# Supplementary material for: Gene and Allele-Specific Expression Underlying the Electric Signal Divergence in African Weakly Electric Fish
Source: Mol Biol Evol. 2024 Feb 15;41(2):msae021. doi: 10.1093/molbev/msae021 (PMC10897887; doi:10.1093/molbev/msae021)
Supplement: msae021_Supplementary_Data [file msae021_supplementary_data.zip › Cheng-MBE-efishtranscriptomes-Supplementary Table 1 Up regulated genes.pdf]

Supplementary Table 1 Genes up-regulated in all species/hybrids in the electric organ relative to sceletal muscle.

ID	Blast Gene	Hightlighs of Predicted Function	Gene Description	Category	Average log2FC	Average Pvalue
maker-ptg000361l-augustus-gene-0.2-mRNA-1	*ACTR3b*	F-actin dynamics / polymerization	ARP3 actin related protein 3 homolog B	cytoskeletal & sarcomeric	3.02	3.1635E-20
snap_masked-ptg000904l-processed-gene-5.5-mRNA-1	*ARPC3*	F-actin dynamics / polymerization	actin related protein 2/3 complex subunit 3	cytoskeletal & sarcomeric	2.01	9.68947E-05
maker-ptg001180l-augustus-gene-13.21-mRNA-1	*ARPC5*	F-actin dynamics / polymerization	actin-related protein 2/3 complex subunit 5	cytoskeletal & sarcomeric	1.99	0.003541395
maker-ptg002072l-snap-gene-6.44-mRNA-1	*CARMIL1*	F-actin dynamics / polymerization	capping protein regulator and myosin 1 linker 1	cytoskeletal & sarcomeric	1.91	0.001040325
maker-ptg000650l-snap-gene-7.49-mRNA-1	*EPPK1*	controls reorganization of intermediate filaments	epiplakin 1	cytoskeletal & sarcomeric	7.74	1.21807E-11
maker-ptg000346l-snap-gene-25.173-mRNA-1	*GSN*	F-actin dynamics / polymerization	gelsolin	cytoskeletal & sarcomeric	6.70	2.7189E-50
maker-ptg001291l-augustus-gene-1.16-mRNA-1	*MYO1d*	unconventional myosin; actin-based motor protein	unconventional myosin-Id	cytoskeletal & sarcomeric	2.16	0.013895309
maker-ptg001182l-snap-gene-5.74-mRNA-1	*MTSS1*	regulation of F-actin dynamics	metastasis suppressor protein 1	cytoskeletal & sarcomeric	4.71	1.09085E-10
maker-ptg000190l-snap-gene-4.78-mRNA-1	*MYH10*	unconventional myosin; actin-based motor protein	myosin-10	cytoskeletal & sarcomeric	1.57	2.93467E-10
maker-ptg001188l-snap-gene-5.24-mRNA-1	*MYL4*	regulatory light chain of myosin	myosin light chain 4	cytoskeletal & sarcomeric	7.12	5.64592E-05
snap_masked-ptg000028l-processed-gene-137.57-mRNA-1	*MYO15a*	unconventional myosin; actin-based motor protein	unconventional myosin-XV	cytoskeletal & sarcomeric	3.68	3.40016E-09
maker-ptg001291l-snap-gene-1.34-mRNA-1	*MYO1d*	unconventional myosin; actin-based motor protein	myosin ID	cytoskeletal & sarcomeric	1.93	0.003762165
maker-ptg001003l-snap-gene-2.16-mRNA-1	*MYO1e*	unconventional myosin; actin-based motor protein	unconventional myosin-Ie	cytoskeletal & sarcomeric	2.63	5.70736E-05
maker-ptg002090l-augustus-gene-35.16-mRNA-1	*MYO3b*	unconventional myosin; actin-based motor protein	myosin-IIIb	cytoskeletal & sarcomeric	3.75	1.42712E-07
maker-ptg000509l-snap-gene-4.4-mRNA-1	*NHS*	regulator of actin remodelling	Nance-Horan syndrome protein	cytoskeletal & sarcomeric	5.30	3.10465E-11
maker-ptg002114l-augustus-gene-0.11-mRNA-1	*PARVG*	regulation of cytoskeleton organization	parvin gamma	cytoskeletal & sarcomeric	3.70	7.51203E-19
snap_masked-ptg000650l-processed-gene-9.23-mRNA-1	*PLEC*	cross-linking and stabilization of cytoskeletal intermediate filaments	plectin	cytoskeletal & sarcomeric	2.62	8.77122E-22
maker-ptg000014l-snap-gene-7.139-mRNA-1	*MAP7d1*	microtubule cytoskeleton organization	MAP7 domain-containing protein 1	cytoskeletal & sarcomeric	8.32	6.90805E-34
snap_masked-ptg000056l-processed-gene-18.17-mRNA-1	*ATP10d*	catalytic component of a P4-ATPase flippase complex	ATPase phospholipid transporting 10D	membrane organization	1.74	0.001111433
maker-ptg000298l-snap-gene-2.25-mRNA-1	*ESYT1*	ER-plasma membrane contact sites	extended synaptotagmin	membrane organization	2.30	3.56639E-09
maker-ptg000317l-snap-gene-0.11-mRNA-1	*CALR3b*	Ca2+-binding chaperone in ER	calreticulin	other	1.38	0.000700663
maker-ptg000316l-snap-gene-4.241-mRNA-1	*CAPN1a*	Ca2+-dependent, non-lysosomal cysteine protease	calpain-1 catalytic subunit	other	3.26	1.5809E-41
maker-ptg000214l-augustus-gene-8.178-mRNA-1	*CAPN5*	Ca2+-dependent, non-lysosomal cysteine protease	calpain 5	other	4.07	3.13077E-28
maker-ptg000886l-augustus-gene-0.134-mRNA-1	*CARHSP1*	regulation of mRNA stability	calcium regulated heat stable protein 1	other	3.01	2.85152E-08
maker-ptg000316l-snap-gene-7.47-mRNA-1	*CRIP3*	metal ion binding activity	cysteine rich protein 3	other	4.90	1.31827E-14
maker-ptg000644l-snap-gene-0.4-mRNA-1	*GDPD4*	enable metal ion binding activity and phosphoric diester hydrolase activity	glycerophosphodiester phosphodiesterase domain containing 4	other	4.98	3.97129E-12
maker-ptg000405l-snap-gene-38.13-mRNA-1	*SMOC2*	secreted calcium-binding protein	SPARC related modular calcium binding 2	other	5.40	1.01506E-17
maker-ptg000070l-snap-gene-5.18-mRNA-1	*FLOT2a*	scaffolding protein within caveolar membranes	flotillin-2a	other	5.77	1.81884E-53
maker-ptg000040l-snap-gene-14.53-mRNA-1	*NAT8l*	catalyzes the synthesis of N-acetylaspartate acid	N-acetyltransferase 8	other	7.97	1.96623E-07
maker-ptg000102l-snap-gene-52.7-mRNA-1	*ST8SIA5*	glycosphingolipid biosynthetic process	ST8 alpha-N-acetyl-neuraminide alpha-2,8-sialyltransferase 5	other	9.67	6.85106E-08
maker-ptg000120l-snap-gene-2.54-mRNA-1	*SYNGR3*	synaptic vesicule membrane protein	synaptogyrin-3	other	10.00	3.33741E-07
maker-ptg000152l-snap-gene-4.203-mRNA-1	*ZDHHC23*	palmitoyltransferase	zinc finger DHHC-type containing 23	other	6.43	5.59154E-07
maker-ptg001827l-augustus-gene-0.22-mRNA-1	*FAT3*	cell-cell adhesion	FAT atypical cadherin 3	other	2.72	0.001238961
snap_masked-ptg000148l-processed-gene-1.18-mRNA-1	*FAT4*	cell adhesion molecule	FAT atypical cadherin 4	other	3.97	3.45897E-11
maker-ptg001421l-augustus-gene-5.0-mRNA-1	*JCAD*	cell adhesion	junctional protein associated with coronary artery disease	other	4.28	3.35791E-67
maker-ptg001427l-augustus-gene-38.97-mRNA-1	*KCTD9*	Substrate-specific adapter of a cullin-based E3 ubiquitin-protein ligase complex	potassium channel tetramerization domain containing 9	other	1.77	3.05442E-05
maker-ptg000361l-snap-gene-40.32-mRNA-1	*LAMA1*	extracellular matrix protein	laminin subunit alpha-1	other	5.73	3.75043E-57
maker-ptg000187l-augustus-gene-16.29-mRNA-1	*MMP28*	extracellular matrix protein	matrix metallopeptidase 28	other	4.82	6.22492E-59
maker-ptg000964l-snap-gene-1.24-mRNA-1	*NCAM1*	cell adhesion molecule	neuronal cell adhesion molecule	other	4.84	2.16487E-24
maker-ptg000718l-snap-gene-13.14-mRNA-1	*NDFIP2*	activates HECT domain-containing E3 ubiquitin-protein ligases	NEDD4 family-interacting protein 2	other	8.81	1.42211E-12
maker-ptg000879l-snap-gene-1.65-mRNA-1	*TMIGD1*	cell adhesion molecule	transmembrane and immunoglobulin domain containing 1	other	9.81	1.75298E-07
maker-ptg001236l-augustus-gene-24.16-mRNA-1	*TRH*	tripeptide hypothalamic regulatory hormone	thyrotropin releasing hormone	other	9.79	5.36476E-17
maker-ptg000774l-augustus-gene-4.234-mRNA-1	*CTNNAL1*	modulation the Rho pathway signaling	catenin alpha like 1	signaling	5.91	2.6796E-49
maker-ptg000049l-snap-gene-23.20-mRNA-1	*FGF12*	regulation of voltage-gated sodium channels	fibroblast growth factor 12	signaling	8.72	5.84977E-17
maker-ptg000028l-augustus-gene-28.23-mRNA-1	*HEG1*	calcium ion binding activity	protein HEG homolog 1	signaling	8.38	8.41625E-14
maker-ptg000159l-augustus-gene-6.0-mRNA-1	*PCP4*	modulator of calcium-binding by calmodulin	calmodulin regulator protein PCP4	signaling	6.64	4.01068E-05
maker-ptg000869l-snap-gene-20.89-mRNA-1	*PVALB9*	cytosolic Ca2+-binding protein of the EF-hand superfamily	parvalbumin, thymic	signaling	8.51	1.38958E-09
maker-ptg000215l-snap-gene-13.31-mRNA-1	*S100b*	cytosolic Ca2+-binding protein of the EF-hand superfamily	S100 calcium binding protein B	signaling	8.14	1.01224E-22
snap_masked-ptg001241l-processed-gene-1.62-mRNA-1	*KL*	may be involved in the regulation of calcium and phosphorus homeostasis	klotho	signaling	5.39	0.000196154
maker-ptg000830l-snap-gene-5.84-mRNA-1	*SEMA5a*	ligand for receptor PLXNB3	semaphorin-5A	signaling	9.17	2.9895E-15
maker-ptg000408l-augustus-gene-0.0-mRNA-1	*ANXA4*	annexin family of calcium-dependent phospholipid binding proteins	annexin A4	signaling	5.55	6.72209E-60
maker-ptg000084l-augustus-gene-28.11-mRNA-1	*CAMK1d*	Ca2+/calmodulin-dependent protein kinase	calcium/calmodulin dependent protein kinase ID	signaling	3.18	0.000145331
maker-ptg000237l-snap-gene-9.30-mRNA-1	*CAMK1g*	Ca2+/calmodulin-dependent protein kinase	calcium/calmodulin-dependent protein kinase type 1D	signaling	4.56	0.000111371
maker-ptg000008l-augustus-gene-0.0-mRNA-1	*CHRNB4*	neuronal acetylcholine receptor subunit alpha; nonselective cation channel	neuronal acetylcholine receptor subunit beta-4	signaling	6.28	2.08134E-05
maker-ptg001610l-snap-gene-4.137-mRNA-1	*GRIK3*	ionotropic glutamate receptor	glutamate ionotropic receptor kainate type subunit 3	signaling	6.15	5.72433E-05
maker-ptg001029l-snap-gene-17.22-mRNA-1	*GRIN2a*	ionotropic glutamate receptor	glutamate receptor ionotropic, NMDA 2A	signaling	6.25	0.001464578
maker-ptg000897l-snap-gene-2.61-mRNA-1	*GRINA*	negative regulation of apoptotic signaling pathway	glutamate ionotropic receptor NMDA type subunit associated protein 1	signaling	3.24	7.33209E-61
maker-ptg000068l-augustus-gene-18.3-mRNA-1	*ITPR1*	InsP3-dependent ER Ca2+ channel	inositol 1,4,5-trisphosphate receptor type 1	signaling	4.07	9.87654E-18
maker-ptg000838l-snap-gene-3.218-mRNA-1	*NDRG3*	predicted to be involved in signal transduction	N-myc downstream-regulated gene 3 protein	signaling	11.02	7.44456E-08
snap_masked-ptg001427l-processed-gene-2.9-mRNA-1	*P2RY2*	receptor for ATP and UTP coupled to G-proteins that activate a phosphatidylinositol-calcium second messenger system	P2Y purinoceptor 2	signaling	3.78	0.001473877
maker-ptg000267l-snap-gene-2.92-mRNA-1	*PIEZO2*	mechanosensitive ion channel	piezo type mechanosensitive ion channel component 2	signaling	1.89	1.03736E-06
maker-ptg001424l-augustus-gene-5.145-mRNA-1	*RET*	receptor tyrosine-protein kinase	ret proto-oncogene	signaling	10.14	2.32382E-12
maker-ptg001563l-augustus-gene-5.35-mRNA-1	*SGK1*	serine/threonine-protein kinase	serine/threonine-protein kinase Sgk1	signaling	7.79	6.63267E-38
maker-ptg000926l-snap-gene-6.19-mRNA-1	*TRPV1*	transient receptor potential family of ion channels; nociception	transient receptor potential cation channel subfamily V member 1	signaling	2.43	0.005919225
maker-ptg001088l-snap-gene-0.7-mRNA-1	*SIX2a*	target ARE promoter elements in Na+/K+ adenosine triphosphatases	SIX homeobox 2	transcription factor	3.05	1.22103E-27
maker-ptg000783l-snap-gene-6.78-mRNA-1	*HEY1*	developing cardiac conduction pathway	hes related family bHLH transcription factor with YRPW motif 1	transcription factor	6.02	1.5325E-13
maker-ptg000149l-augustus-gene-2.87-mRNA-1	*ETV5*	transcription factor	ETS translocation variant 5	transcription factor	5.11	1.99403E-55
snap_masked-ptg000737l-processed-gene-6.55-mRNA-1	*FOXL2*	regulate distinct female sex determining pathways	forkhead box protein L2	transcription factor	8.52	4.14855E-07
maker-ptg001740l-augustus-gene-1.112-mRNA-1	*KLF5*	rebalance potassium channels	Krueppel-like factor 5	transcription factor	8.39	5.04913E-06
maker-ptg000008l-snap-gene-10.43-mRNA-1	*MEF2a*	transcriptional activator for numerous muscle-specific genes	myocyte-specific enhancer factor 2A	transcription factor	3.85	3.78175E-50
maker-ptg001270l-snap-gene-47.16-mRNA-1	*MEF2b*	transcriptional activator for numerous muscle-specific genes	myocyte-specific enhancer factor 2B	transcription factor	6.92	8.45833E-68
maker-ptg000345l-snap-gene-4.29-mRNA-1	*ANO10*	calcium-activated chloride channel	anoctamin 10	transmembrane ion transport	2.37	1.21792E-13
maker-ptg000406l-snap-gene-3.4-mRNA-1	*ANO5*	calcium-activated chloride channel	anoctamin 5	transmembrane ion transport	2.76	3.56495E-17
maker-ptg000979l-snap-gene-12.179-mRNA-1	*ANO6*	calcium-activated nonselective cation (SCAN) channel which acts as a regulator of phospholipid scrambling	anoctamin 6	transmembrane ion transport	1.82	0.000445473
maker-ptg000970l-augustus-gene-2.127-mRNA-1	*ATP1a1*	Na/K-ATPase α-subunit	sodium/potassium-transporting ATPase subunit alpha-1	transmembrane ion transport	10.55	2.18943E-05
snap_masked-ptg001156l-processed-gene-0.19-mRNA-1	*ATP1a2a*	Na/K-ATPase α-subunit	sodium/potassium-transporting ATPase subunit alpha-2	transmembrane ion transport	3.07	2.63171E-12
maker-ptg001047l-snap-gene-1.63-mRNA-1	*ATP1b1a*	Na/K-ATPase β-subunit	ATPase NA+/K+ transporting beta 1a	transmembrane ion transport	6.59	3.63255E-59
maker-ptg000509l-snap-gene-9.39-mRNA-1	*ATP1b1b*	Na/K-ATPase β-subunit	ATPase NA+/K+ transporting beta 1b	transmembrane ion transport	4.51	2.06472E-06
maker-ptg000993l-snap-gene-3.19-mRNA-1	*ATP2a2*	sarcoplasmic/endoplasmic reticulum calcium ATPase 2	sarcoplasmic/endoplasmic reticulum calcium ATPase 2	transmembrane ion transport	1.90	5.79784E-08
snap_masked-ptg000904l-processed-gene-1.73-mRNA-1	*ATP2a2b*	sarcoplasmic/endoplasmic reticulum calcium ATPase 2	sarcoplasmic/endoplasmic reticulum calcium ATPase 2b	transmembrane ion transport	2.72	5.65072E-08
maker-ptg000084l-augustus-gene-36.7-mRNA-1	*ATP2b1a*	plasma membrane calcium-transporting ATPase 1	plasma membrane calcium-transporting ATPase 1a	transmembrane ion transport	2.96	4.08993E-15
maker-ptg000084l-snap-gene-36.24-mRNA-1	*ATP2b1b*	plasma membrane calcium-transporting ATPase 1	plasma membrane calcium-transporting ATPase 1b	transmembrane ion transport	3.66	1.07062E-27
maker-ptg000135l-augustus-gene-78.38-mRNA-1	*ATP2b3*	ATPase plasma membrane Ca2+ transporting 3	ATPase plasma membrane Ca2+ transporting 3	transmembrane ion transport	5.65	1.02377E-05
maker-ptg000347l-snap-gene-10.66-mRNA-1	*ATP2c1*	secretory pathway Ca2+-ATPase	ATPase secretory pathway Ca2+ transporting 1	transmembrane ion transport	2.14	0.000146327
maker-ptg000032l-augustus-gene-1.26-mRNA-1	*ATP6ap2*	subunit of vacuolar-type H+-ATPase	ATPase H+ transporting accessory protein 2	transmembrane ion transport	1.78	2.28333E-09
maker-ptg000501l-augustus-gene-3.39-mRNA-1	*ATP6v1f*	subunit of vacuolar-type H+-ATPase	ATPase H+ transporting V1 subunit F	transmembrane ion transport	1.48	1.99856E-09
maker-ptg001427l-snap-gene-4.23-mRNA-1	*CACNA1b*	calcium channel subunit	voltage-dependent N-type calcium channel subunit alpha-1B	transmembrane ion transport	5.19	2.70467E-08

maker-ptg001182l-snap-gene-8.79-mRNA-1	*CNGB1*	nonselective cation channel	cyclic nucleotide-gated cation channel beta-1	transmembrane ion transport	5.76	2.75381E-05
snap_masked-ptg000084l-processed-gene-43.23-mRNA-1	*CRACR2aa*	Ca2+ binding protein that is a key regulator of CRAC channel-mediated SOCE; adaptor protein for cytoplasic dynein	calcium release activated channel regulator 2Aa	transmembrane ion transport	3.67	0.001092994
maker-ptg001069l-augustus-gene-3.65-mRNA-1	*GABRA1*	subunit of ligand-gated chloride channel	gamma-aminobutyric acid receptor subunit alpha-1	transmembrane ion transport	5.44	1.17436E-05
snap_masked-ptg000509l-processed-gene-7.11-mRNA-1	*GABRG3*	subunit of ligand-gated chloride channel	gamma-aminobutyric acid receptor subunit gamma-3	transmembrane ion transport	2.78	0.000289984
snap_masked-ptg002179l-processed-gene-1.70-mRNA-1	*GLRBB*	ligand-gated chloride channel	glycine receptor, beta b	transmembrane ion transport	9.03	2.58552E-29
maker-ptg000559l-snap-gene-2.76-mRNA-1	*HCN2*	hyperpolarization-activated ion channel	hyperpolarization activated cyclic nucleotide gated potassium and sodium channel 2	transmembrane ion transport	3.65	1.79576E-09
maker-ptg000028l-snap-gene-81.10-mRNA-1	*KCNA7a_1*	voltage-gated potassium channel	potassium voltage-gated channel subfamily A member 7a	transmembrane ion transport	4.60	2.58472E-11
maker-ptg000028l-snap-gene-81.8-mRNA-1	*KCNA7a_2*	voltage-gated potassium channel	potassium voltage-gated channel subfamily A member 7a	transmembrane ion transport	8.27	3.55527E-12
maker-ptg001427l-snap-gene-13.20-mRNA-1	*KCNIP3*	voltage-gated potassium channel	calsenilin	transmembrane ion transport	5.11	0.000993571
maker-ptg000265l-est_gff_est2genome-gene-6.33-mRNA-1	*KCNJ2*	inwardly rectifying potassium channel	inward rectifier potassium channel 2	transmembrane ion transport	5.53	1.12219E-20
maker-ptg000830l-augustus-gene-5.123-mRNA-1	*KCNJ9*	inwardly rectifying potassium channel	G protein-activated inward rectifier potassium channel 3	transmembrane ion transport	5.77	2.63245E-10
snap_masked-ptg001118l-processed-gene-0.13-mRNA-1	*KCNK2*	potassium two pore domain channel	potassium channel subfamily K member 2	transmembrane ion transport	6.15	9.23801E-14
maker-ptg000697l-snap-gene-6.109-mRNA-1	*KCNQ5*	voltage-gated potassium channel	potassium voltage-gated channel subfamily Q member 5	transmembrane ion transport	5.94	0.000938938
maker-ptg001106l-augustus-gene-0.48-mRNA-1	*MCOLN1*	intracellular cation channel	mucolipin-1	transmembrane ion transport	6.37	6.01855E-26
maker-ptg001057l-snap-gene-3.24-mRNA-1	*MCOLN3*	intracellular cation channel	mucolipin-3	transmembrane ion transport	5.68	4.30182E-06
maker-ptg000253l-augustus-gene-20.10-mRNA-1	*SCN1ba*	voltage-gated sodium channel beta-subunit (regulatory)	sodium channel subunit beta-1	transmembrane ion transport	3.84	1.29832E-07
maker-ptg001188l-snap-gene-6.4-mRNA-1	*SCN4aa*	voltage-gated sodium channel alpha-subunit	sodium channel protein type 4 subunit alpha A	transmembrane ion transport	10.98	1.27373E-11
maker-ptg002239l-snap-gene-5.5-mRNA-1	*SCN4b*	voltage-gated sodium channel	sodium voltage-gated channel beta subunit 4	transmembrane ion transport	5.34	8.18062E-47
maker-ptg000477l-augustus-gene-1.10-mRNA-1	*VDAC1*	ion channel in the outer mitochondrial membrane and also the outer cell membrane	voltage-dependent anion-selective channel protein 1	transmembrane ion transport	2.69	2.46626E-06
maker-ptg000051l-snap-gene-132.157-mRNA-1	*TMEM206*	proton-activated chloride channel	transmembrane protein 206	transmembrane ion transport	1.98	6.00534E-10
maker-ptg001300l-snap-gene-4.198-mRNA-1	*TMEM63c*	Ca2+ permeable cation channel at ER/mitochondria contact sites	transmembrane protein 63C	transmembrane ion transport	4.00	0.001858763
maker-ptg000187l-augustus-gene-15.2-mRNA-1	*TMEM120a*	mechanosensing ion channel	transmembrane protein 120A	transmembrane ion transport	6.05	2.33605E-44
maker-ptg000148l-snap-gene-9.4-mRNA-1	*SLC24a2*	calcium, potassium:sodium antiporter	solute carrier family 24 member 2	transmembrane ion transport	9.06	1.1057E-37
snap_masked-ptg000102l-processed-gene-12.46-mRNA-1	*SLC4a4*	sodium bicarbonate cotransporter	solute carrier family 4 member 4	transmembrane ion transport	5.67	1.3753E-11
snap_masked-ptg000004l-processed-gene-3.9-mRNA-1	*SLC8a1a*	sodium/calcium exchanger	solute carrier family 8 member 1a	transmembrane ion transport	5.01	3.00748E-13
maker-ptg002207l-snap-gene-0.59-mRNA-1	*ABCA2*	probable lipid transporter that modulates cholesterol sequestration in the late endosome/lysosome	ATP binding cassette subfamily A member 2	transmembrane ion transport	2.21	1.89872E-10
maker-ptg001844l-snap-gene-4.2-mRNA-1	*ABCB1*	ABC transporter; translocates drugs and phospholipids across the membrane	ATP binding cassette subfamily B member 1	transmembrane ion transport	4.72	0.000481822
maker-ptg001436l-snap-gene-0.52-mRNA-1	*ABCG2*	ABC transporter of broad substrate specificity	ATP-binding cassette sub-family G member 2	transmembrane ion transport	2.24	0.008846064
maker-ptg000326l-snap-gene-3.64-mRNA-1	*SLC13a1*	sodium:sulfate symporter	solute carrier family 13 member 1	transmembrane ion transport	3.41	3.99214E-05
snap_masked-ptg000878l-processed-gene-2.22-mRNA-1	*SLC17a5*	membrane transporter that exports free sialic acids	solute carrier family 17 member 5	transmembrane ion transport	3.88	1.23265E-08
maker-ptg001427l-snap-gene-14.26-mRNA-1	*SLC23a2*	sodium/ascorbate cotransporter	solute carrier family 23 member 2	transmembrane ion transport	2.45	3.35477E-05
snap_masked-ptg001837l-processed-gene-1.59-mRNA-1	*SLC25a23*	calcium-dependent mitochondrial solute carrier on the inner mitochondrial membrane	solute carrier family 25 member 23	transmembrane ion transport	5.13	0.000247848
maker-ptg000102l-augustus-gene-64.1-mRNA-1	*SLC25a25b*	calcium-binding mitochondrial carrier on the inner mitochondrial membrane	solute carrier family 25 member 25b	transmembrane ion transport	2.65	0.003639241
maker-ptg000184l-augustus-gene-2.1-mRNA-1	*SLC5a7*	sodium ion- and chloride ion-dependent high-affinity transporter that mediates choline uptake	solute carrier family 5 member 7	transmembrane ion transport	5.91	0.000293221
maker-ptg000175l-augustus-gene-7.91-mRNA-1	*SLC5a9*	sodium/glucose cotransporter 4	solute carrier family 5 member 9	transmembrane ion transport	2.80	6.19949E-11
maker-ptg001587l-snap-gene-9.16-mRNA-1	*SLC6a17*	sodium-dependent vesicular transporter selective for proline, glycine, leucine and alanine	solute carrier family 6 member 17	transmembrane ion transport	8.15	5.51716E-10
maker-ptg000800l-augustus-gene-2.11-mRNA-1	*SLC6a2*	sodium- and chloride-dependent transport of norepinephrine	solute carrier family 6 member 2	transmembrane ion transport	5.50	3.30148E-15
maker-ptg001004l-snap-gene-4.176-mRNA-1	*SLC6a6*	sodium- and chloride-dependent transport of taurine and beta-alanine	solute carrier family 6 member 6	transmembrane ion transport	4.10	7.32652E-09
