# Supplementary material for: Gene and Allele-Specific Expression Underlying the Electric Signal Divergence in African Weakly Electric Fish
Source: Mol Biol Evol. 2024 Feb 15;41(2):msae021. doi: 10.1093/molbev/msae021 (PMC10897887; doi:10.1093/molbev/msae021)
Supplement: msae021_Supplementary_Data [file msae021_supplementary_data.zip › Cheng-MBE-efishtranscriptomes-Supplementary Table 2 Down regulatedgenes.pdf]

Supplementary Table 2 Genes down-regulated in all species/hybrids in the electric organ relative to sceletal muscle.

ID	Blast Gene	Highlights of Predicted Function	Gene Description	Category	Average log2FC	Average Pvalue
snap_masked-ptg000308l-processed-gene-8.27-mRNA-1	*ABI2a*	regulator of actin cytoskeleton dynamics	abl interactor 2a	cytoskeletal & sarcomeric	-1.94	0.004180823
maker-ptg000512l-snap-gene-2.123-mRNA-1	*ABLIM1b*	binds to actin filaments and mediates interactions between actin and cytoplasmic targets	actin-binding LIM protein 1	cytoskeletal & sarcomeric	-2.88	1.69993E-09
maker-ptg001458l-snap-gene-2.2-mRNA-1	*ABRaa*	muscle specific actin-binding protein; involved in skeletal muscle hypertrophy and atrophy	actin-binding Rho-activating protein	cytoskeletal & sarcomeric	-5.52	1.11159E-06
maker-ptg001416l-augustus-gene-5.37-mRNA-1	*ABRab*	muscle specific actin-binding protein; involved in skeletal muscle hypertrophy and atrophy	actin binding Rho activating protein	cytoskeletal & sarcomeric	-5.37	5.05532E-07
maker-ptg000306l-augustus-gene-0.39-mRNA-1	*ACTA1*	actin isoform, striated muscle	actin, alpha 1, skeletal muscle	cytoskeletal & sarcomeric	-5.17	9.36728E-19
maker-ptg001070l-augustus-gene-5.71-mRNA-1	*ACTB1*	actin isoform, cytoplasmic	actin, cytoplasmic 1	cytoskeletal & sarcomeric	-1.95	0.00022477
maker-ptg000171l-snap-gene-9.31-mRNA-1	*ACTC1a*	actin isoform, striated muscle	actin, alpha cardiac muscle 1a	cytoskeletal & sarcomeric	-8.11	8.1603E-05
snap_masked-ptg002519l-processed-gene-0.5-mRNA-1	*ACTC1b*	actin isoform, striated muscle	actin, alpha cardiac muscle 1b	cytoskeletal & sarcomeric	-4.70	0.004466918
maker-ptg000067l-snap-gene-17.2-mRNA-1	*ACTC2*	actin isoform, striated muscle	actin, alpha, cardiac muscle 2	cytoskeletal & sarcomeric	-7.94	0.006969056
maker-ptg000501l-snap-gene-3.13-mRNA-1	*CAPZA2*	F-actin binding protein	F-actin-capping protein subunit alpha-2	cytoskeletal & sarcomeric	-2.35	1.10876E-11
maker-ptg000693l-snap-gene-21.128-mRNA-1	*CAPZB*	F-actin binding protein	F-actin-capping protein subunit beta	cytoskeletal & sarcomeric	-1.32	9.88642E-06
maker-ptg000196l-augustus-gene-4.0-mRNA-1	*KY*	cytoskeleton-associated protease required for normal muscle growth	kyphoscoliosis peptidase	cytoskeletal & sarcomeric	-7.80	0.000264434
maker-ptg000007l-snap-gene-2.65-mRNA-1	*MYBPC1*	thick filament-associated protein located in the crossbridge region of vertebrate striated muscle A bands	myosin binding protein C, slow type	cytoskeletal & sarcomeric	-6.60	0.000728724
snap_masked-ptg000028l-processed-gene-83.4-mRNA-1	*MYBPC2*	thick filament-associated protein located in the crossbridge region of vertebrate striated muscle A bands	myosin-binding protein C, fast-type	cytoskeletal & sarcomeric	-6.01	0.001321102
maker-ptg000333l-augustus-gene-6.128-mRNA-1	*MYBPC3*	thick filament-associated protein located in the crossbridge region of vertebrate striated muscle A bands	myosin-binding protein C, cardiac-type	cytoskeletal & sarcomeric	-8.23	4.50551E-05
maker-ptg000962l-augustus-gene-12.1-mRNA-1	*MYBPH*	binds to myosin; probably involved in interaction with thick myofilaments in the A-band	myosin-binding protein H	cytoskeletal & sarcomeric	-4.84	0.003814769
maker-ptg000085l-augustus-gene-17.7-mRNA-1	*MYH2*	unconventional myosin; actin-based motor protein	myosin heavy chain, fast skeletal muscle	cytoskeletal & sarcomeric	-8.17	5.93451E-19
snap_masked-ptg000407l-processed-gene-2.145-mRNA-1	*MYH1*	myosin heavy chain beta isoform expressed primarily in the heart, but also in skeletal muscles	myosin 1	cytoskeletal & sarcomeric	-8.60	4.34569E-06
maker-ptg000133l-augustus-gene-18.18-mRNA-1	*MYL1*	non-regulatory myosin light chain	myosin light chain 1	cytoskeletal & sarcomeric	-5.57	1.66255E-08
maker-ptg001175l-snap-gene-0.158-mRNA-1	*MYL2*	myosin regulatory light chain 2	myosin regulatory light chain 2, cardiac muscle	cytoskeletal & sarcomeric	-6.12	0.000536607
maker-ptg000187l-snap-gene-16.83-mRNA-1	*MYL2b*	myosin regulatory light chain 2	myosin regulatory light chain 2B, cardiac muscle	cytoskeletal & sarcomeric	-8.19	5.84804E-05
maker-ptg000100l-snap-gene-11.24-mRNA-1	*MYL4*	myosin regulatory light chain	myosin light chain 4	cytoskeletal & sarcomeric	-7.66	0.001195604
maker-ptg000135l-snap-gene-52.42-mRNA-1	*MYLK2*	myosin light chain kinase	myosin light chain kinase 2	cytoskeletal & sarcomeric	-4.77	0.000103916
maker-ptg000441l-augustus-gene-6.2-mRNA-1	*MYO16*	unconventional myosin; actin-based motor protein	unconventional myosin-XVI	cytoskeletal & sarcomeric	-4.85	0.006179819
maker-ptg000256l-snap-gene-3.25-mRNA-1	*MYO18a*	unconventional myosin; actin-based motor protein	unconventional myosin-XVIIIa	cytoskeletal & sarcomeric	-4.19	1.94298E-13
maker-ptg000960l-augustus-gene-0.14-mRNA-1	*MYO18b*	unconventional myosin; actin-based motor protein	myosin XVIIIB	cytoskeletal & sarcomeric	-3.94	8.08729E-15
maker-ptg000193l-snap-gene-2.47-mRNA-1	*MYOM1*	major component of the vertebrate myofibrillar M band	myomesin 1	cytoskeletal & sarcomeric	-7.91	1.13806E-07
maker-ptg000110l-augustus-gene-2.12-mRNA-1	*MYOM2*	major component of the vertebrate myofibrillar M band	myomesin 2	cytoskeletal & sarcomeric	-4.02	3.46432E-14
maker-ptg000405l-snap-gene-31.60-mRNA-1	*MYOZ1*	involved in linking Z-disk proteins	myozenin 1	cytoskeletal & sarcomeric	-6.71	6.15331E-07
maker-ptg000314l-augustus-gene-4.2-mRNA-1	*MYOZ2*	involved in linking Z-disk proteins	myozenin 2	cytoskeletal & sarcomeric	-7.19	0.001878985
maker-ptg000182l-snap-gene-3.82-mRNA-1	*MYOZ3*	involved in linking Z-disk proteins	myozenin 3	cytoskeletal & sarcomeric	-6.59	0.001769106
maker-ptg001112l-snap-gene-5.13-mRNA-1	*NEB*	binds and stabilize F-actin in the sarcomere	nebulin	cytoskeletal & sarcomeric	-7.40	0.000736895
maker-ptg000512l-snap-gene-3.14-mRNA-1	*NRAP*	may be involved in anchoring the terminal actin filaments in the myofibril to the membrane	nebulin related anchoring protein	cytoskeletal & sarcomeric	-3.92	1.29313E-19
maker-ptg002114l-snap-gene-0.37-mRNA-1	*PARVB*	adapter protein involved in the reorganization of the actin cytoskeleton	parvin beta	cytoskeletal & sarcomeric	-5.41	3.02009E-05
maker-ptg000572l-snap-gene-14.41-mRNA-1	*SMYHC1*	myosin heavy chain; actin-based motor protein	slow myosin heavy chain 1	cytoskeletal & sarcomeric	-7.57	0.004522565
maker-ptg000572l-augustus-gene-14.18-mRNA-1	*SMYHC2*	myosin heavy chain; actin-based motor protein	slow myosin heavy chain 2	cytoskeletal & sarcomeric	-5.20	0.001635808
maker-ptg000135l-augustus-gene-79.30-mRNA-1	*TNNC1*	component of troponin complex; regulation of muscle contraction	troponin C, slow skeletal and cardiac muscles	cytoskeletal & sarcomeric	-7.24	0.000307439
maker-ptg000135l-snap-gene-52.7-mRNA-1	*TNNC2*	component of troponin complex; regulation of muscle contraction	troponin C, skeletal muscle	cytoskeletal & sarcomeric	-4.81	5.01121E-11
maker-ptg000223l-snap-gene-2.48-mRNA-1	*TNNI2*	component of troponin complex; regulation of muscle contraction	troponin I, fast skeletal muscle	cytoskeletal & sarcomeric	-7.27	3.13868E-23
maker-ptg000445l-snap-gene-11.25-mRNA-1	*TPM1*	actin-binding protein; in association with the troponin complex involved in the striated muscle contraction	tropomyosin alpha-1 chain	cytoskeletal & sarcomeric	-4.81	6.55146E-15
maker-ptg001553l-augustus-gene-0.54-mRNA-1	*TPM2*	actin-binding protein; in association with the troponin complex involved in the striated muscle contraction	tropomyosin beta chain	cytoskeletal & sarcomeric	-6.67	1.53935E-06
maker-ptg001563l-snap-gene-12.53-mRNA-1	*TPM3*	actin-binding protein; in association with the troponin complex involved in the striated muscle contraction	tropomyosin alpha-3 chain	cytoskeletal & sarcomeric	-3.70	4.35071E-06
maker-ptg000555l-augustus-gene-11.55-mRNA-1	*TTN*	sarcomere organization	titin	cytoskeletal & sarcomeric	-2.95	3.70566E-07
snap_masked-ptg000176l-processed-gene-4.104-mRNA-1	*HSPB11*	disassembly of the sarcomeres	heat shock protein beta-11	cytoskeletal & sarcomeric	-4.89	5.99473E-07
maker-ptg001400l-snap-gene-3.115-mRNA-1	*CAPN3*	muscle-specific calpain; calcium-activated non-lysosomal thiol-protease	calpain 3	other	-4.51	3.46551E-17
maker-ptg000222l-snap-gene-9.11-mRNA-1	*CDH20*	calcium-dependent cell adhesion protein	cadherin-20	other	-3.77	0.000127412
maker-ptg000061l-augustus-gene-13.21-mRNA-1	*CDH26*	calcium-dependent cell adhesion protein	cadherin-like protein 26	other	-2.83	0.005372135
maker-ptg000534l-snap-gene-5.169-mRNA-1	*KCMF1*	E3 ubiquitin-protein ligase	potassium channel modulatory factor 1	other	-1.41	7.97951E-07
snap_masked-ptg002090l-processed-gene-16.20-mRNA-1	*ACOT12*	fatty acid metabolic process	acyl-CoA thioesterase 12	other	-7.79	0.00346148
maker-ptg001966l-augustus-gene-10.17-mRNA-1	*CKM*	reversibly catalyzes the transfer of phosphate between ATP and various phosphogens	creatine kinase M-type	other	-8.43	5.01097E-09
maker-ptg000393l-augustus-gene-30.5-mRNA-1	*CKMT1a*	reversibly catalyzes the transfer of phosphate between ATP and various phosphogens	creatine kinase U-type, mitochondrial	other	-7.81	4.06172E-07
maker-ptg000323l-snap-gene-1.83-mRNA-1	*EPN2*	interacts with clathrin	epsin-2	other	-7.50	1.26567E-05
maker-ptg000299l-snap-gene-6.20-mRNA-1	*GLS2B*	glutaminase activity	glutaminase kidney isoform, mitochondrial	other	-7.18	1.23041E-05
snap_masked-ptg001143l-processed-gene-2.56-mRNA-1	*IGFN1*	cell adhesion	immunoglobulin-like and fibronectin type III domain-containing protein 1	other	-7.80	5.98234E-36
maker-ptg000487l-snap-gene-5.15-mRNA-1	*LDHA*	catalyzes the conversion of L-lactate and NAD to pyruvate and NADH in the final step of anaerobic glycolysis	L-lactate dehydrogenase A chain	other	-7.47	0.000293053
maker-ptg000254l-augustus-gene-6.0-mRNA-1	*TECR*	involved in both the production of very long-chain fatty acids	very-long-chain enoyl-CoA reductase	other	-7.82	0.000632574
maker-ptg000665l-snap-gene-7.139-mRNA-1	*MYOC*	secreted glycoprotein regulating the activation of different signaling pathways in adjacent cells	myocilin	signaling	-2.10	0.00200062
maker-ptg000558l-snap-gene-4.101-mRNA-1	*CALM3*	calcium-binding EF-hand protein	calmodulin 3	signaling	-2.84	8.29055E-05
snap_masked-ptg000869l-processed-gene-20.110-mRNA-1	*PVALB2*	Ca2+-binding protein of the EF-hand superfamily	parvalbumin-2	signaling	-8.61	0.000478414
maker-ptg000974l-augustus-gene-10.59-mRNA-1	*PVALB4*	Ca2+-binding protein of the EF-hand superfamily	parvalbumin 4	signaling	-6.32	0.000158436
maker-ptg002802l-snap-gene-0.11-mRNA-1	*PVALB7*	Ca2+-binding protein of the EF-hand superfamily	parvalbumin 7	signaling	-8.33	5.15658E-05
maker-ptg000481l-snap-gene-2.8-mRNA-1	*ADRB2*	beta-2-adrenergic receptor	adrenoceptor beta 2	signaling	-4.35	1.00955E-12
maker-ptg000135l-augustus-gene-82.84-mRNA-1	*GPR173*	G-protein coupled receptor	probable G-protein coupled receptor 173	signaling	-8.04	0.00013504
maker-ptg001004l-augustus-gene-9.10-mRNA-1	*SBK1*	serine/threonine kinase activity	serine/threonine-protein kinase SBK1	signaling	-6.93	0.00015683
maker-ptg000253l-augustus-gene-58.60-mRNA-1	*MYOCD*	smooth muscle cells and cardiac muscle cells-specific transcriptional factor	myocardin	transcription factor	-3.89	1.03704E-11
maker-ptg000085l-snap-gene-26.0-mRNA-1	*MYOG*	muscle-specific transcription factor	myogenin	transcription factor	-2.73	1.67587E-05
maker-ptg000171l-augustus-gene-6.26-mRNA-1	*ANO1*	calcium-activated chloride channel	anoctamin 1	transmembrane ion transport	-3.40	1.225E-17
maker-ptg000852l-snap-gene-16.100-mRNA-1	*ATP1b3a*	Na/K-ATPase β-subunit	ATPase Na+/K+ transporting subunit beta 3a	transmembrane ion transport	-4.10	1.54943E-19
maker-ptg000600l-augustus-gene-1.19-mRNA-1	*ATP2a1*	sarcoplasmic/endoplasmic reticulum calcium ATPase 1	sarcoplasmic/endoplasmic reticulum calcium ATPase 1	transmembrane ion transport	-7.30	3.1585E-07
maker-ptg000474l-snap-gene-6.56-mRNA-1	*ATP2a2a*	sarcoplasmic/endoplasmic reticulum calcium ATPase 2	sarcoplasmic/endoplasmic reticulum calcium ATPase 2a	transmembrane ion transport	-7.66	0.000724467
maker-ptg000085l-snap-gene-5.119-mRNA-1	*ATP2b2*	plasma membrane Ca2+ transporting ATPase 2	ATPase plasma membrane Ca2+ transporting 2	transmembrane ion transport	-2.46	0.002679005
maker-ptg000718l-snap-gene-53.80-mRNA-1	*ATP5f1b*	subunit of mitochondrial ATP synthase	ATP synthase subunit beta, mitochondrial	transmembrane ion transport	-1.81	2.19442E-06
maker-ptg000237l-snap-gene-5.34-mRNA-1	*CACNA1s*	voltage-gated calcium channel	dihydropyridine-sensitive L-type skeletal muscle calcium channel subunit alpha-1	transmembrane ion transport	-6.87	3.16021E-05
maker-ptg000085l-augustus-gene-14.68-mRNA-1	*CACNA2d1*	voltage-gated calcium channel	voltage-dependent calcium channel subunit alpha-2/delta-2	transmembrane ion transport	-4.46	1.86424E-11
maker-ptg000202l-snap-gene-1.0-mRNA-1	*CACNG1a*	voltage-gated calcium channel	voltage-dependent calcium channel gamma-1 subunit	transmembrane ion transport	-4.04	4.43468E-11
maker-ptg000763l-augustus-gene-3.165-mRNA-1	*CALHM6*	pore-forming subunit of a voltage-gated ion channel	calcium homeostasis modulator family member 6	transmembrane ion transport	-3.12	1.19758E-11
maker-ptg001156l-augustus-gene-8.0-mRNA-1	*CASQ1a*	calcium-binding protein in SR	calsequestrin-1a	transmembrane ion transport	-7.32	4.86051E-08
maker-ptg000021l-augustus-gene-6.30-mRNA-1	*CASQ1b*	calcium-binding protein in SR	calsequestrin-1b	transmembrane ion transport	-3.71	9.95212E-15
maker-ptg000650l-augustus-gene-22.51-mRNA-1	*CLCN1*	voltage-dependent chloride channel; important for repolarization of skeletal muscle cells after muscle contraction	chloride channel protein 1	transmembrane ion transport	-7.56	6.86007E-07
snap_masked-ptg000633l-processed-gene-25.11-mRNA-1	*KCNA1b*	voltage-gated potassium channel	potassium voltage-gated channel subfamily A member 1b	transmembrane ion transport	-6.39	6.20958E-05
snap_masked-ptg000643l-processed-gene-7.150-mRNA-1	*KCNA4a*	voltage-gated potassium channel	potassium voltage-gated channel subfamily A member 4a	transmembrane ion transport	-4.05	0.000266439
snap_masked-ptg000633l-processed-gene-25.9-mRNA-1	*KCNA5b*	voltage-gated potassium channel	potassium voltage-gated channel subfamily A member 5b	transmembrane ion transport	-4.65	0.001218785
snap_masked-ptg000633l-processed-gene-26.11-mRNA-1	*KCNA6a*	voltage-gated potassium channel	potassium voltage-gated channel subfamily A member 6a	transmembrane ion transport	-5.40	2.72077E-12
maker-ptg000600l-augustus-gene-13.28-mRNA-1	*KCNA7b*	voltage-gated potassium channel	potassium voltage-gated channel subfamily A member 7b	transmembrane ion transport	-7.39	0.000445521
maker-ptg000072l-snap-gene-0.6-mRNA-1	*KCNAB2*	voltage-gated potassium channel	voltage-gated potassium channel subunit beta-2	transmembrane ion transport	-3.37	9.48977E-05

maker-ptg000068l-snap-gene-8.7-mRNA-1	*KCNB1*	voltage-gated potassium channel	potassium voltage-gated channel subfamily B member 1	transmembrane ion transport	-5.58	0.006990731
maker-ptg001248l-est_gff_est2genome-gene-0.0-mRNA-1	*KCNE4*	voltage-gated potassium channel	potassium voltage-gated channel subfamily E regulatory subunit 4	transmembrane ion transport	-1.99	0.002653689
maker-ptg000314l-augustus-gene-2.9-mRNA-1	*KCNIP4*	voltage-gated potassium channel	Kv channel-interacting protein 4	transmembrane ion transport	-3.20	0.000884639
maker-ptg001348l-snap-gene-2.39-mRNA-1	*KCNK4*	potassium two pore domain channel	potassium channel subfamily K member 4	transmembrane ion transport	-4.94	0.00032563
maker-ptg000170l-snap-gene-11.9-mRNA-1	*KCNK7*	potassium two pore domain channel	potassium channel subfamily K member 1	transmembrane ion transport	-5.11	9.18843E-12
maker-ptg000253l-snap-gene-49.55-mRNA-1	*KCNN4*	calcium-activated potassium channel	potassium calcium-activated channel subfamily N member 4	transmembrane ion transport	-5.04	1.88707E-11
maker-ptg000721l-snap-gene-3.11-mRNA-1	*SCN3b*	voltage-gated sodium channel	sodium voltage-gated channel beta subunit 3	transmembrane ion transport	-2.11	5.45393E-10
maker-ptg000974l-augustus-gene-6.41-mRNA-1	*SCN4ab*	voltage-gated sodium channel	sodium channel protein type 4 subunit alpha b	transmembrane ion transport	-5.38	4.17695E-08
maker-ptg000422l-augustus-gene-0.23-mRNA-1	*SRL*	Ca2+-binding protein in SR; Ca2+ buffering	sarcalumenin	transmembrane ion transport	-6.02	5.45506E-07
maker-ptg000562l-augustus-gene-8.257-mRNA-1	*TMEM38a*	monovalent cation channel in the SR and nuclear membranes of skeletal muscle	transmembrane protein 38A	transmembrane ion transport	-1.77	3.0994E-08
maker-ptg000600l-snap-gene-7.69-mRNA-1	*TRPM4*	Ca2 +-activated nonselective monovalent cation channel	transient receptor potential cation channel subfamily M member 4	transmembrane ion transport	-4.84	1.11494E-06
snap_masked-ptg002114l-processed-gene-2.64-mRNA-1	*ABCC9*	subunit of ATP-sensitive potassium channels	ATP-binding cassette sub-family C member 9	transmembrane ion transport	-3.10	3.68287E-09
maker-ptg000181l-snap-gene-1.66-mRNA-1	*SLC4a7*	sodium bicarbonate cotransporter	solute carrier family 4 member 7	transmembrane ion transport	-4.78	1.00112E-09
maker-ptg000361l-snap-gene-18.27-mRNA-1	*SLC22a23*	antiporter to transport organic ions across cell membranes	solute carrier family 22 member 23	transmembrane ion transport	-4.24	0.004015017
maker-ptg000718l-augustus-gene-55.59-mRNA-1	*SLC25a12*	mitochondrial electrogenic aspartate/glutamate antiporter	solute carrier family 25 member 12	transmembrane ion transport	-3.53	1.18714E-12
maker-ptg000230l-snap-gene-5.12-mRNA-1	*SLC41a1*	Na+/Mg2+ ion exchanger	solute carrier family 41 member 1	transmembrane ion transport	-4.91	5.94485E-08
maker-ptg000600l-augustus-gene-10.17-mRNA-1	*SLC6a16*	Na(+)- and Cl(-)-dependent neurotransmitter transporter	solute carrier family 6 member 16	transmembrane ion transport	-2.21	1.60618E-05
maker-ptg000299l-snap-gene-9.30-mRNA-1	*SLC6a6b*	taurine:sodium symporter	solute carrier family 6 member 6b	transmembrane ion transport	-2.58	1.66873E-11
