# Supplementary material for: Gene and Allele-Specific Expression Underlying the Electric Signal Divergence in African Weakly Electric Fish
Source: Mol Biol Evol. 2024 Feb 15;41(2):msae021. doi: 10.1093/molbev/msae021 (PMC10897887; doi:10.1093/molbev/msae021)
Supplement: msae021_Supplementary_Data [file msae021_supplementary_data.zip › Cheng-MBE-efishtranscriptomes-Supplementary Table 3 GO terms in up-regulatedgenes in EO.pdf]

Supplementary Table 3 44 Significantly enriched Gene Ontology terms with Fisher's exact test p-value < 0.01 in genes up-regulated in electric organ.

Term	GO terms	Category	Count	%	P-value	Genes	List Total	Pop Hits	Pop Total	Fold Enrichment	Bonferroni	Benjamini	FDR
GO:0016310	phosphorylation	Biological Process	61	5.407801	1.32E-04	*RET, SI:CH211-195B13.1, PFKFB2B, MOB1BA, PIK3CG, HK2, STK24B, RPS6KA3A, RPS6KA2, PIP5KL1, RPS6KA1, RPS6KB1B, AKT1, SI:CH211-243J20.2, PIM3, ERBB4B, PDGFRA, PRKCI, MAP4K3A, PRKCBB, DCLK1A, SI:DKEY-172J4.3, EPHA4A, SHPK, ACVR1BB, MAPK14A, PI4K2A, GNE, IPPK, ITPK1B, UCK2A, LTK, PRKX, TTK, CITA, PIP4K2AA, PRKCZ, PFKPA, CKMT1, PIP5K1CA, GRK6, ERBB2, TRIOA, CAMK1GB, MAPK6, CHKB, YES1, TNK2B, LIMK2, PTK2AB, HIPK2, PRKACBB, ETNK2, JAK2B, RPS6KAL, PRKCHA, CMPK, PI4KB, PTK7B, CDK14, CAMK1DA*	944	718	18397	1.66	0.201384509	0.224860832	0.224860832
GO:0018105	peptidyl-serine phosphorylation	Biological Process	16	1.41844	0.001444283	*PRKCI, SI:CH211-195B13.1, TTK, PRKX, TTBK1A, PRKCZ, HIPK2, PRKCBB, RPS6KA3A, RPS6KA2, RPS6KA1, RPS6KAL, RPS6KB1B, PRKCHA, CAMK1GB, CAMK1DA*	944	122	18397	2.56	0.914560516	0.684527812	0.684527812
GO:0098609	cell-cell adhesion	Biological Process	19	1.684397	0.001444379	*DCHS1A, ARNT2, NEO1A, IGSF9BA, ITGA2B, CTNND1, NRCAMA, HSPG2, TMEM47, CNTN1A, DLG2, PERP, ITGA8, CDH13, ELMO2, ITGAV, PKP4, FAT4, CDH17*	944	160	18397	2.31	0.91457447	0.684527812	0.684527812
GO:0001944	vasculature development	Biological Process	12	1.06383	0.003025118	*NOTCH2, YAP1, STN1, PANK2, ANGPT2B, CYP26C1, LGALS2A, AGAP2, ENPP2, ITGB8, RAB11A, MYCA*	944	82	18397	2.85	0.994238484	0.684527812	0.684527812
GO:0043123	positive regulation of I-kappaB kinase/NF-kappaB signaling	Biological Process	8	0.70922	0.003315885	*PRKCBB, CD40, TNIP2, TNFRSF19, RBCK1, S100B, LURAP1, MAP3K14A*	944	39	18397	4.00	0.996493066	0.684527812	0.684527812
GO:0001935	endothelial cell proliferation	Biological Process	5	0.443262	0.003380098	*ITGA2B, ITGB8, ITGAV, ARHGEF7B, HSPG2*	944	13	18397	7.50	0.996857294	0.684527812	0.684527812
GO:0030198	extracellular matrix organization	Biological Process	16	1.41844	0.003386468	*MMP15B, MMP17A, FBLN2, COLQ, SMOC2, COL4A2, ADAMTSL4, SI:DKEY-6N6.1, COL1A1A, ADAMTSL3, ADAMTS17, MMP28, COL2A1B, MMP19, ADAMTSL7, ADAMTS9*	944	133	18397	2.34	0.996891298	0.684527812	0.684527812
GO:0072583	clathrin-dependent endocytosis	Biological Process	6	0.531915	0.003582283	*AP2M1A, FCHO1, DNAJC6, SGIP1A, AP2A1, GPR107*	944	21	18397	5.57	0.997774987	0.684527812	0.684527812
GO:0070121	Kupffer's vesicle development	Biological Process	12	1.06383	0.003658342	*YAP1, MBD3B, SNX10A, VGLL4B, ARL6, RAB3IP, DNMT3BB.1, ENPP2, ITGAV, GPR22A, RAB11A, ATP6V1F*	944	84	18397	2.78	0.998046074	0.684527812	0.684527812
GO:0061817	endoplasmic reticulum-plasma membrane tethering	Biological Process	4	0.35461	0.004021903	*ESYT1A, ESYT2B, ESYT2A, GRAMD2AA*	944	7	18397	11.14	0.998950121	0.684527812	0.684527812
GO:0006486	protein glycosylation	Biological Process	16	1.41844	0.006246214	*GALNT12, ST6GAL1, GALNT13, GALNT16, B3GAT2, EXT1B, FUT8A, ST6GALNAC5A, MGAT1B, B3GNT7, ST3GAL4, ST8SIA5, ST8SIA6, STT3B, LARGE2, ST3GAL2*	944	142	18397	2.20	0.999976638	0.804864816	0.804864816
GO:0043409	negative regulation of MAPK cascade	Biological Process	6	0.531915	0.006588046	*DUSP4, DUSP5, SPRED2B, PPEF2A, SPRED2A, DUSP7*	944	24	18397	4.87	0.999986992	0.804864816	0.804864816
GO:0006811	ion transport	Biological Process	47	4.166667	0.006643522	*SLC24A2, GLRBB, KCNG3, SCN4BA, SCN1BA, TTYH2, PACC1, TMEM63C, ITPR1B, ATP2C1, ATP1A3A, CHRNG, ATP1B1B, ATP2A2B, KCNQ5B, SLC39A7, GABRD, ATP1A1A.4, SI:CH211-225P5.8, ATP6V1F, SLC13A1, SCN4AA, HEPHL1B, GABRA1, CHRNB4, SLC8A1A, KCNJ9, SLC11A2, SLC39A10, TRPV1, SI:DKEY-28B4.8, CNGA3A, GRIN2AA, SLC5A9, ATP6V0A1A, KCNJ2A, KCNK2A, SLCO4A1, ATP1A2A, ATP2B1A, ATP2B3B, CACNA1BA, VDAC1, SLC4A4A, SLC4A4B, MCU, GRIA3B*	944	614	18397	1.49	0.999988172	0.804864816	0.804864816
GO:0030030	cell projection organization	Biological Process	11	0.975177	0.006953193	*CATIP, SNX10A, INTU, ARL6, CFL1, IFT122, TMEM237A, GPR22A, CCDC61, GSNA, SDCCAG8*	944	79	18397	2.71	0.999993042	0.804864816	0.804864816
GO:0000188	inactivation of MAPK activity	Biological Process	5	0.443262	0.007609678	*DUSP4, DUSP5, SPRED2B, SPRED2A, DUSP7*	944	16	18397	6.09	0.999997742	0.804864816	0.804864816
GO:0048593	camera-type eye morphogenesis	Biological Process	5	0.443262	0.007609678	*SOX11A, LAMA1, LUM, ALDH1A2, IFT122*	944	16	18397	6.09	0.999997742	0.804864816	0.804864816
GO:0007264	small GTPase mediated signal transduction	Biological Process	13	1.152482	0.00803919	*RAC3A, BCAR3, ARHGAP32A, RHOUB, DOCK4B, GDI1, DOCK7, TIAM1B, RHOF, RAPGEFL1, RHOBTB1, RHOCB, RASGEF1BA*	944	106	18397	2.39	0.999998919	0.804864816	0.804864816
GO:0006790	sulfur compound metabolic process	Biological Process	4	0.35461	0.008935331	*CHST6, CHST7, CHST2B, SI:CH73-62B13.1*	944	9	18397	8.66	0.999999768	0.844885165	0.844885165
GO:0016020	membrane	Cellular Component	464	41.13475	3.42E-10	*SI:DKEY-34D22.1, PGAP2, TMEM200A, OLFCS1, EXT1B, CORO2BA, ZFYVE28, LAMB1A, NSDHL, CHST2B, TIAM1B, BCR, BCAM, SLC5A9, GRAMD1BA, CACNA1BA, MCOLN1A, PI4K2A, GRAMD1BB, FREM3, CDC42SE1, RPN2, TTYH2, SGIP1A, ABCB5, MBOAT2B, HACD3, SI:DKEY-91M11.5, PDGFC, CHST10, ANO5B, ST3GAL4, GSG1L2B, ST3GAL2, PLXNB1B, PLXNB1A, ABCC6A, PRRT1, ABCA2, CADM3, ICMT, APCDD1L, SI:DKEY-15H8.17, SI:CH211-286O17.1, EIF5, NPTNB, MCOLN3A, TMEM237A, NMT2, SPIRE1A, PAM, RALAA, RET, ACHE, MTMR10, MTMR11, TMEM181, LYST, SLC6A2, PHEX, CYB561D2, TMEM47, CATIP, ADAMTSL3, SH3GLB2B, ATP1A1A.4, TMEM119B, CHST6, CHST7, RHBDF1A, ELOVL2, SLC39A10, GNL1, PRSS12, FAM234B, ECRG4A, SLCO4A1, ACVR1BB, TSPAN7B, ERGIC3, PIGF, MGLL, PACC1, SLC16A6B, TMEM72, ADCY7, PPP1R3AA, CHRNG, GDPD4A, MUC13B, SLC17A5, SC5D, GABRA1, SNX21, PLXDC1, TSPAN9A, GDPD5B, SH3GLB1A, CYB561, RIC3B, RCA2.1, TMEM229B, GPR146, SEMA5A, ADCY1A, GLRBB, KCNG3, NRROS, TUSC3, CLSTN1, ITGA2B, CXCL14, KDELR2B, AP2M1A, ABHD12, LAPTM4B, RNF19A, CFL1, TSPAN4A, SI:CH211-1E14.1, SI:DKEY-122A22.2, PLXNC1, SLC39A7, PHLDA2, IL13RA2, LARGE2, PHLDA3, SEMA6A, SLC25A23B, BSCL2, FRMD3, TOM1L2, TRIM101, MAG, MFSD12A, SYPL1, MADD, ROR2, SI:CH73-62B13.1, GRIA3B, NOTCH2, ABHD2A, ARL6, TMEM230A, LIN7C, PFKPA, MGAT1B, ADTRP1, SI:CH211-76L23.7, SI:CH211-225P5.8, DRD4B, KL, SLC37A2, OSBPL6, RAB4B, SDF2L1, GPR22A, SLC25A25B, CYP51, TMPRSS15, GRIN2AA, CNGA3A, G6PC3, TSPAN5A, CLTCB, GPR107, MCU, SPRED2B, DYSF, NENF, MRC1A, PIK3CG, B4GALNT3A, SPRED2A, SNX10A, SI:CH73-364H19.1, NAPBB, CERS3A, ITGB8, ITGAV, IL21R.1, CCDC51, SLC13A1, ADGRV1, CERS6, ZGC:110329, CHPF2, TNFRSF19, NRG1, SLC25A55A, ST6GALNAC5A, RRBP1A, SYNGR3A, PRKCBB, ZDHHC8B, KCNJ2A, SLC15A1A, ITGA8, EFHC1, TNFRSF21, CERS1, SLC24A2, NLGN1, NEO1A, ATP10D, LRP5, JAKMIP3, AP3M2, CKMT1, FCHO1, CYB5A, LMBRD2B, KCNJ9, SEMA4C, MBOAT1, MCOLN2, SI:DKEY-*	983	7112	18868	1.25	1.30E-07	1.30E-07	1.27E-07

GO:0005886	plasma membrane	Cellular Component	237	21.01064	6.08E-08	*ZGC:165507, SI:DKEY-34D22.1, SCN1BA, CPNE7, OLFCS1, ITPR1B, RAPGEFL1, ZDHHC4, EFR3A, MFSD2B, EHD1B, ESYT2B, ESYT2A, ENTPD1, PRKCI, SLC6A17, ACTN1, SLC11A2, TIAM1B, ADGRA3, HSPG2, BCAM, SLC5A9, CCNY, PRKAR1B, ATP1A2A, EPHA4A, ATP2B1A, GRAMD1BA, MCOLN1A, VDAC1, PI4K2A, GRAMD1BB, LRP1BB, CDC42SE1, ESYT1A, TTYH2, RRAD, TMEM63C, SGIP1A, MYO6A, PCDH19, CRHR1, CD79B, ATP1B1B, ANO5B, GSG1L2B, STXBP6, ANO10B, RHOCB, PLXNB1B, PLXNB1A, CARMIL2, YES1, CARMIL3, VASNB, APCDD1L, NPTNB, MCOLN3A, AVPR1AA, BAMBIA, FAT4, SPIRE1A, RALAA, RET, ACHE, DOCK4B, TENM4, SLC6A2, PHEX, CATIP, ERBB4B, ATP1A1A.4, TMEM119B, DCHS1B, HEPHL1B, SI:CH211-264F5.2, DCHS1A, SLC39A10, TMEM30AB, ANO6, MMEL1, RHOF, GMIP, PRSS12, ZDHHC17, RGMA, ECRG4A, SLCO4A1, ACVR1BB, AVPR2AA, TSPAN7B, DSCAML1, SLC5A7A, SORBS2A, EPS15L1A, SLC16A6B, PACC1, AMIGO1, NRN1A, GUCY2F, ADCY7, SEMA3AB, ATP1A3A, CHRNG, SLC17A5, ARF3A, GABRD, RASGEF1BA, SCCPDHA.1, GABRA1, STAC3, GDPD5B, TSPAN9A, ATP2B3B, SLC6A6B, RIC3B, UNC13BB, ADCY1A, TRHDE.1, GLRBB, GRM6A, NRROS, CTNND1, ACSL4A, AP2M1A, RAB44, LAPTM4B, FAM174B, IGFLR1, TSPAN4A, PLXNC1, PHLDA3, PDGFRA, CHRNB4, SLC8A1A, LMTK2, CYBA, TTC7A, KCTD7, MAG, MFSD12A, PSTPIP1A, PKP4, ROR2, ABCG1, GRIA3B, NOTCH2, ABHD2A, SCARB2A, ARL6, PSEN2, LDLRB, PIP4K2AA, LIN7C, RHOBTB1, PIP5K1CA, GRK6, CLDN11A, CNRIP1B, ATP6V1F, MFAP3L, DRD4B, OSBPL6, RAB4B, OPN3, CAV3, STXBP1A, ACSL3B, CRIM1, TRPV1, GPR22A, GRIN2AA, CNGA3A, DLG2, CAPRIN2, TSPAN5A, FLOT2A, HSP90AB1, SPRED2B, DYSF, NRCAMA, TRH, MRC1A, PIK3CG, RERG, GRM4, SEMA3GB, PIP5KL1, ADGRV1, ZGC:110329, TNFRSF19, ATP6AP2, ATP6V0A1A, KCNJ2A, NID1A, CPTP, SLC15A1A, CDH13, CDH17, TNFRSF21, SLC24A2, RAC3A, NLGN1, LTK, NEO1A, SCN4BA, ATP10D, LRP5, PMP22B, ABHD17C, ATP2C1, CNTN1A, GNG5, FCHO1, ERBB2, SH2B2, SI:DKEY-206P8.1, SCN4AA, PDZD7A, LMBRD2B, TNK2B, MYO1EA,*	983	3299	18868	1.38	2.30E-05	1.15E-05	1.12E-05
GO:0005794	Golgi apparatus	Cellular Component	62	5.496454	2.02E-06	*GALNT12, SLC35B4, GALNT13, GALNT16, PGAP2, CLSTN1, EXT1B, B4GALNT3A, ZDHHC4, RAB44, STK24B, FAM174B, SLC39A7, LARGE2, ADAM19B, PDGFRA, ST6GAL1, RHBDF1A, CHPF2, ZDHHC13, TMEM30AB, FUT8A, ZDHHC17, ST6GALNAC5A, ZDHHC14, CSGALNACT1A, SREBF2, ZDHHC8B, B3GNT7, CPTP, ZGC:162200, ERGIC3, FAM20CB, GRINAA, PI4K2A, RNF128A, TMEM230A, MYO6A, PSEN2, RFNG, TMEM241, AP3M2, MGAT1B, CHSY1, CHST10, ST3GAL4, ST8SIA5, ST8SIA6, ZGC:162698, ST3GAL2, ARHGAP32A, CAV3, B3GAT2, YIPF5, CLASP1A, ZDHHC23B, RAB11A, PLEKHA8, ZDHHC9, NDST1B, SH3GLB1A, GPR107*	983	628	18868	1.89	7.65E-04	2.55E-04	2.49E-04
GO:0005783	endoplasmic reticulum	Cellular Component	68	6.028369	1.89E-05	*NRROS, PGAP2, EXT1B, ITPR1B, ACSL4A, TXNDC12, PITPNBL, ZDHHC4, CYB561D2, KDELR2B, ABHD12, SEC61A1, NSDHL, CERS3A, SI:DKEY-122A22.2, SMPD2B, SLC39A7, ESYT2B, ESYT2A, CERS6, RHBDF1A, ELOVL2, ATP6AP2, LMF2A, BRINP3A.1, SRD5A2A, TMEM30AB, PDIA8, ZDHHC14, SREBF2, PDIA4, BSCL2, RCN2, ERGIC3, CRELD2, NAT8L, GRINAA, RNF128A, CERS1, NECAB3, PDXDC1, RPN2, PSEN2, HSD17B3, ATP2C1, HACD3, ORMDL2, REEP3B, CALR3A, KL, SLC37A2, ICMT, SDF2L1, BNIP3, YIPF5, ACSL3B, SERPINH1B, NCK2B, ZDHHC23B, CYP51, G6PC3, TBL2, CERS2B, ZDHHC9, MGAT4A, DOLPP1, PI4KB, RIC3B*	983	763	18868	1.71	0.007155957	0.001795404	0.001752769
GO:0000139	Golgi membrane	Cellular Component	35	3.102837	1.26E-04	*GALNT12, SLC35B4, GALNT13, GALNT16, PGAP2, CLSTN1, PSEN2, RFNG, ATP2C1, ZDHHC4, KDELR2B, MAN2A2, CHST10, QSOX1, LARGE2, PCSK5B, CHST6, RHBDF1A, RIC1, CHST7, CAV3, CHST2B, B3GAT2, ZDHHC13, ZDHHC23B, ZDHHC17, ZDHHC14, SREBF2, ZDHHC8B, ZDHHC9, B3GNT7, NDST1B, SH3GLB1A, ERGIC3, SI:CH73-62B13.1*	983	331	18868	2.03	0.04670409	0.00956538	0.009338234
GO:0005789	endoplasmic reticulum membrane	Cellular Component	48	4.255319	2.51E-04	*CDS1, ESYT1A, SI:DKEY-13N15.2, DIPK1B, NRROS, SLC35B4, PGAP2, EXT1B, PSEN2, FMN2B, ITPR1B, HACD3, ZDHHC4, KDELR2B, ABHD12, SEC61A1, CERS3A, ORMDL2, REEP3B, SC5D, SLC39A7, CALR3A, ESYT2B, ESYT2A, SLC37A2, ICMT, CERS6, RHBDF1A, SDF2L1, ELOVL2, LMF2A, ATP6AP2, YIPF5, SCDB, ZDHHC14, SREBF2, RRBP1A, CYP51, BSCL2, G6PC3, ZDHHC9, CERS2B, DOLPP1, GRAMD1BA, ERGIC3, SEC24D, PIGF, GRAMD1BB*	983	529	18868	1.74	0.090736226	0.015851352	0.015474934

GO:0016021	integral component of membrane	Cellular Component	309	27.39362	0.00231972	*SLC23A2, SLC35B4, ZGC:165507, PGAP2, SI:DKEY-34D22.1, PLEKHB2, TMEM200A, EXT1B, TXNDC11, ITPR1B, FRMD4BA, ZDHHC4, NPDC1A, NSDHL, STS, SLITRK3A, ARL10, ESYT2B, ESYT2A, ENTPD1, ST6GAL1, CHST2B, SLC11A2, MMP15B, ADGRA3, BCAM, SLC5A9, ATP1A2A, EPHA4A, GRAMD1BA, MCOLN1A, GRAMD1BB, FREM3, LRP1BB, CDS1, ESYT1A, RPN2, TMEM63C, SGIP1A, ABCB5, MBOAT2B, PCDH19, HACD3, CRHR1, CD79B, CHST10, PDGFC, ANO5B, ST3GAL4, ST8SIA5, ST8SIA6, GSG1L2B, ANO10B, ST3GAL2, PRRT1, ABCC6A, ABCA2, CADM3, TMEM86A, TMEM184C, ICMT, VASNB, APCDD1L, SI:DKEY-15H8.17, NRXN3A, SI:CH211-286O17.1, FLRT1B, EIF5, GPR180, NPTNB, MCOLN3A, TMEM237A, NCAM1A, FAT4, PAM, AHCTF1, TENM4, TMEM181, PHEX, CYB561D2, ZCCHC14, TMEM47, ADAMTSL3, ATP2A2B, SMPD2B, ERBB4B, ATP1A1A.4, TMEM119B, SI:CH211-264F5.2, DCHS1A, CHST6, RHBDF1A, ZDHHC13, SLC39A10, TMEM30AB, ANO6, MMEL1, GNL1, ZDHHC17, CSGALNACT1A, ZDHHC14, SREBF2, RGMA, FAM234B, B3GNT7, SLCO4A1, CRELD1, ERGIC3, GRINAA, DSCAML1, PIGF, SLC5A7A, RPRMA, ZGC:92045, SLC16A6B, PACC1, HSD17B3, TMEM72, GUCY2F, PPP1R3AA, TMEM164, MEGF6A, ATP1A3A, GDPD4A, ORMDL2, PERP, MEP1A.1, MUC13B, REEP3B, SLC17A5, KCNQ5B, SC5D, ZGC:162698, ZGC:92275, YIPF5, KLHL2, ZDHHC23B, PLXDC1, DPY19L3, ZDHHC9, GDPD5B, CYB561, RCA2.1, RIC3B, TMEM229B, GPR146, GALNT12, SEMA5A, TRHDE.1, KCNG3, GALNT13, NRROS, GALNT16, TUSC3, CLSTN1, ITGA2B, ACSL4A, COX6A1, TMEM263, KDELR2B, ABHD12, LAPTM4B, TMEM268, FAM174B, RNF19A, IGFLR1, SI:CH211-1E14.1, SI:DKEY-122A22.2, SLC39A7, IL13RA2, LARGE2, ADAM19B, PDGFRA, SLC8A1A, SEMA6A, CCR12A, IGSF9BA, APLP2, SRD5A2A, SHISA3, ACYP1, SLC25A23B, FUT8A, FRMD3, TOM1L2, ALDH3A1, TRIM101, MAG, B3GLCTA, SYPL1, TMCC1B, FAM20CB, ABCG1, AQP1A.1, NOTCH2, DDX5, ABHD2A, DIPK1B, SCARB2A, TMEM230A, PSEN2, SI:CH211-241B2.5, LDLRB, TMEM241, TMEM242, ADTRP1, MGAT1B,*	983	5161	18868	1.15		0.585297993	0.125596253	0.122613757
GO:0005905	clathrin-coated pit	Cellular Component	7	0.620567	0.003965195	*AP2M1A, HIP1RB, FCHO1, SGIP1A, MYO6A, CLTCB, AP2A1*	983	30	18868	4.48		0.778159411	0.187851107	0.183390263
GO:0031012	extracellular matrix	Cellular Component	22	1.950355	0.004951409	*NRROS, VASNB, MMP15B, MMP17A, TSKU, COLQ, VCANB, CCN4A, COL4A2, ADAMTSL4, SI:DKEY-6N6.1, COL1A1A, ADAMTSL3, ADAMTS17, SI:CH211-106H11.3, MMP28, COL2A1B, TIMP2A, MMP19, SI:DKEY-65B12.6, ADAMTSL7, ADAMTS9*	983	218	18868	1.94		0.84759982	0.208509355	0.203557945
GO:0030424	axon	Cellular Component	19	1.684397	0.005735502	*RET, SCN4AA, SYNM, SLC8A1A, SCN1BA, CLSTN1, ATP6AP2, ROGDI, ELAVL4, CCKA, NRCAMA, RAB11A, SI:DKEY-91M11.5, BCR, CNTN1A, DLG2, NPTNB, DSCAML1, SLC5A7A*	983	179	18868	2.04		0.886959935	0.217375523	0.212213572
GO:0042995	cell projection	Cellular Component	31	2.748227	0.007956686	*RAC3A, TENM4, INTU, ARL6, CLSTN1, ROGDI, ELAVL4, MYO6A, ABHD17C, RHOBTB1, SI:DKEY-91M11.5, LAPTM4B, TEKT3, RHOCB, ACTR3, PDGFRA, ADGRV1, ACTN1, IFT122, PTK2AB, RHOF, ZDHHC17, BCR, DLG2, TMEM218, TMEM237A, PI4K2A, GSNA, CCDC61, MACF1A, SDCCAG8*	983	361	18868	1.65		0.951570454	0.252320114	0.246328344
GO:0031410	cytoplasmic vesicle	Cellular Component	24	2.12766	0.008805908	*RAC3A, SI:DKEY-13N15.2, DENND4C, ROGDI, SPRED2B, MYO6A, DYSF, ZDHHC13, RHOF, ZDHHC17, SREBF2, RHOBTB1, KDELR2B, AP3M2, CADPSA, AP2M1A, TBC1D7, CLTCB, SPIRE1A, SEC24D, FLOT2A, RHOCB, CYB561, PI4K2A*	983	259	18868	1.78		0.964993461	0.252320114	0.246328344
GO:0031234	extrinsic component of cytoplasmic side of plasma membrane	Cellular Component	8	0.70922	0.009171801	*ESYT1A, ESYT2B, ESYT2A, YES1, TNK2B, STAC3, PTK2AB, GRAMD2AA*	983	46	18868	3.34		0.969564691	0.252320114	0.246328344
GO:0031227	intrinsic component of endoplasmic reticulum membrane	Cellular Component	4	0.35461	0.009320532	*ESYT1A, ESYT2B, ESYT2A, GRAMD2AA*	983	9	18868	8.53		0.97124797	0.252320114	0.246328344
GO:0016740	transferase activity	Molecular Function	126	11.17021	3.52E-05	*GALNT12, GALNT13, PRDM9, GALNT16, PFKFB2B, GTF2B, EXT1B, ZDHHC4, RPS6KA3A, RPS6KA2, RNF19A, RPS6KA1, AKT1, PIM3, LARGE2, PDGFRA, PRKCI, ST6GAL1, CHST2B, UBE2E3, MAP4K3A, FUT8A, RC3H2, DCLK1A, SI:CH211-256M1.8, B3GLCTA, EPHA4A, MAPK14A, RBCK1, PI4K2A, SI:CH73-62B13.1, GNE, CDS1, IPPK, UCK2A, ITPK1B, CRATA, UAP1, CITA, PIP4K2AA, PRKCZ, PFKPA, MGAT1B, PIP5K1CA, GRK6, CHST10, ST3GAL4, ST8SIA5, ST8SIA6, TRIOA, CAMK1GB, ST3GAL2, YES1, ICMT, CHKB, JAK2B, PRKCHA, NMT2, PI4KB, STT3B, PTK7B, CDK14, RET, PYGB, SI:CH211-195B13.1, MOB1BA, PIK3CG, HK2, B4GALNT3A, GYS1, STK24B, CPT2, CERS3A, PIP5KL1, RPS6KB1B, SI:CH211-243J20.2, ERBB4B, CHST6, CHST7, CERS6, CHPF2, ELOVL2, ZDHHC17, ZDHHC14, CSGALNACT1A, ST6GALNAC5A, APRT, PRKCBB, ZDHHC8B, KAT2A, MYLIPA, B3GNT7, UBE2R2, SI:DKEY-172J4.3, ACVR1BB, NAT8L, RNF213A, KMT2CA, LTK, UHRF1, PRKX, TTK, RFNG, METTL21A, CKMT1, CHSY1, ERBB2, MAPK6, RNF20, EHMT1B, TNK2B, B3GAT2, LIMK2, DNMT3BB.1, PTK2AB, ZDHHC23B, HIPK2, CERS2B, PRKACBB, ZDHHC9, ETNK2, BMT2, NDST1B, RPS6KAL, CMPK, CAMK1DA*	880	1743	17340	1.42		0.027705485	0.014324224	0.014288368

GO:0005509	calcium ion binding	Molecular Function	64	5.673759	3.59E-05	*RET, SNED1, CAPN1A, CAPN1B, FKBP14, CETN2, PDCD6, CLSTN1, CETN3, DYSF, ANXA11B, ITPR1B, EFHD2, ENPP2, EFHD1, EHD1B, DCHS1B, ESYT2B, ESYT2A, DCHS1A, EGFL6, ACTN1, ANXA4, VWDE, SLC25A23B, HSPG2, MYL4, VCANB, RCN2, NID1A, CDH13, CRELD1, PPEF2A, CRELD2, CDH17, GSNA, LRP1BB, FBLN7, ESYT1A, NOTCH2, EPS15L1A, DIPK1B, NECAB3, SWAP70B, LDLRB, PCDH19, FBLN2, MEGF6A, SPOCK3, REPS2, SLIT2, CALR3A, S100B, SLC25A25B, EDIL3A, MACF1B, SMOC2, PVALB9, FAT4, PCDH1G31, KCNIP3A, UNC13BB, MACF1A, CRACR2AA*	880	739	17340	1.71	0.028242471	0.014324224	0.014288368
GO:0016301	kinase activity	Molecular Function	60	5.319149	1.72E-04	*RET, SI:CH211-195B13.1, PFKFB2B, MOB1BA, PIK3CG, HK2, STK24B, RPS6KA3A, RPS6KA2, PIP5KL1, RPS6KA1, RPS6KB1B, AKT1, SI:CH211-243J20.2, PIM3, ERBB4B, PDGFRA, PRKCI, MAP4K3A, PRKCBB, DCLK1A, SI:DKEY-172J4.3, EPHA4A, ACVR1BB, MAPK14A, PI4K2A, GNE, IPPK, ITPK1B, UCK2A, LTK, PRKX, TTK, CITA, PIP4K2AA, PRKCZ, PFKPA, CKMT1, PIP5K1CA, GRK6, ERBB2, TRIOA, CAMK1GB, MAPK6, CHKB, YES1, TNK2B, LIMK2, PTK2AB, HIPK2, PRKACBB, ETNK2, JAK2B, RPS6KAL, PRKCHA, CMPK, PI4KB, PTK7B, CDK14, CAMK1DA*	880	718	17340	1.65	0.128144638	0.032432442	0.03235126
GO:0035091	phosphatidylinositol binding	Molecular Function	16	1.41844	1.87E-04	*ESYT1A, ESYT2B, ESYT2A, ARHGAP32A, SI:CH211-195B13.1, ITPR1B, PITPNBL, SNX21, STAM, ZCCHC14, TOM1L2, SH3YL1, SNX10A, HIP1RB, SNX7*	880	102	17340	3.09	0.138769321	0.032432442	0.03235126
GO:0004711	ribosomal protein S6 kinase activity	Molecular Function	5	0.443262	2.03E-04	*RPS6KA3A, RPS6KA2, RPS6KA1, RPS6KAL, RPS6KB1B*	880	7	17340	14.07	0.149710731	0.032432442	0.03235126
GO:0005388	calcium-transporting ATPase activity	Molecular Function	5	0.443262	0.003245591	*SI:DKEY-28B4.8, ATP2B1A, ATP2A2B, ATP2B3B, ATP2C1*	880	13	17340	7.58	0.925536363	0.401975219	0.400969023
GO:0004222	metalloendopeptidase activity	Molecular Function	15	1.329787	0.003521685	*MMP15B, MMEL1, PAPPAA, MMP17A, PHEX, PITRM1, ADAMTSL4, ADAMTSL3, ADAMTS17, MEP1A.1, MMP28, MMP19, ADAMTSL7, ADAMTS9, ADAM19B*	880	122	17340	2.42	0.940322064	0.401975219	0.400969023
GO:0000166	nucleotide binding	Molecular Function	111	9.840426	0.004302395	*ADCY1A, PANK2, TAOK2A, NUBP2, RPS6KA3A, RPS6KA2, RPS6KA1, DHX58, AKT1, PIM3, EHD1B, PDGFRA, PRKCI, ENTPD1, UBE2E3, MAP4K3A, AARSD1, DCLK1A, SI:CH211-256M1.8, PRKAR1B, EPHA4A, ATP1A2A, ATP2B1A, MAPK14A, ROR2, PI4K2A, ABCG1, IPPK, UCK2A, ITPK1B, DDX5, RTEL1, DHX8, ARL6, RRAD, ABCB5, MYO6A, TUBA8L3, TUBA8L2, CITA, PIP4K2AA, PRKCZ, PFKPA, PIP5K1CA, GRK6, TRIOA, CAMK1GB, MYH10, RHOCB, SI:DKEY-32E23.4, ABCC6A, ABCA2, RAB4B, YES1, MYO15AA, EIF5, JAK2B, PRKCHA, PI4KB, CDK14, RALAA, KIF13BA, RET, SI:CH211-195B13.1, HSP90AB1, MCM7, HK2, KIF15, ARL5C, PIP5KL1, ATP2A2B, RPS6KB1B, SI:CH211-243J20.2, ERBB4B, ATP1A1A.4, MBD3B, GNL1, PRKCBB, SI:DKEY-28B4.8, UBE2R2, SI:DKEY-172J4.3, ACVR1BB, EEF1A1B, BLVRA, LTK, ATP10D, PRKX, TTK, ATP2C1, ADCY7, CKMT1, ATP1A3A, RASD1, SI:CH211-257P13.3, ERBB2, ARF3A, MAPK6, KIF26BA, MAP4K4, TNK2B, MYO1EA, PTK2AB, HIPK2, PRKACBB, ATP2B3B, RPS6KAL, ABCG2A, CMPK, KRAS, CAMK1DA, RAN*	880	1703	17340	1.28	0.968096199	0.429701734	0.428626135
GO:0001517	N-acetylglucosamine 6-O-sulfotransferase activity	Molecular Function	4	0.35461	0.006000047	*CHST6, CHST7, CHST2B, SI:CH73-62B13.1*	880	8	17340	9.85	0.991839725	0.510765439	0.509486928
GO:0005085	guanyl-nucleotide exchange factor activity	Molecular Function	21	1.861702	0.006592485	*BCAR3, ARHGEF10, RIC1, DOCK4B, IQSEC3A, DENND4C, RAB3IP, DOCK7, TIAM1B, RAB3IL1, ARHGEF7A, ARHGEF9B, ARHGEF7B, RAPGEFL1, SI:DKEY-91M11.5, NET1, BCR, MADD, TRIOA, SBF1, RASGEF1BA*	880	215	17340	1.92	0.994932132	0.510765439	0.509486928
GO:0051015	actin filament binding	Molecular Function	23	2.039007	0.007522058	*ACTR3, MBD3B, MYO1EA, FHOD1, ACTN1, MYO6A, MYO15AA, NEB, SHROOM1, ARPC5LA, CORO2BA, DUB, PSTPIP1A, SAMD14, HIP1RB, ARPC3, CFL2, CFL1, TWF1B, GAS2L1, CTNNAL1, MYH10, GSNA*	880	247	17340	1.83	0.997601334	0.510765439	0.509486928
GO:0016409	palmitoyltransferase activity	Molecular Function	7	0.620567	0.00767107	*ZDHHC8B, ZDHHC9, ZDHHC13, ZDHHC23B, ZDHHC17, ZDHHC14, ZDHHC4*	880	35	17340	3.94	0.997872515	0.510765439	0.509486928
