# Supplementary material for: Gene and Allele-Specific Expression Underlying the Electric Signal Divergence in African Weakly Electric Fish
Source: Mol Biol Evol. 2024 Feb 15;41(2):msae021. doi: 10.1093/molbev/msae021 (PMC10897887; doi:10.1093/molbev/msae021)
Supplement: msae021_Supplementary_Data [file msae021_supplementary_data.zip › Cheng-MBE-efishtranscriptomes-Supplementary Table 4 GO terms indown-regulated genes in EO.pdf]

Supplementary Table 4 76 Significantly enriched Gene Ontology terms with Fisher's exact test p-value < 0.01 in genes down-regulated in electric organ.

Term	GO terms	Category	Count	%	P-value	Genes	List Total	Pop Hits	Pop Total	Fold Enrichment	Bonferroni	Benjamini	FDR
GO:0048741	skeletal muscle fiber development	Biological Process	22	2.263374	1.52E-13	*CAVIN4B, KLHL41A, MYBPC1, PYGMA, RBFOX2, SMPX, MYO18AB, RBFOX1L, LGALS2A, MYO18AA, SIX1B, RYR3, ACTN2B, LMOD3, RYR1B, RYR1A, NFIXA, KLHL40B, KLHL40A, KLHL41B, SMYD1B, MYF5*	820	63	18397	7.834572203	2.09E-10	2.09E-10	2.06E-10
GO:0006936	muscle contraction	Biological Process	20	2.057613	3.14E-12	*TMOD1, TNNI1C, MYHB, TNNI4A, TMOD4, TPM3, TPM1, TNNT3B, TNNT2A, MYOM2A, MYOM1A, MYOM1B, LMOD3, LMOD2B, SPEGB, TNNI2A.4, TNNT2E, TPMA, DESMA, TNNI2A.1*	820	58	18397	7.736333053	4.33E-09	2.16E-09	2.13E-09
GO:0030239	myofibril assembly	Biological Process	16	1.646091	6.69E-11	*TMOD1, TMOD4, TNNT3B, ACTN2B, LMOD3, LMOD2B, TTN.2, TNNI2A.4, TTN.1, MEF2AA, MEF2AB, PROX1A, CRYABA, DESMA, PGM5, SMYD1B*	820	40	18397	8.974146341	9.22E-08	3.07E-08	3.02E-08
GO:0007519	skeletal muscle tissue development	Biological Process	18	1.851852	2.25E-09	*MYOG, POPDC3, CDKN1A, DNAJB6A, MYLPFB, FHL1A, STAC3, FXR1, SYNPO2LA, SYNPO2LB, TTN.2, BAG3, TTN.1, NFIXA, CRYABA, DESMA, ITGA7, MYF5*	820	65	18397	6.212870544	3.10E-06	7.75E-07	7.61E-07
GO:0045214	sarcomere organization	Biological Process	15	1.54321	6.83E-09	*KLHL41A, CAPN3A, LRRC39, TFPI2, TNNT3B, TNNT2A, ACTN2B, SMYHC2, TTN.2, TNNT2E, MYH7L, DESMA, FLNCB, KLHL41B, SMYD1B*	820	46	18397	7.31588017	9.42E-06	1.88E-06	1.85E-06
GO:0003009	skeletal muscle contraction	Biological Process	10	1.028807	4.70E-07	*TNNI1C, RYR1B, TNNI4A, ZMP:0000000930, TNNI2A.4, TNNC1B, RYR1A, STAC3, TNNI2A.1, TCAP*	820	24	18397	9.348069106	6.48E-04	1.08E-04	1.06E-04
GO:0060048	cardiac muscle contraction	Biological Process	11	1.131687	1.05E-06	*SMYHC2, TNNI1C, MYL13, TNNI4A, ZMP:0000000930, TNNI2A.4, TNNC1B, MYH7L, TNNI2A.1, TNNT2A, TCAP*	820	33	18397	7.478455285	0.001451569	2.08E-04	2.04E-04
GO:0006096	glycolytic process	Biological Process	11	1.131687	5.68E-06	*PFKMB, GPIB, INSRA, PGAM2, PKMA, TPI1B, ALDOAA, ENO3, ALDOAB, GAPDH, ALDOCB*	820	39	18397	6.327923702	0.007795445	9.78E-04	9.61E-04
GO:0014866	skeletal myofibril assembly	Biological Process	7	0.720165	4.17E-05	*MYO18AB, TPM3, MYO18AA, TMOD4, TTN.1, DUSP27, SMYD1B*	820	16	18397	9.815472561	0.055863454	0.006387031	0.006271
GO:0030036	actin cytoskeleton organization	Biological Process	19	1.954733	7.32E-05	*PDLIM5B, PDLIM3B, PHACTR3B, ACTN3B, EHBP1L1A, EHBP1L1B, ACTN2B, SSH2A, ROCK2A, CAPZB, STARD13B, DAAM2, CAPZA1B, LDB3B, XIRP1, FLNA, CORO1CA, ZGC:162952, SMTNL1*	820	144	18397	2.960221883	0.096053207	0.010098108	0.009915
GO:0016310	phosphorylation	Biological Process	55	5.658436	1.18E-04	*COQ8AA, MYLK2, DYRK4, CDKN1A, PRKAB1A, MAST2, CKMT2A, AKAP8L, CAMK2N1A, ROCK2A, EEF2K, ULK1B, MYLK4A, PLAUA, PIK3R3B, ADKB, GRK7A, PRKG1B, ADKA, ZGC:172076, HUNK, BMPR1AA, MAPKAPK3, PIK3CA, PRKCQ, MET, UCKL1B, VEGFAA, DAPK2A, RAF1A, AK1, CITA, PAK1, INSRA, ERBB2, ABL1, CDKN1CA, MAP2K6, SRPK3, PDK2A, CKMB, PFKMB, CKMA, NEK6, SI:CH211-220I18.4, NEK7, CAMK2B1, PKMA, ALPK3A, PTK2AA, AKT3A, SI:DKEY-8E10.3, MAPKAPK2A, PFKFB4B, SI:DKEY-96F10.1*	820	718	18397	1.718586521	0.149904153	0.014763328	0.014496
GO:0006099	tricarboxylic acid cycle	Biological Process	8	0.823045	2.80E-04	*CS, FH, SUCLA2, MDH2, IDH2, DLST, ACO2, IDH3A*	820	30	18397	5.982764228	0.320253974	0.032165168	0.031582
GO:0048769	sarcomerogenesis	Biological Process	5	0.514403	4.11E-04	*ZMP:0000000930, TTN.2, TTN.1, TCAP, SMYD1B*	820	9	18397	12.46409214	0.432339201	0.040436785	0.039704
GO:0035914	skeletal muscle cell differentiation	Biological Process	5	0.514403	4.11E-04	*MYOG, CDKN1A, FHL1A, KLHL41B, MYF5*	820	9	18397	12.46409214	0.432339201	0.040436785	0.039704
GO:0055001	muscle cell development	Biological Process	9	0.925926	5.41E-04	*FXR1, CAPZB, MYH7BA, NRAP, TCAP, ACTN3B, NEB, TGFBI, ACTN2B*	820	43	18397	4.695774248	0.525580303	0.049697423	0.048796
GO:0045727	positive regulation of translation	Biological Process	6	0.617284	6.83E-04	*FXR1, FXR2, PCIF1, METTL5, LARP4B, LARP1B*	820	17	18397	7.918364419	0.61028456	0.058876034	0.057809
GO:0006874	cellular calcium ion homeostasis	Biological Process	10	1.028807	0.001264	*ATP2A2A, RYR1B, RYR1A, ATP2B3B, HOMER1B, TNNT2A, ATP2A1, ATP2B2, RYR3, DHRS7CB*	820	60	18397	3.739227642	0.825237001	0.102542457	0.100683
GO:0061061	muscle structure development	Biological Process	6	0.617284	0.001532	*PDLIM5B, PDLIM3B, LDB3B, HOMER1B, KLHL40B, KLHL40A*	820	20	18397	6.730609756	0.879206058	0.115330509	0.11324
GO:0033693	neurofilament bundle assembly	Biological Process	4	0.411523	0.001589	*SYNM, SI:DKEY-33C12.3, NEFMA, NEFLA*	820	6	18397	14.95691057	0.888421012	0.115330509	0.11324
GO:0006470	protein dephosphorylation	Biological Process	16	1.646091	0.001923	*EPM2A, SI:CH211-223P8.8, PTPN4A, PTPN21, DUSP27, DUSP16, CDC25B, SSH2A, PDP1, PTPRNA, DUSP10, SI:CH211-121A2.2, DUSP22A, SI:CH211-195B15.8, DUSP13A, DUSP22B*	820	144	18397	2.492818428	0.929674738	0.132603536	0.1302
GO:0016311	dephosphorylation	Biological Process	15	1.54321	0.002433	*EPM2A, SI:CH211-223P8.8, PTPN4A, PTPN21, DUSP27, DUSP16, PTP4A3A, SSH2A, PTPRNA, DUSP10, SI:CH211-121A2.2, DUSP22A, SI:CH211-195B15.8, DUSP13A, DUSP22B*	820	133	18397	2.53030442	0.965225892	0.153840334	0.151051
GO:0016567	protein ubiquitination	Biological Process	32	3.292181	0.002548	*VCP, ANAPC16, KLHL15, ASB5B, PDZRN3B, UBR3, NEDD4L, KLHL13, ASB18, SH3RF1, ASB16, TRIM35-31, CAND2, HERC2, ASB15B, ASB10, DCAF12, CUL3B, SI:CH73-54F23.4, SOCS3B, TRIM55B, FEM1A, KLHL21, ZBTB16A, FBXO32, UBAC1, FBXO31, SI:CH211-120G10.1, ASB2A.1, NEURL2, ASB4, TRIM54*	820	406	18397	1.768304698	0.970353175	0.153840334	0.151051
GO:0007623	circadian rhythm	Biological Process	7	0.720165	0.002566	*NFIL3-6, NR0B2A, PER1B, CLOCKA, MITFA, NPAS2, ARNTL2*	820	32	18397	4.90773628	0.971069964	0.153840334	0.151051
GO:0060047	heart contraction	Biological Process	11	1.131687	0.002841	*BAG3, TTN.2, LRRC39, CRYABA, DESMA, DLST, TNNT2A, TCAP, FBXO32, SMYD1B, LIMS1*	820	80	18397	3.084862805	0.980211305	0.161969	0.159033
GO:0017148	negative regulation of translation	Biological Process	7	0.720165	0.00302	*FXR1, PAIP2B, FXR2, EIF4EBP1, CAPRIN1A, EIF4EBP3L, YBX1*	820	33	18397	4.759016999	0.984558527	0.161969	0.159033
GO:0007015	actin filament organization	Biological Process	16	1.646091	0.003054	*TMOD1, MYO5AA, TMOD4, TPM3, TPM1, RHOBTB4, MYO16, LMOD3, TMSB, LMOD2B, TPMA, XIRP1, CORO6, BCL2L16, RHOAC, CORO1CA*	820	151	18397	2.377257309	0.985266585	0.161969	0.159033
GO:0032922	circadian regulation of gene expression	Biological Process	8	0.823045	0.003497	*NFIL3-6, BHLHE40, KDM8, CRY2, PER1B, NR1D1, CLOCKA, NPAS2*	820	45	18397	3.988509485	0.992015785	0.178433114	0.175198

GO:0043409	negative regulation of MAPK cascade	Biological Process	6	0.617284	0.003623	*DUSP10, SI:CH211-223P8.8, SI:CH211-121A2.2, SI:CH211-195B15.8, DUSP13A, DUSP16*	820	24	18397	5.608841463	0.993296997	0.178433114	0.175198
GO:0046314	phosphocreatine biosynthetic process	Biological Process	4	0.411523	0.00416	*CKMB, CKMA, CKMT2A, ZGC:172076*	820	8	18397	11.21768293	0.99681468	0.185072082	0.181717
GO:0045947	negative regulation of translational initiation	Biological Process	4	0.411523	0.00416	*PAIP2B, EIF4EBP1, EIF4EBP3L, YBX1*	820	8	18397	11.21768293	0.99681468	0.185072082	0.181717
GO:2001243	negative regulation of intrinsic apoptotic signaling pathway	Biological Process	4	0.411523	0.00416	*MCL1A, MCL1B, BCL2L16, BCL2L1*	820	8	18397	11.21768293	0.99681468	0.185072082	0.181717
GO:0030388	fructose 1,6-bisphosphate metabolic process	Biological Process	5	0.514403	0.004621	*PFKMB, ALDOAA, ALDOAB, FBP2, ALDOCB*	820	16	18397	7.011051829	0.998315766	0.193081178	0.189581
GO:0006937	regulation of muscle contraction	Biological Process	5	0.514403	0.004621	*TNNT2E, TNNC1B, TNNT3B, TNNT2A, ATP2A1*	820	16	18397	7.011051829	0.998315766	0.193081178	0.189581
GO:0070588	calcium ion transmembrane transport	Biological Process	12	1.234568	0.005678	*ATP2A2A, RYR1B, CACNA1SA, RYR1A, CACNA2D2B, ATP2B3B, TRPM4A, ITPR3, ATP2A1, ATP2B2, CACNG1B, RYR3*	820	102	18397	2.639454806	0.999611057	0.230287196	0.226112
GO:0051694	pointed-end actin filament capping	Biological Process	4	0.411523	0.006035	*TMOD1, LMOD2B, TMOD4, LMOD3*	820	9	18397	9.971273713	0.999763133	0.231188731	0.226997
GO:0055008	cardiac muscle tissue morphogenesis	Biological Process	4	0.411523	0.006035	*ZMP:0000000930, LRRC39, TCAP, FBXO32*	820	9	18397	9.971273713	0.999763133	0.231188731	0.226997
GO:0055013	cardiac muscle cell development	Biological Process	5	0.514403	0.007238	*RBFOX2, ZMP:0000000930, RBFOX1L, TCAP, NR2F2*	820	18	18397	6.23204607	0.999955381	0.269758239	0.264868
GO:0030240	skeletal muscle thin filament assembly	Biological Process	4	0.411523	0.008339	*ZMP:0000000930, TCAP, LMOD3, SMYD1B*	820	10	18397	8.974146341	0.999990343	0.294860619	0.289515
GO:0006108	malate metabolic process	Biological Process	4	0.411523	0.008339	*FH, MDH2, ME1, ME3*	820	10	18397	8.974146341	0.999990343	0.294860619	0.289515
GO:0030018	Z disc	Cellular Component	26	2.674897	4.63E-17	*FHL1A, ACTN3B, RYR3, SYNPO2LA, SYNPO2LB, ZMP:0000000930, BAG3, MYOZ2A, MYOZ2B, NRAP, DESMA, CASQ1B, PDLIM5B, PDLIM3B, NEB, PARVB, ACTN2B, RYR1B, MYOZ3A, RYR1A, LDB3B, MYOZ1A, MYOZ1B, TCAP, TRIM54, LIMS1*	827	69	18868	8.596954244	1.43E-14	1.43E-14	1.37E-14
GO:0016529	sarcoplasmic reticulum	Cellular Component	15	1.54321	6.45E-12	*KLHL41A, CASQ1B, ITPR3, JPH1A, ATP2A1, JPH1B, RYR3, TRDN, ATP2A2A, RYR1B, JPH2, RYR1A, TMEM38A, KLHL41B, THBS4B*	827	30	18868	11.40749698	1.99E-09	6.78E-10	6.52E-10
GO:0005737	cytoplasm	Cellular Component	278	28.60082	6.61E-12	*APOBEC2B, UGP2B, LRRC14B, PRKAB1A, AGLA, CALCOCO1A, DCAF6, ZFYVE28, NR0B2A, ROCK2A, HERC2, EIF2D, DUSP13A, CHAC1, KLHL41B, SMU1A, JMJD4, CAVIN4B, KLHL41A, RFX2, ARMC8, RNF123, RUFY3, ULK2, PRKCQ, TTLL12, HPRT1, SI:CH211-195B15.8, KLHL40B, KLHL40A, CCNO, UCKL1B, ILRUN, NUMBL, ANAPC16, CAPN3A, DAPK2A, SGIP1A, PTPN4A, NEDD4L, NMD3, APBB2B, TRIM35-31, FXR1, LDHA, FXR2, PCBP4, CMYA5, MYL10, NAA50, SRPK3, MYO5AA, CAMK2B1, PARVB, UBAC1, ALS2B, DAZL, MAPKAPK2A, MYH7L, DUSP22A, ABLIM1A, DUSP22B, FARSB, SYNM, BTG2, DNAJB6A, SVILA, SETD3, MYLPFB, ACY1, LRRC39, RPLP0, PDZRN3B, STON2, SH3RF1, SMG6, SSH2A, MSI2B, GYS1, UCHL1, KIF1B, ZGC:85777, SI:CH73-54F23.4, MYL12.2, PLEKHO1B, CNOT6L, RBFOX1L, MYO18AB, ARG1, MYO18AA, PTGR2, TMSB, EIF4EBP3L, CLOCKA, CACTIN, ACSBG2, KANK2, FH, SIX1B, VCLB, ARNTL2, PAK1, TTN.2, FRZB, DESMA, MAP3K3, SI:CH211-260E23.9, MDH2, SI:CH211-220I18.4, STAC3, BBOX1, PKMA, SMCR8A, TUBB4B, SMYHC2, RNF146, SI:CH211-120G10.1, IMPDH1A, MSRB3, TACC2, TPI1B, PCMT, LIMS1, FHOD1, PRUNE, CTNND1, UBE3A, IPO4, PAIP2B, GRB14, ULK1B, MID1IP1L, ARHGDIA, KCNAB2B, KPNA4, BTBD10A, KPNA3, EGLN1A, EGLN1B, FGF11A, EPM2A, TXNIPA, USP4, NEFMA, VASH2, HOMER1B, ZBTB16A, CDC25B, CLIP3, CRTC1B, SI:CH211-253B8.5, ALDH3A2B, MAPKAPK3, TPMA, CRYABA, KPNB3, KANK4, IDI1, VCP, SI:DKEY-33C12.3, AK1, GSTT2, UBR3, TUBA8L2, CITA, NDRG2, NPAS2, PTP4A3A, EIF3EA, NEFLA, TOB1A, BAG3, DTNBP1A, EIF4EBP1, MTUS1A, PACSIN3, MYH14, GULP1A, ZC3H15, FGF21, MAP2K6, CCDC135, KLHL21, PER1B, YWHAG2, DAO.2, YWHAG1, FBXO32, KY, COPS3, IBTK, DNMBP, RNF150A, MYH7BA, CAPRIN1A, TWF2B, SERGEF, DYRK4, MYHB, DAGLA, SI:CH211-223P8.8, DOCK3, HSPB8, HSPB7,*	827	4405	18868	1.439856599	2.03E-09	6.78E-10	6.52E-10
GO:0031430	M band	Cellular Component	8	0.823045	1.96E-07	*SMPX, SPEGB, LRRC39, MYOM2A, MYOM1A, MYOM1B, LMOD3, SMYD1B*	827	12	18868	15.20999597	6.05E-05	1.37E-05	1.32E-05
GO:0033017	sarcoplasmic reticulum membrane	Cellular Component	9	0.925926	2.23E-07	*ATP2A2A, RYR1B, KLHL41A, RYR1A, ATP2A1, TMEM38A, KLHL41B, RYR3, TRDN*	827	17	18868	12.07852621	6.87E-05	1.37E-05	1.32E-05
GO:0016460	myosin II complex	Cellular Component	10	1.028807	1.43E-05	*SMYHC2, MYL12.2, MYHB, MYO18AB, MYL13, MYO18AA, MYH7BA, MYH7L, MYH14, MYLZ3*	827	35	18868	6.518569701	0.00439237	7.34E-04	7.05E-04

GO:0005829	cytosol	Cellular Component	77	7.921811	2.97E-05	*IPO11, UBE3A, MTR, IPO7, DUSP16, LARP1B, ZFYVE28, PSME4B, PSME4A, MAP1LC3A, MID1IP1L, ARHGDIA, EEF2L2, PGM5, CHAC1, ACY3.1, ZGC:136908, PGM1, SI:DKEY-51E6.1, USP9, ADSL, ADKB, ARG1, PDE4D, ADKA, ACOT12, AMPD1, RBP7B, LARP4B, ALDOAA, ALDOAB, IRS2B, HPRT1, SI:CH211-195B15.8, GAPDH, ASPA, FBP2, ZGC:64002, USP13, VCP, FH, AHCY, ANAPC16, RAF1A, AK1, STRIP2, BAG3, G3BP1, CDAB, AAMP, ZC3H15, NAA50, USP24, GPD1B, RIC1, GPIB, SI:CH211-260E23.9, OSBPL5, PLEKHA5, STAC3, RAD23AA, MTHFR, USP28, ALDOCB, MLLT11, RNF146, ACOT11A, AGBL1, CASTOR2, PPP2R2BB, PFKFB4B, DUSP22A, SI:DKEY-96F10.1, ACO2, TPI1B, LRCH3, DUSP22B*	827	1082	18868	1.623617869	0.009106642	0.001306889	0.001256
GO:0005861	troponin complex	Cellular Component	8	0.823045	9.52E-05	*TNNI1C, TNNI4A, TNNI2A.4, TNNT2E, TNNC1B, TNNI2A.1, TNNT3B, TNNT2A*	827	26	18868	7.01999814	0.028883725	0.003663459	0.003521
GO:0042383	sarcolemma	Cellular Component	10	1.028807	1.72E-04	*POPDC3, RYR1B, SGCB, KCNB1, RYR1A, STAC3, DESMA, PGM5, VCLB, RYR3*	827	47	18868	4.854254033	0.051663583	0.005893489	0.005664
GO:0015629	actin cytoskeleton	Cellular Component	16	1.646091	1.98E-04	*SVILA, MYO5AA, ARHGAP32B, VCLB, PARVB, MYO16, SYNPO2LA, SYNPO2LB, MYOZ3A, MYOZ2A, MYOZ2B, MYOZ1A, TPMA, MYOZ1B, ABLIM1A, CORO1CA*	827	118	18868	3.093558502	0.059160439	0.006097662	0.00586
GO:0032982	myosin filament	Cellular Component	7	0.720165	3.68E-04	*SMYHC2, MYHB, MYO18AB, MYO18AA, MYH7BA, MYH7L, MYH14*	827	23	18868	6.943693812	0.107093176	0.010295656	0.009895
GO:0030016	myofibril	Cellular Component	6	0.617284	0.001106	*TMOD1, LMOD2B, TMOD4, TNNT2A, TWF2B, LMOD3*	827	19	18868	7.204734933	0.288790797	0.028383349	0.027278
GO:0031941	filamentous actin	Cellular Component	6	0.617284	0.002246	*PDLIM5B, PDLIM3B, LDB3B, EHBP1L1A, EHBP1L1B, SMTNL1*	827	22	18868	6.222271078	0.499670009	0.053208425	0.051135
GO:0016459	myosin complex	Cellular Component	10	1.028807	0.00248	*SMYHC2, SMYHC3, MYO5AA, MYHB, MYO18AB, MYO18AA, MYH7BA, MYH7L, MYH14, MYO16*	827	67	18868	3.405222978	0.534560246	0.054558852	0.052433
GO:0031463	Cul3-RING ubiquitin ligase complex	Cellular Component	6	0.617284	0.006748	*KLHL15, KLHL21, KLHL13, KLHL40B, KLHL40A, CUL3B*	827	28	18868	4.888927276	0.875745965	0.132589038	0.127423
GO:0005856	cytoskeleton	Cellular Component	43	4.423868	0.006888	*DYRK4, FHOD1, PTPN4A, TUBA8L2, PTPN21, RHOBTB4, HSPB1, LRMP, KRT18A.1, VCLB, CITA, LMOD3, SSH2A, ACTB2, ROCK2A, MID1IP1L, CAPZB, SGCB, DCAF12, KLHL41B, RHOAC, MAP2K6, TMOD1, KLHL41A, ABI2A, TMOD4, CCDC135, TPM1, KLHL21, PARVB, TUBB4B, PTK2AA, TMSB, EPB41L3B, EPB41L3A, DNMBP, LMOD2B, TPMA, TWF2B, TACC2, GAPDH, KANK4, CORO1CA*	827	645	18868	1.520999597	0.881015762	0.132589038	0.127423
GO:0005911	cell-cell junction	Cellular Component	9	0.925926	0.00947	*IGSF11, MPP1, EPB41L3B, USP53B, EPB41L3A, DNMBP, MPP7A, DESMA, LIMS1*	827	68	18868	3.019631553	0.946641091	0.17157736	0.164893
GO:0003779	actin binding	Molecular Function	50	5.144033	2.02E-13	*ABRAB, MYHB, SVILA, ABRAA, SETD3, SSH2A, SYNPO2LA, SYNPO2LB, CAPZB, MYO18AB, TPM3, MYO18AA, PDLIM3B, TPM1, ACTN2B, TMSB, EPB41L3B, EPB41L3A, DAAM2, MYOZ3A, TPMA, CORO1CA, MICAL2A, TNNT2A, ACTN3B, VCLB, SMTNL, MYOZ2A, NRAP, MYOZ2B, XIRP1, FLNCB, MYH14, FLNA, MYO5AA, PDLIM5B, PHACTR3B, NEB, PARVB, MYO16, SMYHC2, SMYHC3, MYH7BA, LDB3B, MYH7L, CAPZA1B, MYOZ1A, MYOZ1B, ABLIM1A, TWF2B*	771	337	17340	3.336835664	1.41E-10	1.41E-10	1.38E-10
GO:0051015	actin filament binding	Molecular Function	36	3.703704	1.19E-09	*MYHB, SVILA, FHOD1, ACTN3B, MYOM1A, VCLB, MYOM1B, CAPZB, SPEGB, NRAP, XIRP1, MYH14, FLNA, FLNCB, TMOD1, MYO5AA, MYO18AB, TPM3, MYO18AA, TMOD4, TNNC1B, TPM1, MYOM2A, NEB, MYO16, ACTN2B, SMYHC2, SMYHC3, MYH7BA, CAPZA1B, MYH7L, TPMA, CORO6, ABLIM1A, TWF2B, CORO1CA*	771	247	17340	3.277934435	8.30E-07	4.15E-07	4.08E-07
GO:0008138	protein tyrosine/serine/threonine phosphatase activity	Molecular Function	12	1.234568	3.50E-05	*EPM2A, DUSP10, SI:CH211-223P8.8, SI:CH211-121A2.2, DUSP22A, SI:CH211-195B15.8, DUSP13A, DUSP27, DUSP16, DUSP22B, PTP4A3A, SSH2A*	771	57	17340	4.734794184	0.024053012	0.008115528	0.007975
GO:0005523	tropomyosin binding	Molecular Function	7	0.720165	6.11E-05	*TMOD1, LMOD2B, TNNT2E, TMOD4, TNNT3B, TNNT2A, LMOD3*	771	17	17340	9.260700389	0.04156231	0.010612357	0.010429
GO:0004879	RNA polymerase II transcription factor activity, ligand-activated sequence-specific DNA binding	Molecular Function	13	1.337449	1.07E-04	*ESR2A, RXRAA, RARAA, PPARDB, RORC, RXRGB, NR1D1, NR2F2, NR2F5, NR2F6B, NR2F1A, RORAA, ESRRGA*	771	75	17340	3.898313878	0.07192424	0.012041058	0.011833
GO:0031433	telethonin binding	Molecular Function	5	0.514403	1.21E-04	*MYOZ3A, MYOZ2A, MYOZ2B, MYOZ1A, MYOZ1B*	771	7	17340	16.06448027	0.080837652	0.012041058	0.011833
GO:0051373	FATZ binding	Molecular Function	5	0.514403	1.21E-04	*MYOZ3A, MYOZ2A, MYOZ2B, MYOZ1A, MYOZ1B*	771	7	17340	16.06448027	0.080837652	0.012041058	0.011833

GO:0003824	catalytic activity	Molecular Function	35	3.600823	1.67E-04	*APOBEC2B, FH, TKTB, AGLA, CKMT2A, GLULB, ACACB, HADHAA, LDHA, HYAL3, ENPP6, CDAB, PFKMB, CKMB, ADSL, PYGMA, CKMA, MDH2, MOCOS, PGAM2, ZGC:172076, PKMA, ALDOAA, ALDOAB, ALDOCB, GOT2B, SUCLA2, IMPDH1A, PCYT1BA, GOT2A, PFKFB4B, SI:DKEY-96F10.1, DUS1L, TPI1B, BCAT1*	771	393	17340	2.002950466	0.109314057	0.014469219	0.014219
GO:0016301	kinase activity	Molecular Function	54	5.555556	2.00E-04	*COQ8AA, MYLK2, DYRK4, CDKN1A, PRKAB1A, MAST2, CKMT2A, AKAP8L, CAMK2N1A, ROCK2A, EEF2K, ULK1B, MYLK4A, PLAUA, PIK3R3B, ADKB, GRK7A, PRKG1B, ADKA, ZGC:172076, HUNK, BMPR1AA, MAPKAPK3, PIK3CA, PRKCQ, MET, UCKL1B, DAPK2A, RAF1A, AK1, CITA, PAK1, INSRA, ERBB2, ABL1, CDKN1CA, MAP2K6, SRPK3, PDK2A, CKMB, PFKMB, CKMA, NEK6, SI:CH211-220I18.4, NEK7, CAMK2B1, PKMA, ALPK3A, PTK2AA, AKT3A, SI:DKEY-8E10.3, MAPKAPK2A, PFKFB4B, SI:DKEY-96F10.1*	771	718	17340	1.691468953	0.129839425	0.015451511	0.015185
GO:0016791	phosphatase activity	Molecular Function	20	2.057613	2.54E-04	*EPM2A, SI:CH211-223P8.8, PTPN4A, PTPN21, PPTC7A, DUSP27, DUSP16, PTP4A3A, SSH2A, PDP1, PTPRNA, CTDSPL2A, DUSP10, SI:CH211-121A2.2, DUSP22A, SI:CH211-195B15.8, CTDSPLB, DUSP13A, FBP2, DUSP22B*	771	173	17340	2.600031488	0.161930356	0.017663163	0.017358
GO:0004721	phosphoprotein phosphatase activity	Molecular Function	17	1.748971	4.52E-04	*EPM2A, SI:CH211-223P8.8, PTPN4A, PTPN21, PPTC7A, DUSP16, CDC25B, SSH2A, PDP1, CTDSPL2A, DUSP10, SI:CH211-121A2.2, DUSP22A, SI:CH211-195B15.8, CTDSPLB, DUSP13A, DUSP22B*	771	139	17340	2.750608851	0.269638051	0.026531485	0.026073
GO:0003700	transcription factor activity, sequence-specific DNA binding	Molecular Function	46	4.73251	4.58E-04	*ESR2A, RXRAA, RARAA, CREB5B, HOXA9B, SIX1B, RORC, MAFAA, RXRGB, NPAS2, ARNTL2, NR2F6B, PBX3A, NFIXA, ZNF395A, FOXO3B, FOXO3A, CREMA, HLFA, PITX3, HOXC4A, NFIL3-6, JUND, ATF5A, PPARDB, TBX3A, RFX2, MAFBA, NFATC1, FOXN3, NR1D1, NR2F2, MITFA, NR2F5, POU3F1, MYCB, TBX15, NFATC3A, RFX6, MYCH, NFIC, CLOCKA, NR2F1A, RORAA, ESRRGA, ATF3*	771	602	17340	1.718525796	0.272725119	0.026531485	0.026073
GO:0016740	transferase activity	Molecular Function	105	10.80247	0.001036	*COQ8AA, UGP2B, MYLK2, TKTB, UBE2D4, PRKAB1A, AGLA, CKMT2A, CAMK2N1A, UBE3A, ROCK2A, ULK1B, HHATLA, PIK3R3B, KCMF1, UBE2E1, BMPR1AA, MAPKAPK3, PRKCQ, HPRT1, ZGC:64002, UCKL1B, LIPT2, DAPK2A, AK1, UBR3, CITA, MOGAT3A, INSRA, ABL1, COX10, MAP2K6, NAA50, SRPK3, NEK6, GFPT2, NEK7, PCIF1, UBE2G1A, CAMK2B1, ALPK3A, CS, SI:DKEY-8E10.3, MAPKAPK2A, PFKFB4B, SI:DKEY-96F10.1, DYRK4, CDKN1A, SETD3, MAST2, PDZRN3B, AKAP8L, SETD7, MTR, CDC34A, SH3RF1, SMG6, DTWD2, GYS1, EEF2K, MYLK4A, NTMT1, PLAUA, DLAT, ZGC:162952, GRK7A, ADKB, PRKG1B, ADKA, ZGC:172076, HUNK, TRPT1, ASH1L, SIRT3, GOT2B, METTL22, PIK3CA, GOT2A, SI:CH211-269C21.2, BCAT1, GAPDH, MET, RAF1A, DLST, TGM2B, RNF2, PAK1, ERBB2, METTL5, GPAT3, CDKN1CA, SMYD1B, PDK2A, CKMB, PYGMA, PFKMB, CKMA, SI:CH211-220I18.4, MOCOS, PKMA, PTK2AA, AKT3A, RNF146, GATM, PCMT*	771	1743	17340	1.354835685	0.513478159	0.055392321	0.054436
GO:0004725	protein tyrosine phosphatase activity	Molecular Function	15	1.54321	0.001778	*EPM2A, SI:CH211-223P8.8, PTPN4A, PTPN21, DUSP16, PTP4A3A, CDC25B, SSH2A, PTPRNA, DUSP10, SI:CH211-121A2.2, DUSP22A, SI:CH211-195B15.8, DUSP13A, DUSP22B*	771	129	17340	2.61514795	0.709717396	0.088271452	0.086747
GO:0000978	RNA polymerase II core promoter proximal region sequence-specific DNA binding	Molecular Function	70	7.201646	0.002018	*HIVEP2A, CREB5B, HOXA9B, ZBTB47B, RORC, MAFAA, RXRGB, PBX3A, ZNF648, FOXO3B, FOXO3A, CREMA, HLFA, PITX3, MYOG, TBX3A, PAX4, RFX2, SOX13, TFEB, ZBTB16A, MAFBA, ZBTB34, MITFA, POU3F1, KLF15, SOX10, EGR2B, MYCB, RFX6, MYCH, MEF2AA, KLF11B, MEF2AB, CLOCKA, DACHC, RORAA, ESRRGA, DACHD, ATF3, ESR2A, RXRAA, RARAA, SIX1B, NPAS2, ARNTL2, NR2F6B, NFIXA, HOXA10B, IRX3A, ZNF395A, HOXC4A, ZBTB18, MLXIP, JUND, SIX4B, PPARDB, NFATC1, NR1D1, NR2F2, NR2F5, KLF4, TBX15, NFATC3A, TFCP2, NFIC, BHLHE40, PROX1A, NR2F1A, MYF5*	771	1095	17340	1.437734307	0.754294653	0.093480387	0.091866
GO:0004111	creatine kinase activity	Molecular Function	4	0.411523	0.00413	*CKMB, CKMA, CKMT2A, ZGC:172076*	771	8	17340	11.24513619	0.943659776	0.179400167	0.176303
GO:0051019	mitogen-activated protein kinase binding	Molecular Function	5	0.514403	0.005778	*DUSP10, MAPKAPK3, MAPKAPK2A, SI:CH211-195B15.8, DUSP16*	771	17	17340	6.614785992	0.982184439	0.236237342	0.232158
