# Supplementary material for: Gene and Allele-Specific Expression Underlying the Electric Signal Divergence in African Weakly Electric Fish
Source: Mol Biol Evol. 2024 Feb 15;41(2):msae021. doi: 10.1093/molbev/msae021 (PMC10897887; doi:10.1093/molbev/msae021)
Supplement: msae021_Supplementary_Data [file msae021_supplementary_data.zip › Cheng-MBE-efishtranscriptomes-Supplementary Table 5 GO terms in group 5 and6.pdf]

Supplementary Table 5 19 Significantly enriched Gene Ontology terms with Fisher's exact test p-value < 0.05 among genes with increasing expression relative to EOD duration (Group 5 and 6).

Term	GO terms	Category	Count	%	P-value	Genes	List Total	Pop Hits	Pop Total	Fold Enrichment	Bonferroni	Benjamini	FDR
GO:0005975	carbohydrate metabolic process	Biological Process	11	4.564315	7.83E-05	*CHST7, GNPDA2, MAN2B2, B3GAT3, GLB1, RPE, SI:DKEY-199F5.8, SPATA20, GUSB, YDJC, HK2*	205	199	18397	4.960583405	0.044687604	0.045715087	0.045715087
GO:0048675	axon extension	Biological Process	4	1.659751	0.010509161	*IST1, UBAP1, PLXNB1B, GRNB*	205	41	18397	8.755264723	0.997908403	1	1
GO:0006914	autophagy	Biological Process	5	2.074689	0.01365593	*BECN1, GABARAPL2, PLEKHM1, WIPI1, MCOLN1A*	205	83	18397	5.406112254	0.999674458	1	1
GO:0007032	endosome organization	Biological Process	3	1.244813	0.022373383	*PLEKHF2, IST1, PI4K2A*	205	21	18397	12.82020906	0.999998176	1	1
GO:0046854	phosphatidylinositol phosphorylation	Biological Process	4	1.659751	0.032820251	*PIP5K1CA, IMPA1, PIK3CG, PI4K2A*	205	63	18397	5.697870693	0.999999997	1	1
GO:0006811	ion transport	Biological Process	13	5.394191	0.041416232	*SLC24A2, SLC12A4, SLC10A7, SLC31A2, KCTD12.1, KCNJ2A, SFXN5B, ATP6V0A1A, TCN2, ATP6V1D, PANX3, MCU, ATP6V1F*	205	614	18397	1.900063558	1	1	1
GO:0006665	sphingolipid metabolic process	Biological Process	3	1.244813	0.043438725	*ARV1, PSAP, SFTPBB*	205	30	18397	8.974146341	1	1	1
GO:0005764	lysosome	Cellular Component	11	4.564315	3.03E-05	*ASAH1B, MAN2B2, BRI3, CTSBA, SMPD1, VPS41, PSAP, PLEKHM1, MCOLN1A, SFTPBB, GUSB*	201	186	18868	5.551489863	0.005199004	0.005212487	0.005182182
GO:0005794	Golgi apparatus	Cellular Component	18	7.46888	3.93E-04	*ZDHHC16B, BECN1, SLC10A7, YIPF3, B3GAT3, CHPF2, ENTPD6, SYAP1, SCOCA, FUT9A, ARV1, MGAT1B, TRAPPC6B, VPS41, SI:DKEY-199F5.8, EXTL3, ZGC:162698, PI4K2A*	201	628	18868	2.690559939	0.065338118	0.033778584	0.033582197
GO:0005768	endosome	Cellular Component	11	4.564315	6.34E-04	*PLEKHF2, BECN1, RAB5B, RAB40C, VPS41, PLEKHM1, UBAP1, FLOT1B, COMMD1, LAMTOR3, PI4K2A*	201	270	18868	3.824359683	0.103297005	0.03633201	0.036120778
GO:0005829	cytosol	Cellular Component	21	8.713693	0.011107253	*BECN1, GABARAPL2, USP7, RIC1, IRS1, RPE, GSR, DNAJB1A, CST14A.2, PPM1AA, WIPI1, SCOCA, PRDX6, HK2, RSPH1, EIF5, UBAP1, OSBPL1A, RAN, ZGC:162698, SPG21*	201	1082	18868	1.821888708	0.853559593	0.477611894	0.47483508
GO:0005773	vacuole	Cellular Component	3	1.244813	0.018737755	*TMEM138, GLB1, LGMN*	201	20	18868	14.08059701	0.961360155	0.595562503	0.592099931
GO:0031410	cytoplasmic vesicle	Cellular Component	8	3.319502	0.020775436	*RAC3A, BECN1, VMA21, TBC1D7, VPS41, FLOT1B, RHOCB, PI4K2A*	201	259	18868	2.899479437	0.972975637	0.595562503	0.592099931
GO:0005581	collagen trimer	Cellular Component	4	1.659751	0.024709798	*COL8A1A, COL12A1A, COL2A1B, COL4A5*	201	59	18868	6.364111645	0.986478523	0.607155038	0.603625067
GO:0005783	endoplasmic reticulum	Cellular Component	15	6.224066	0.03295827	*ZDHHC16B, PLEKHF2, BECN1, SLC10A7, TAPBPL, HSPBP1, AGPAT2, CYP51, ARV1, ZFYVE27, SPTLC1, TRAPPC6B, VMA21, FKBP9, EXTL3*	201	763	18868	1.845425559	0.996862548	0.708602798	0.704483014
GO:0004185	serine-type carboxypeptidase activity	Molecular Function	3	1.244813	6.00E-04	*CTSA, SCPEP1, SI:CH211-122F10.4*	176	4	17340	73.89204545	0.166179401	0.181682518	0.181682518
GO:0004180	carboxypeptidase activity	Molecular Function	3	1.244813	0.02229738	*CTSA, SCPEP1, SI:CH211-122F10.4*	176	23	17340	12.85079051	0.998921912	1	1
GO:0046961	proton-transporting ATPase activity, rotational mechanism	Molecular Function	3	1.244813	0.026095058	*ATP6V0A1A, ATP6V1D, ATP6V1F*	176	25	17340	11.82272727	0.999668472	1	1
GO:0016798	hydrolase activity, acting on glycosyl bonds	Molecular Function	4	1.659751	0.047207987	*MAN2B2, GLB1, SMPD1, GUSB*	176	80	17340	4.926136364	0.999999567	1	1
