# Supplementary material for: Gene and Allele-Specific Expression Underlying the Electric Signal Divergence in African Weakly Electric Fish
Source: Mol Biol Evol. 2024 Feb 15;41(2):msae021. doi: 10.1093/molbev/msae021 (PMC10897887; doi:10.1093/molbev/msae021)
Supplement: msae021_Supplementary_Data [file msae021_supplementary_data.zip › Cheng-MBE-efishtranscriptomes-Supplementary Table 6 GO terms in group 3.pdf]

Supplementary Table 6 41 Significantly enriched Gene Ontology terms with Fisher's exact test p-value < 0.05 among genes with decreasing expression relative to EOD duration (Group 3).

Term	GO terms	Category	Count	%	P-value	Genes	List Total	Pop Hits	Pop Total	Fold Enrichment	Bonferroni	Benjamini	FDR
GO:0007411	axon guidance	Biological Process	12	3.71517	8.12E-05	*SEMA3AB, CNTN1A, SEMA6A, SEMA4D, LAMA1, NPTNB, EPHA4A, SEMA4C, EFNB2A, NRCAMA, TRIOA, DPYSL2B*	271	182	18397	4.47597421	0.055436912	0.057030482	0.056949
GO:0030198	extracellular matrix organization	Biological Process	9	2.786378	7.83E-04	*ADAMTS5, COL5A3A, CCDC80, ADAMTSL4, COL1A1A, COL2A1B, ADAMTS17, ADAMTS9, ADAMTS15A*	271	133	18397	4.593763005	0.423029942	0.274874767	0.274483
GO:0006486	protein glycosylation	Biological Process	9	2.786378	0.001198	*B3GAT1A, GALNT7, NUS1, GALNT14, AARS2, ST8SIA5, FUT8A, LARGE2, ST3GAL2*	271	142	18397	4.302609012	0.568895602	0.280300317	0.279901
GO:0031103	axon regeneration	Biological Process	4	1.23839	0.001825	*B3GAT1A, SEMA4D, LINGO1A, DPYSL2B*	271	17	18397	15.97308444	0.72266857	0.294500541	0.294081
GO:0007155	cell adhesion	Biological Process	16	4.95356	0.002098	*DCHS1B, PCDH15B, EGFL6, LAMA1, PCDH19, CCN2B, CNTN1A, VCANB, EPHA4A, ITGA10, NPTNB, EFNB2A, NCAM1A, ITGB8, FBN2B, BCAR1*	271	437	18397	2.485514283	0.77100365	0.294500541	0.294081
GO:0016310	phosphorylation	Biological Process	21	6.501548	0.004751	*UCK2A, CHKB, MARK3B, DGKB, PFKFB2B, EIF2AK3, DGKZA, NUCKS1A, CDK7, CDK5, EPHB4A, ETNK2, EPHA4A, GRK6, CKBB, SI:CH211-243J20.2, MAP3K8, MAPK14A, TRIOA, PIK3R3B, CAMK1DA*	271	718	18397	1.985512237	0.964684651	0.555914712	0.555123
GO:0008045	motor neuron axon guidance	Biological Process	5	1.547988	0.008075	*SEMA3AB, LAMA1, BICD1A, NOTUM2, NTN2*	271	54	18397	6.285704524	0.996626008	0.809806795	0.808653
GO:0048843	negative regulation of axon extension involved in axon guidance	Biological Process	4	1.23839	0.014473	*SEMA3AB, SEMA6A, SEMA4D, SEMA4C*	271	35	18397	7.758355298	0.999964072	1	1
GO:0007420	brain development	Biological Process	9	2.786378	0.024678	*ADGRL2A, SCGN, CNTN1A, SEMA4D, LAMA1, TAOK2A, NRCAMA, PCDH19, DPYSL2B*	271	238	18397	2.567102856	0.999999976	1	1
GO:0050919	negative chemotaxis	Biological Process	4	1.23839	0.025058	*SEMA3AB, SEMA6A, SEMA4D, SEMA4C*	271	43	18397	6.314940359	0.999999982	1	1
GO:0006468	protein phosphorylation	Biological Process	19	5.882353	0.025768	*ADAM10B, MARK3B, TAOK2A, EIF2AK3, GUCY2F, CDK7, BRSK2B, CDK5, EPHB4A, EPHA4A, GRK6, MAP3K8, MAPK14A, TRIOA, FAM20CB, NRBP2B, CAMK1DA, SI:CH211-1I11.3, MARK1*	271	741	18397	1.740656637	0.999999989	1	1
GO:0035475	angioblast cell migration involved in selective angioblast sprouting	Biological Process	2	0.619195	0.029138	*EPHB4A, EFNB2A*	271	2	18397	67.88560886	0.999999999	1	1
GO:0033334	fin morphogenesis	Biological Process	3	0.928793	0.031137	*FRAS1, COL1A1A, COL2A1B*	271	19	18397	10.71878035	1	1	1
GO:0090630	activation of GTPase activity	Biological Process	5	1.547988	0.033837	*ARHGAP22, SI:CH211-288D18.1, SI:DKEY-191M6.4, SIPA1L1, AGAP3*	271	83	18397	4.089494509	1	1	1
GO:0048013	ephrin receptor signaling pathway	Biological Process	3	0.928793	0.037515	*EPHB4A, EFNB2A, ANKS1AB*	271	21	18397	9.697944122	1	1	1
GO:0071526	semaphorin-plexin signaling pathway	Biological Process	4	1.23839	0.038847	*SEMA3AB, SEMA6A, SEMA4D, SEMA4C*	271	51	18397	5.324361479	1	1	1
GO:0003404	optic vesicle morphogenesis	Biological Process	2	0.619195	0.043388	*EPHA4A, EFNB2A*	271	3	18397	45.25707257	1	1	1
GO:0001756	somitogenesis	Biological Process	5	1.547988	0.046537	*CCDC80, MEF2AA, EFNB2A, MAPK14A, DLC*	271	92	18397	3.689435264	1	1	1
GO:0030903	notochord development	Biological Process	4	1.23839	0.046918	*COL5A3A, EGFL6, LAMA1, COL2A1B*	271	55	18397	4.93713519	1	1	1
GO:0031012	extracellular matrix	Cellular Component	13	4.024768	1.29E-04	*COLEC12, COL5A3A, CCN2B, ADAMTS5, VCANB, ADAMTSL4, COL1A1A, ADAMTS17, COL2A1B, FBN2B, ADAMTS9, LINGO1A, ADAMTS15A*	287	218	18868	3.920404053	0.023360005	0.023635648	0.023636
GO:0005794	Golgi apparatus	Cellular Component	21	6.501548	0.001505	*GALNT7, ARHGAP32A, ADAM10B, GALNT14, AARS2, CLASP1A, SACM1LB, FUT8A, ZDHHC23B, B4GALNT3A, ZDHHC14, B3GAT1A, ST8SIA5, ZGC:162200, FAM20CB, BICD1A, LARGE2, GRINAA, ERGIC1, ST3GAL2, PLA2G4AB*	287	628	18868	2.198384341	0.240907021	0.137711768	0.137712

GO:0016020	membrane	Cellular Component	129	39.93808	0.008157	*ADGRL2A, GALNT14, IFITM1, DGKB, RHOT1B, OLFCS1, XYLT1, ACSL4A, TMEM263, FADS2, SI:CH211-1E14.1, TNFSF11, EHD1B, SI:DKEY-174M14.3, LARGE2, INPP4AB, SYPL2B, ADAM10B, ENTPD2A.1, SEMA6A, NUP210, ATP6AP1A, GPSM2L, FUT8A, TRIM101, SLC5A9, ADAM15, ADGRB2, EPHA4A, MADD, TMCC1B, FAM20CB, GRAMD1BB, OSBPL3B, DIPK1B, WAIF2, AARS2, RPN2, SDC3, MTMR9, SACM1LB, PCDH19, RNF145B, HSD11B2, TMEM248, VPS11, ST8SIA5, GSG1L, SCN3B, ST3GAL2, LINGO1A, NUS1, TMEM132E, ANKRD22, TRPV1, SLC25A25B, FLRT1B, EPHB4A, NPTNB, LRFN1, ITGA10, SPIRE2, TSPAN5A, NCAM1A, SDK1A, LRCH4, TMEM19, MCTP1A, MACF1A, PIGS, KCNK6, TRPC6A, NRCAMA, VSIG10, B4GALNT3A, SPRED2A, SI:CH1073-291C23.2, DGKZA, CATIP, SI:DKEY-112M2.1, CPT2, EFNB2A, ITGB8, GAL3ST4, TMEM119B, DCHS1B, PCDH15B, EGFL6, ANO6, MMEL1, SLC39A14, ZDHHC14, RRBP1A, B3GAT1A, KCNK4A, RAP2B, ECRG4A, TMEM218, BIRC6, DLC, NAT8L, GRINAA, TNFRSF21, ERGIC1, COLEC12, GRAMD1C, LAMA1, PIP4P1A, GUCY2F, ADCY7, PPP1R3AA, IGSF21A, CNTN1A, SI:CH211-278J3.3, GDPD4A, KCNQ5A, KCNQ5B, GALNT7, SLC35A1, SEMA4C, ZDHHC23B, MGAT4C, SPCS2, JAGN1B, FRAS1, GDPD5B, EPPK1, SI:CH73-267C23.10, GPR146*	287	7112	18868	1.192454819	0.776603689	0.487709159	0.487709
GO:0005576	extracellular region	Cellular Component	27	8.359133	0.01066	*LAMA1, IGFBP6B, CXCL19, OLFM3A, FGF2, HTRA1B, NTN2, CXCL18A.1, SEMA3AB, ADAMTS5, GLIPR2, ADAMTSL4, COL1A1A, ADAMTS17, ENPP2, FBN2B, NOTUM2, ADAMTS9, MARK1, ADAMTS15A, COL5A3A, CCN2B, VCANB, CCDC80, DKK3A, ECRG4A, COL2A1B*	287	1059	18868	1.676145729	0.859325897	0.487709159	0.487709
GO:0043231	intracellular membrane-bounded organelle	Cellular Component	11	3.405573	0.024173	*EPN3A, HSD11B2, SPCS2, PHLPP1, HIP1RB, PDXDC1, DHRS9, EHD1B, NAT8L, RHOBTB1, OSBPL3B*	287	319	18868	2.266971044	0.98864384	0.884730212	0.88473
GO:0000139	Golgi membrane	Cellular Component	11	3.405573	0.03016	*B3GAT1A, GALNT7, SLC35A1, GALNT14, AARS2, XYLT1, SACM1LB, ZDHHC23B, LARGE2, ERGIC1, ZDHHC14*	287	331	18868	2.184784783	0.996317753	0.886341935	0.886342
GO:0015629	actin cytoskeleton	Cellular Component	6	1.857585	0.033904	*MTSS1LA, ARHGAP32A, CATIP, HAX1, MYO5B, MACF1A*	287	118	18868	3.342821709	0.998185616	0.886341935	0.886342
GO:0005930	axoneme	Cellular Component	4	1.23839	0.042102	*HYDIN, CFAP36, CFAP206, DNALI1*	287	51	18868	5.156247865	0.999618585	0.91141975	0.91142
GO:0005938	cell cortex	Cellular Component	5	1.547988	0.044824	*ARHGAP32A, SPIRE2, GPSM2L, RHOBTB1, MACF1A*	287	88	18868	3.735350016	0.999773395	0.91141975	0.91142
GO:0005509	calcium ion binding	Molecular Function	24	7.430341	4.28E-04	*DCHS1B, SNED1, PCDH15B, DIPK1B, CALM1A, EGFL6, DGKB, RHOT1B, PDCD6, ACTN1, PCDH19, SLC25A25B, EDIL3A, SCGN, VCANB, PPP3R1B, EFHD2, ENPP2, DLC, FBN2B, EHD1B, MCTP1A, MACF1A, PLA2G4AB*	250	739	17340	2.25255751	0.139265272	0.149936794	0.149937
GO:0004222	metalloendopeptidase activity	Molecular Function	8	2.47678	0.001912	*ADAMTS5, ADAM10B, ADAM15, ADAMTSL4, ADAMTS17, MMEL1, ADAMTS9, ADAMTS15A*	250	122	17340	4.548196721	0.488191854	0.323477718	0.323478
GO:0016740	transferase activity	Molecular Function	40	12.3839	0.003459	*UBE2NB, UGP2B, UCK2A, GALNT14, AARS2, DGKB, PFKFB2B, XYLT1, B4GALNT3A, DGKZA, SI:CH211-278J3.3, CPT2, GRK6, ST8SIA5, SI:CH211-243J20.2, MAP3K8, TRIOA, LARGE2, PIK3R3B, ST3GAL2, GALNT7, NUS1, CHKB, MARK3B, EIF2AK3, FUT8A, ZDHHC23B, ZDHHC14, MGAT4C, B3GAT1A, NUCKS1A, CDK7, CDK5, ETNK2, EPHB4A, EPHA4A, CKBB, MAPK14A, NAT8L, CAMK1DA*	250	1743	17340	1.591738382	0.702598533	0.323477718	0.323478
GO:0016301	kinase activity	Molecular Function	21	6.501548	0.003697	*UCK2A, CHKB, MARK3B, DGKB, PFKFB2B, EIF2AK3, DGKZA, NUCKS1A, CDK7, CDK5, EPHB4A, ETNK2, EPHA4A, GRK6, CKBB, SI:CH211-243J20.2, MAP3K8, MAPK14A, TRIOA, PIK3R3B, CAMK1DA*	250	718	17340	2.028635097	0.726460298	0.323477718	0.323478
GO:0030215	semaphorin receptor binding	Molecular Function	4	1.23839	0.01261	*SEMA3AB, SEMA6A, SEMA4D, SEMA4C*	250	34	17340	8.16	0.988223357	0.778839411	0.778839
GO:0045499	chemorepellent activity	Molecular Function	4	1.23839	0.014735	*SEMA3AB, SEMA6A, SEMA4D, SEMA4C*	250	36	17340	7.706666667	0.994458678	0.778839411	0.778839
GO:0016757	transferase activity, transferring glycosyl groups	Molecular Function	10	3.095975	0.019346	*B3GAT1A, GALNT7, MGAT4C, GALNT14, AARS2, ST8SIA5, XYLT1, FUT8A, LARGE2, ST3GAL2*	250	278	17340	2.494964029	0.998927326	0.778839411	0.778839
GO:0005178	integrin binding	Molecular Function	5	1.547988	0.01995	*CCN2B, EGFL6, ADAM15, ITGA10, ITGB8*	250	72	17340	4.816666667	0.999135338	0.778839411	0.778839
GO:0050321	tau-protein kinase activity	Molecular Function	3	0.928793	0.021586	*BRSK2B, MARK3B, MARK1*	250	16	17340	13.005	0.999518189	0.778839411	0.778839
GO:0005524	ATP binding	Molecular Function	35	10.83591	0.022253	*UBE2NB, UCK2A, AARS2, DGKB, PFKFB2B, TAOK2A, GUCY2F, SMC4, ADCY7, DGKZA, SMCHD1, SI:CH211-257P13.3, GRK6, DHX15, SI:CH211-243J20.2, MAP3K8, TRIOA, EHD1B, NRBP2B, SI:CH211-1I11.3, MARK1, ACTR3, MARK3B, ENTPD2A.1, STARD9, EIF2AK3, CDK7, BRSK2B, CDK5, EPHB4A, EPHA4A, MYO5B, CKBB, MAPK14A, CAMK1DA*	250	1662	17340	1.460649819	0.999620387	0.778839411	0.778839
GO:0016758	transferase activity, transferring hexosyl groups	Molecular Function	5	1.547988	0.032802	*GALNT7, GALNT14, AARS2, LARGE2, B4GALNT3A*	250	84	17340	4.128571429	0.99999148	1	1

GO:0005085	guanyl-nucleotide exchange factor activity	Molecular Function	8	2.47678	0.03611	*PREX2, BCAR3, RABGEF1, CCDC88C, MADD, ARHGEF9B, TRIOA, RAPGEFL1*	250	215	17340	2.580837209	0.999997432	1	1
GO:0005201	extracellular matrix structural constituent	Molecular Function	5	1.547988	0.044995	*COL5A3A, LAMA1, COL1A1A, COL2A1B, FBN2B*	250	93	17340	3.729032258	0.9999999	1	1
