# Supplementary material for: Gene and Allele-Specific Expression Underlying the Electric Signal Divergence in African Weakly Electric Fish
Source: Mol Biol Evol. 2024 Feb 15;41(2):msae021. doi: 10.1093/molbev/msae021 (PMC10897887; doi:10.1093/molbev/msae021)
Supplement: msae021_Supplementary_Data [file msae021_supplementary_data.zip › Cheng-MBE-efishtranscriptomes-Supplementary Table 7 Imbalanced alleles.pdf]

Supplementary Table 7 Imbalanced expressed alleles and their *C. compressirostris* allele proportion for all five replicates of any hybrid cohort.

**Hybrid cohort**	**Tissue**	**SNPs ID**	**Gene ID in Annotation**	**Proportion 1**	**Proportion 2**	**Proportion 3**	**Proportion 4**	**Proportion 5**	**Average Proportion (95% Confidence Limits)**	**Gene**	**Hightlights of Predicted Function**	**Gene Description**
com x rhy	EO	1665681	maker-ptg000267l-snap-gene-2.67-mRNA-1	0.64	0.63	0.64	0.7	0.67	0.66 (0.62-0.369)	*ANKRD12*	ankyrin repeats-containing cofactor	ankyrin repeat domain 12
com x rhy	EO	681658	maker-ptg000082l-snap-gene-20.7-mRNA-1	0.64	0.61	0.74	0.68	0.73	0.68 (0.61-0.75)	*ARL13b*	cilium-specific protein	ADP ribosylation factor like GTPase 13B
com x rhy	EO	2982031	maker-ptg000598l-snap-gene-2.10-mRNA-1	0.65	0.79	0.81	0.68	0.67	0.72 (0.63-0.81)	*CDH15*	calcium-dependent cell adhesion protein	cadherin 15
com x rhy	EO	1098949	maker-ptg000160l-snap-gene-11.44-mRNA-1	0.86	0.82	0.79	0.81	0.79	0.82 (0.78-0.85)	*CHRND*	opening of an ion-conducting channel across the plasma membrane.	cholinergic receptor nicotinic delta subunit
com x rhy	EO	4997264	maker-ptg001536l-augustus-gene-4.34-mRNA-1	0.61	0.61	0.79	0.6	0.64	0.65 (0.60-0.71)	*COL6a3*	cell-binding protein	collagen type VI alpha 3 chain
com x rhy	EO	4997265	maker-ptg001536l-augustus-gene-4.34-mRNA-1	0.61	0.62	0.8	0.6	0.64				
com x rhy	EO	4531902	maker-ptg001236l-augustus-gene-26.64-mRNA-1	0.71	0.75	0.79	0.74	0.66	0.73 (0.67-0.79)	*CRELD1*	epidermal growth factor-related proteins	cysteine rich with EGF like domains 1
com x rhy	EO	4531904	maker-ptg001236l-augustus-gene-26.64-mRNA-1	0.81	0.6	0.65	0.89	0.7				
com x rhy	EO	1396998	maker-ptg000216l-augustus-gene-4.4-mRNA-1	0.67	0.6	0.64	0.61	0.6	0.62 (0.59-0.66)	*DAG1*	laminin and basement membrane assembly	dystroglycan 1
com x rhy	EO	4392819	snap_masked-ptg001165l-processed-gene-0.145-mRNA-1	0.65	0.74	0.67	0.77	0.74	0.71 (0.65-0.78)	*DCAF6*	ligand-dependent coactivator of nuclear receptors	DDB1 and CUL4 associated factor 6
com x rhy	EO	837824	maker-ptg000110l-snap-gene-11.31-mRNA-1	0.67	0.61	0.71	0.61	0.61	0.64 (0.58-0.70)	*DST*	cytoskeletal linker protein	dystonin
com x rhy	EO	3404159	maker-ptg000729l-snap-gene-5.79-mRNA-1	0.76	0.8	0.7	0.69	0.84	0.74 (0.69-0.79)	*ENPP2*	phosphodiesterase	ectonucleotide pyrophosphatase/phosphodiesterase 2
com x rhy	EO	3404158	maker-ptg000729l-snap-gene-5.79-mRNA-1	0.73	0.67	0.68	0.7	0.84				
com x rhy	EO	3813376	maker-ptg000876l-snap-gene-1.7-mRNA-1	0.66	0.67	0.71	0.63	0.6	0.65 (0.60-0.71)	*HOXC11a*	multicellular organism development and regulation of transcription	homeobox C11a
com x rhy	EO	1651431	maker-ptg000265l-est_gff_est2genome-gene-6.33-mRNA-1	0.2	0.18	0.13	0.14	0.12	0.16 (0.11-0.20)	*KCNJ2*	allow potassium to flow into a cell rather than out of a cell, probably participates in establishing action potential waveform	inward rectifier potassium channel 2
com x rhy	EO	768530	snap_masked-ptg000100l-processed-gene-4.104-mRNA-1	0.81	0.69	0.77	0.88	0.77	0.71 (0.64-0.78)	*OBSCN*	structural component of striated muscles which plays a role in myofibrillogenesis	obscurin, cytoskeletal calmodulin and titin-interacting RhoGEF
com x rhy	EO	768526	snap_masked-ptg000100l-processed-gene-4.104-mRNA-1	0.62	0.62	0.6	0.67	0.68				
com x rhy	EO	1624861	maker-ptg000254l-augustus-gene-4.109-mRNA-1	0.69	0.9	0.68	0.9	0.73	0.78 (0.64-0.92)	*PALMD*	regulation of cell shape	palmdelphin
com x rhy	EO	2766503	maker-ptg000548l-snap-gene-0.90-mRNA-1	0.62	0.78	0.63	0.67	0.76	0.69 (0.60-0.78)	*PNKD*	regulation of myofibrillogenesis	paroxysmal nonkinesigenic dyskinesia
com x rhy	EO	4446575	maker-ptg001188l-snap-gene-6.4-mRNA-1	0.66	0.63	0.61	0.69	0.69	0.66 (0.64-0.68)	*SCN4aa*	voltage-gated sodium channel	sodium channel protein type 4 subunit alpha A
com x rhy	EO	4446574	maker-ptg001188l-snap-gene-6.4-mRNA-1	0.65	0.68	0.67	0.66	0.63				
com x tsh	EO	2670824	maker-ptg000509l-augustus-gene-3.41-mRNA-1	0.91	0.85	0.78	0.92	0.86	0.86 (0.79-0.93)	*TSPAN7b*	integral component of plasma membrane	tetraspanin 7b
com x tsh	EO	5058039	maker-ptg001586l-snap-gene-1.60-mRNA-1	0.94	0.71	0.82	0.9	0.9	0.84 (0.77-0.91)	*UACA*	modulates isoactin dynamics to regulate the morphological alterations required for cell growth and motility	uveal autoantigen with coiled-coil domains and ankyrin repeats
com x tsh	EO	5058040	maker-ptg001586l-snap-gene-1.60-mRNA-1	0.91	0.68	0.77	0.9	0.87				
com x rhy	SM	3177368	maker-ptg000959l-snap-gene-7.19-mRNA-1	0.72	0.6	0.67	0.67	0.68	0.67 (0.61-0.72)	*PLPP3*	membrane glycoprotein at the cell plasma membrane	phospholipid phosphatase 3
com x rhy	SM	305826	maker-ptg000051l-snap-gene-6.91-mRNA-1	0.73	0.67	0.61	0.68	0.64	0.67 (0.61-0.72)	*HSP90aa1*	inducible molecular chaperone	heat shock protein 90 alpha family class a member 1
com x rhy	SM	3786101	maker-ptg001394l-snap-gene-3.24-mRNA-1	0.66	0.7	0.65	0.8	0.6	0.68 (0.59-0.78)	*MYO18a*	unconventional myosin	myosin XVIIIA
com x rhy	SM	4016401	maker-ptg001619l-snap-gene-1.135-mRNA-1	0.65	0.75	0.79	0.64	0.72	0.71 (0.63-0.79)	*TRIM63*	muscle-specific rING finger protein	E3 ubiquitin-protein ligase TRIM63
com x rhy	SM	862477	maker-ptg000160l-snap-gene-11.44-mRNA-1	0.8	0.8	0.7	0.69	0.83	0.76 (0.68-0.84)	*CHRND*	opening of an ion-conducting channel across the plasma membrane	cholinergic receptor nicotinic delta subunit
com x rhy	SM	606006	snap_masked-ptg000100l-processed-gene-4.104-mRNA-1	0.84	0.7	0.83	0.84	0.75	0.79 (0.71-0.87)	*OBSCN*	structural component of striated muscles which plays a role in myofibrillogenesis	obscurin, cytoskeletal calmodulin and titin-interacting RhoGEF
com x rhy	SM	3426233	maker-ptg001156l-augustus-gene-8.0-mRNA-1	0.91	0.9	0.92	0.95	0.92	0.92 (0.90-0.94)	*CASQ1a*	skeletal muscle specific member of the calsequestrin protein	calsequestrin-1a
com x tsh	SM	1814167	maker-ptg000413l-snap-gene-5.117-mRNA-1	0.77	0.86	0.73	0.78	0.81	0.79 (0.73-0.85)	*XIRP1*	protect actin filaments during depolymerization	xin actin-binding repeat-containing protein 1
