# Supplementary material for: Gene and Allele-Specific Expression Underlying the Electric Signal Divergence in African Weakly Electric Fish
Source: Mol Biol Evol. 2024 Feb 15;41(2):msae021. doi: 10.1093/molbev/msae021 (PMC10897887; doi:10.1093/molbev/msae021)
Supplement: msae021_Supplementary_Data [file msae021_supplementary_data.zip › Cheng-MBE-efishtranscriptomes-Supplementary Table 8 Transcript sequence of KCNJ2 gene.pdf]

Supplementary Table 8 Sequence information of *KCNJ2* transcripts in all pure-bred individuals

Species	Sequence
com1	ATGAATGTCCAAAACTGTTCCTCAGAAGGTTCTTCCAAAAACTTTTCCAAGGCAGCAGAAGTGAAGCCCTCATCAGCAGAA<br>GCGATGGGTAGTGTGCGGGCCAGCCGCTACAGCGTCGTGTCCTCCAAAGTAGATGGCCTCAAGTTGGCCACTGTGGCCGT<br>GTCCAATGGCCACAGCAATGGTGATGGCAAGGTGAACATGTGGCAGCCGGTGCCATGTCGTTTCGTCAAGAAGGATGGA<br>CACTGCAACGTGCACATCATCAACATGAGCGAGAAAGGCCAGCGCTACATAGCCGACATCTTCACCACCTGCGTGGACATC<br>CGTTGGCGATGGATGATAATCATCTTCTGCTTGACTTTTGTGCTTTCATGGTTGTTCTTTGGCTATGTGTTCTGGCTGGTGG<br>CCTTCTTCTATGGTGACTTGGGGAATAGCTCCCAGCAGTGTGTCTCCAATGTCAACAGCTTCATGGCAGCCTTCCTCTTCTCT<br>GTGGAGACGCAGACCACTATTGGCTATGGTTACCACCATGTGACAGAAGAGTGCCCCATCGCTGTCTTTATGGTGGTTTTC<br>CAGTGCATTGTTGGCTGCATCATCGACGCCTTCATCATTGGTGCCGTCATGGCCAAGATGGCCAAGCCCACGAAGCGCAAT<br>GAAACCCTGGTGTTTAGCCACAACGCTACAATAGCAATGCGGGACGGCAAGCTATGCCTGATGTGGCGAGTTGGCAACCT<br>ACGCAAAAGCCACCTGGTGGAGGCCCACGTGAGGGCTCAGCTACTCAAGTCCCGGACCACCGCCGAGGGGGAGTTTATC<br>CCCCTAGACCACGTAGATATTGATGTGGGCTTTGACACTGGCGTAGACCGGATCTTCCTTGTTTCCCCCATCACCATTGTCC<br>ATGAGATCAACGAGGACAGTCCCTTCTATGATATGAGCAAGCAGGATTTTGAGACTGCTGGATTTGAGATTGTGGTCATCC<br>TGGAGGGCATGGTAGAAGCCACAGCCATGACAACCCAGTGTCGCAGTTCCTACCTGGCAGGGGAGATCCTCTGGGGACA<br>CTGCTTCGAGCCTGTACTCTTTGAGGAGAAGAACTACTACAAGGTCGACTACTCTCATTTCCACAAAACCTACGAGGTGCC<br>GAGCACTCCGCTATGTAGTGCGCGGGAGCTTGCTGAAAAGAAGGATAATGAGTCCAGCTCTAACTCTTTTTGCTATGAGA<br>ATGAAGTGGCGATGATGGACAAAGAGGAGACGGAGGACAAAAGCGAGTGCAGCAATGATGGGAGCAGTTCACAAAAGG<br>CTTCAGAGTTGGGGCGCAATCTCTTCATGACGTTTAGACGAGAATCTGAGATTTGA

com2

ATGAATGTCCAAAACTGTTCCTCAGAAGGTTCTTCCAAAAACTTTTCCAAGGCAGCAGAAGTGAAGCCCTCATCAGCAGAA
GCGATGGGTAGTGTGCGGGCCAGCCGCTACAGCGTCGTGTCCTCCAAAGTAGATGGCCTCAAGTTGGCCACTGTGGCCGT
GTCCAATGGCCACAGCAATGGTGATGGCAAGGTGAACATGTGGCAGCCGGTGCCATGTCGTTTCGTCAAGAAGGATGGA
CACTGCAACGTGCACATCATCAACATGAGCGAGAAAGGCCAGCGCTACATAGCCGACATCTTCACCACCTGCGTGGACATC
CGTTGGCGATGGATGATAATCATCTTCTGCTTGACTTTTGTGCTTTCATGGTTGTTCTTTGGCTATGTGTTCTGGCTGGTGG
CCTTCTTCTATGGTGACTTGGGGAATAGCTCCCAGCAGTGTGTCTCCAATGTCAACAGCTTCATGGCAGCCTTCCTCTTCTCT
GTGGAGACGCAGACCACTATTGGCTATGGTTACCACCATGTGACAGAAGAGTGCCCCATCGCTGTCTTTATGGTGGTTTTC
CAGTGCATTGTTGGCTGCATCATCGACGCCTTCATCATTGGTGCCGTCATGGCCAAGATGGCCAAGCCCACGAAGCGCAAT
GAAACCCTGGTGTTTAGCCACAACGCTACAATAGCAATGCGGGACGGCAAGCTATGCCTGATGTGGCGAGTTGGCAACCT
ACGCAAAAGCCACCTGGTGGAGGCCCACGTGAGGGCTCAGCTACTCAAGTCCCGGACCACCGCCGAGGGGGAGTTTATC
CCCCTAGACCACGTAGATATTGATGTGGGCTTTGACACTGGCGTAGACCGGATCTTCCTTGTTTCCCCCATCACCATTGTCC
ATGAGATCAACGAGGACAGTCCCTTCTATGATATGAGCAAGCAGGATTTTGAGACTGCTGGATTTGAGATTGTGGTCATCC
TGGAGGGCATGGTAGAAGCCACAGCCATGACAACCCAGTGTCGCAGTTCCTACCTGGCAGGGGAGATCCTCTGGGGACA
CTGCTTCGAGCCTGTACTCTTTGAGGAGAAGAACTACTACAAGGTCGACTACTCTCATTTCCACAAAACCTACGAGGTGCC
GAGCACTCCGCTATGTAGTGCGCGGGAGCTTGCTGAAAAGAAGGATAATGAGTCCAGCTCTAACTCTTTTTGCTATGAGA
ATGAAGTGGCGATGATGGACAAAGAGGAGACGGAGGACAAAAGCGAGTGCAGCAATGATGGGAGCAGTTCACAAAAGG
CTTCAGAGTTGGGGCGCAATCTCTTCATGACGTTTAGACGAGAATCTGAGATTTGA

com3

ATGAATGTCCAAAACTGTTCCTCAGAAGGTTCTTCCAAAAACTTTTCCAAGGCAGCAGAAGTGAAGCCCTCATCAGCAGAA
GCGATGGGTAGTGTGCGGGCCAGCCGCTACAGCGTCGTGTCCTCCAAAGTAGATGGCCTCAAGTTGGCCACTGTGGCCGT
GTCCAATGGCCACAGCAATGGTGATGGCAAGGTGAACATGTGGCAGCCGGTGCCATGTCGTTTCGTCAAGAAGGATGGA
CACTGCAACGTGCACATCATCAACATGAGCGAGAAAGGCCAGCGCTACATAGCCGACATCTTCACCACCTGCGTGGACATC
CGTTGGCGATGGATGATAATCATCTTCTGCTTGACTTTTGTGCTTTCATGGTTGTTCTTTGGCTATGTGTTCTGGCTGGTGG
CCTTCTTCTATGGTGACTTGGGGAATAGCTCCCAGCAGTGTGTCTCCAATGTCAACAGCTTCATGGCAGCCTTCCTCTTCTCT
GTGGAGACGCAGACCACTATTGGCTATGGTTACCACCATGTGACAGAAGAGTGCCCCATCGCTGTCTTTATGGTGGTTTTC
CAGTGCATTGTTGGCTGCATCATCGACGCCTTCATCATTGGTGCCGTCATGGCCAAGATGGCCAAGCCCACGAAGCGCAAT
GAAACCCTGGTGTTTAGCCACAACGCTACAATAGCAATGCGGGACGGCAAGCTATGCCTGATGTGGCGAGTTGGCAACCT
ACGCAAAAGCCACCTGGTGGAGGCCCACGTGAGGGCTCAGCTACTCAAGTCCCGGACCACCGCCGAGGGGGAGTTTATC
CCCCTAGACCACGTAGATATTGATGTGGGCTTTGACACTGGCGTAGACCGGATCTTCCTTGTTTCCCCCATCACCATTGTCC
ATGAGATCAACGAGGACAGTCCCTTCTATGATATGAGCAAGCAGGATTTTGAGACTGCTGGATTTGAGATTGTGGTCATCC
TGGAGGGCATGGTAGAAGCCACAGCCATGACAACCCAGTGTCGCAGTTCCTACCTGGCAGGGGAGATCCTCTGGGGACA
CTGCTTCGAGCCTGTACTCTTTGAGGAGAAGAACTACTACAAGGTCGACTACTCTCATTTCCACAAAACCTACGAGGTGCC
GAGCACTCCGCTATGTAGTGCGCGGGAGCTTGCTGAAAAGAAGGATAATGAGTCCAGCTCTAACTCTTTTTGCTATGAGA
ATGAAGTGGCGATGATGGACAAAGAGGAGACGGAGGACAAAAGCGAGTGCAGCAATGATGGGAGCAGTTCACAAAAGG
CTTCAGAGTTGGGGCGCAATCTCTTCATGACGTTTAGACGAGAATCTGAGATTTGA

com4 ATGAATGTCCAAAACTGTTCCTCAGAAGGTTCTTCCAAAAACTTTTCCAAGGCAGCAGAAGTGAAGCCCTCATCAGCAGAA
GCGATGGGTAGTGTGCGGGCCAGCCGCTACAGCGTCGTGTCCTCCAAAGTAGATGGCCTCAAGTTGGCCACTGTGGCCGT
GTCCAATGGCCACAGCAATGGTGATGGCAAGGTGAACATGTGGCAGCCGGTGCCATGTCGTTTCGTCAAGAAGGATGGA
CACTGCAACGTGCACATCATCAACATGAGCGAGAAAGGCCAGCGCTACATAGCCGACATCTTCACCACCTGCGTGGACATC
CGTTGGCGATGGATGATAATCATCTTCTGCTTGACTTTTGTGCTTTCATGGTTGTTCTTTGGCTATGTGTTCTGGCTGGTGG
CCTTCTTCTATGGTGACTTGGGGAATAGCTCCCAGCAGTGTGTCTCCAATGTCAACAGCTTCATGGCAGCCTTCCTCTTCTCT
GTGGAGACGCAGACCACTATTGGCTATGGTTACCACCATGTGACAGAAGAGTGCCCCATCGCTGTCTTTATGGTGGTTTTC
CAGTGCATTGTTGGCTGCATCATCGACGCCTTCATCATTGGTGCCGTCATGGCCAAGATGGCCAAGCCCACGAAGCGCAAT
GAAACCCTGGTGTTTAGCCACAACGCTACAATAGCAATGCGGGACGGCAAGCTATGCCTGATGTGGCGAGTTGGCAACCT
ACGCAAAAGCCACCTGGTGGAGGCCCACGTGAGGGCTCAGCTACTCAAGTCCCGGACCACCGCCGAGGGGGAGTTTATC
CCCCTAGACCACGTAGATATTGATGTGGGCTTTGACACTGGCGTAGACCGGATCTTCCTTGTTTCCCCCATCACCATTGTCC
ATGAGATCAACGAGGACAGTCCCTTCTATGATATGAGCAAGCAGGATTTTGAGACTGCTGGATTTGAGATTGTGGTCATCC
TGGAGGGCATGGTAGAAGCCACAGCCATGACAACCCAGTGTCGCAGTTCCTACCTGGCAGGGGAGATCCTCTGGGGACA
CTGCTTCGAGCCTGTACTCTTTGAGGAGAAGAACTACTACAAGGTCGACTACTCTCATTTCCACAAAACCTACGAGGTGCC
GAGCACTCCGCTATGTAGTGCGCGGGAGCTTGCTGAAAAGAAGGATAATGAGTCCAGCTCTAACTCTTTTTGCTATGAGA
ATGAAGTGGCGATGATGGACAAAGAGGAGACGGAGGACAAAAGCGAGTGCAGCAATGATGGGAGCAGTTCACAAAAGG
CTTCAGAGTTGGGGCGCAATCTCTTCATGACGTTTAGACGAGAATCTGAGATTTGA

com5 ATGAATGTCCAAAACTGTTCCTCAGAAGGTTCTTCCAAAAACTTTTCCAAGGCAGCAGAAGTGAAGCCCTCATCAGCAGAA
GCGATGGGTAGTGTGCGGGCCAGCCGCTACAGCGTCGTGTCCTCCAAAGTAGATGGCCTCAAGTTGGCCACTGTGGCCGT
GTCCAATGGCCACAGCAATGGTGATGGCAAGGTGAACATGTGGCAGCCGGTGCCATGTCGTTTCGTCAAGAAGGATGGA
CACTGCAACGTGCACATCATCAACATGAGCGAGAAAGGCCAGCGCTACATAGCCGACATCTTCACCACCTGCGTGGACATC
CGTTGGCGATGGATGATAATCATCTTCTGCTTGACTTTTGTGCTTTCATGGTTGTTCTTTGGCTATGTGTTCTGGCTGGTGG
CCTTCTTCTATGGTGACTTGGGGAATAGCTCCCAGCAGTGTGTCTCCAATGTCAACAGCTTCATGGCAGCCTTCCTCTTCTCT
GTGGAGACGCAGACCACTATTGGCTATGGTTACCACCATGTGACAGAAGAGTGCCCCATCGCTGTCTTTATGGTGGTTTTC
CAGTGCATTGTTGGCTGCATCATCGACGCCTTCATCATTGGTGCCGTCATGGCCAAGATGGCCAAGCCCACGAAGCGCAAT
GAAACCCTGGTGTTTAGCCACAACGCTACAATAGCAATGCGGGACGGCAAGCTATGCCTGATGTGGCGAGTTGGCAACCT
ACGCAAAAGCCACCTGGTGGAGGCCCACGTGAGGGCTCAGCTACTCAAGTCCCGGACCACCGCCGAGGGGGAGTTTATC
CCCCTAGACCACGTAGATATTGATGTGGGCTTTGACACTGGCGTAGACCGGATCTTCCTTGTTTCCCCCATCACCATTGTCC
ATGAGATCAACGAGGACAGTCCCTTCTATGATATGAGCAAGCAGGATTTTGAGACTGCTGGATTTGAGATTGTGGTCATCC
TGGAGGGCATGGTAGAAGCCACAGCCATGACAACCCAGTGTCGCAGTTCCTACCTGGCAGGGGAGATCCTCTGGGGACA
CTGCTTCGAGCCTGTACTCTTTGAGGAGAAGAACTACTACAAGGTCGACTACTCTCATTTCCACAAAACCTACGAGGTGCC
GAGCACTCCGCTATGTAGTGCGCGGGAGCTTGCTGAAAAGAAGGATAATGAGTCCAGCTCTAACTCTTTTTGCTATGAGA
ATGAAGTGGCGATGATGGACAAAGAGGAGACGGAGGACAAAAGCGAGTGCAGCAATGATGGGAGCAGTTCACAAAAGG
CTTCAGAGTTGGGGCGCAATCTCTTCATGACGTTTAGACGAGAATCTGAGATTTGA

tsh1 ATGAATGTCCAAAACTGTTCCTCAGAAGGTTCTTCCAAAAACTTTTCCAAGGCAGCAGAAGTGAAGCCCTCATCAGCAGAA
GCGATGGGTAGTGTGCGGGCCAGCCGCTACAGCGTCGTGTCCTCCAAAGTAGATGGCCTCAAGTTGGCCACTGTGGCCGT
GTCCAATGGCCACAGCAATGGTGATGGCAAGGTGAACATGTGGCAGCCGGTGCCATGTCGTTTCGTCAAGAAGGATGGA
CACTGCAACGTGCACATCATCAACATGAGCGAGAAAGGCCAGCGCTACATAGCCGACATCTTCACCACCTGCGTGGACATC
CGTTGGCGATGGATGATAATCATCTTCTGCTTGACTTTTGTGCTTTCATGGTTGTTCTTTGGCTATGTGTTCTGGCTGGTGG
CCTTCTTCTATGGTGACTTGGGGAATAGCTCCCAGCAGTGTGTCTCCAATGTCAACAGCTTCATGGCAGCCTTCCTCTTCTCT
GTGGAGACGCAGACCACTATTGGCTATGGTTACCACCATGTGACAGAAGAGTGCCCCATCGCTGTCTTTATGGTGGTTTTC
CAGTGCATTGTTGGCTGCATCATCGACGCCTTCATCATTGGTGCCGTCATGGCCAAGATGGCCAAGCCCACGAAGCGCAAT
GAAACCCTGGTGTTTAGCCACAACGCTACAATAGCAATGCGGGACGGCAAGCTATGCCTGATGTGGCGAGTTGGCAACCT
ACGCAAAAGCCACCTGGTGGAGGCCCACGTGAGGGCTCAGCTACTCAAGTCCCGGACCACCGCCGAGGGGGAGTTTATC
CCCCTAGACCACGTAGATATTGATGTGGGCTTTGACACTGGCGTAGACCGGATCTTCCTTGTTTCCCCCATCACCATTGTCC
ATGAGATCAACGAGGACAGTCCCTTCTATGATATGAGCAAGCAGGATTTTGAGACTGCTGGATTTGAGATTGTGGTCATCC
TGGAGGGCATGGTAGAAGCCACAGCCATGACAACCCAGTGTCGCAGTTCCTACCTGGCAGGGGAGATCCTCTGGGGACA
CTGCTTCGAGCCTGTACTCTTTGAGGAGAAGAACTACTACAAGGTCGACTACTCTCATTTCCACAAAACCTACGAGGTGCC
GAGCACTCCGCTATGTAGTGCGCGGGAGCTTGCTGAAAAGAAGGATAATGAGTCCAGCTCTAACTCTTTTTGCTATGAGA
ATGAAGTGGCGATGATGGACAAAGAGGAGACGGAGGACAAAAGCGAGTGCAGCAATGATGGGAGCAGTTCACAAAAGG
CTTCAGAGTTGGGGCGCAATCTCTTCATGACGTTTAGACGAGAATCTGAGATTTGA

tsh2 ATGAATGTCCAAAACTGTTCCTCAGAAGGTTCTTCCAAAAACTTTTCCAAGGCAGCAGAAGTGAAGCCCTCATCAGCAGAA
GCGATGGGTAGTGTGCGGGCCAGCCGCTACAGCGTCGTGTCCTCCAAAGTAGATGGCCTCAAGTTGGCCACTGTGGCCGT
GTCCAATGGCCACAGCAATGGTGATGGCAAGGTGAACATGTGGCAGCCGGTGCCATGTCGTTTCGTCAAGAAGGATGGA
CACTGCAACGTGCACATCATCAACATGAGCGAGAAAGGCCAGCGCTACATAGCCGACATCTTCACCACCTGCGTGGACATC
CGTTGGCGATGGATGATAATCATCTTCTGCTTGACTTTTGTGCTTTCATGGTTGTTCTTTGGCTATGTGTTCTGGCTGGTGG
CCTTCTTCTATGGTGACTTGGGGAATAGCTCCCAGCAGTGTGTCTCCAATGTCAACAGCTTCATGGCAGCCTTCCTCTTCTCT
GTGGAGACGCAGACCACTATTGGCTATGGTTACCACCATGTGACAGAAGAGTGCCCCATCGCTGTCTTTATGGTGGTTTTC
CAGTGCATTGTTGGCTGCATCATCGACGCCTTCATCATTGGTGCCGTCATGGCCAAGATGGCCAAGCCCACGAAGCGCAAT
GAAACCCTGGTGTTTAGCCACAACGCTACAATAGCAATGCGGGACGGCAAGCTATGCCTGATGTGGCGAGTTGGCAACCT
ACGCAAAAGCCACCTGGTGGAGGCCCACGTGAGGGCTCAGCTACTCAAGTCCCGGACCACCGCCGAGGGGGAGTTTATC
CCCCTAGACCACGTAGATATTGATGTGGGCTTTGACACTGGCGTAGACCGGATCTTCCTTGTTTCCCCCATCACCATTGTCC
ATGAGATCAACGAGGACAGTCCCTTCTATGATATGAGCAAGCAGGATTTTGAGACTGCTGGATTTGAGATTGTGGTCATCC
TGGAGGGCATGGTAGAAGCCACAGCCATGACAACCCAGTGTCGCAGTTCCTACCTGGCAGGGGAGATCCTCTGGGGACA
CTGCTTCGAGCCTGTACTCTTTGAGGAGAAGAACTACTACAAGGTCGACTACTCTCATTTCCACAAAACCTACGAGGTGCC
GAGCACTCCGCTATGTAGTGCGCGGGAGCTTGCTGAAAAGAAGGATAATGAGTCCAGCTCTAACTCTTTTTGCTATGAGA
ATGAAGTGGCGATGATGGACAAAGAGGAGACGGAGGACAAAAGCGAGTGCAGCAATGATGGGAGCAGTTCACAAAAGG
CTTCAGAGTTGGGGCGCAATCTCTTCATGACGTTTAGACGAGAATCTGAGATTTGA

tsh3

ATGAATGTCCAAAACTGTTCCTCAGAAGGTTCTTCCAAAAACTTTTCCAAGGCAGCAGAAGTGAAGCCCTCATCAGCAGAA
GCGATGGGTAGTGTGCGGGCCAGCCGCTACAGCGTCGTGTCCTCCAAAGTAGATGGCCTCAAGTTGGCCACTGTGGCCGT
GTCCAATGGCCACAGCAATGGTGATGGCAAGGTGAACATGTGGCAGCCGGTGCCATGTCGTTTCGTCAAGAAGGATGGA
CACTGCAACGTGCACATCATCAACATGAGCGAGAAAGGCCAGCGCTACATAGCCGACATCTTCACCACCTGCGTGGACATC
CGTTGGCGATGGATGATAATCATCTTCTGCTTGACTTTTGTGCTTTCATGGTTGTTCTTTGGCTATGTGTTCTGGCTGGTGG
CCTTCTTCTATGGTGACTTGGGGAATAGCTCCCAGCAGTGTGTCTCCAATGTCAACAGCTTCATGGCAGCCTTCCTCTTCTCT
GTGGAGACGCAGACCACTATTGGCTATGGTTACCACCATGTGACAGAAGAGTGCCCCATCGCTGTCTTTATGGTGGTTTTC
CAGTGCATTGTTGGCTGCATCATCGACGCCTTCATCATTGGTGCCGTCATGGCCAAGATGGCCAAGCCCACGAAGCGCAAT
GAAACCCTGGTGTTTAGCCACAACGCTACAATAGCAATGCGGGACGGCAAGCTATGCCTGATGTGGCGAGTTGGCAACCT
ACGCAAAAGCCACCTGGTGGAGGCCCACGTGAGGGCTCAGCTACTCAAGTCCCGGACCACCGCCGAGGGGGAGTTTATC
CCCCTAGACCACGTAGATATTGATGTGGGCTTTGACACTGGCGTAGACCGGATCTTCCTTGTTTCCCCCATCACCATTGTCC
ATGAGATCAACGAGGACAGTCCCTTCTATGATATGAGCAAGCAGGATTTTGAGACTGCTGGATTTGAGATTGTGGTCATCC
TGGAGGGCATGGTAGAAGCCACAGCCATGACAACCCAGTGTCGCAGTTCCTACCTGGCAGGGGAGATCCTCTGGGGACA
CTGCTTCGAGCCTGTACTCTTTGAGGAGAAGAACTACTACAAGGTCGACTACTCTCATTTCCACAAAACCTACGAGGTGCC
GAGCACTCCGCTATGTAGTGCGCGGGAGCTTGCTGAAAAGAAGGATAATGAGTCCAGCTCTAACTCTTTTTGCTATGAGA
ATGAAGTGGCGATGATGGACAAAGAGGAGACGGAGGACAAAAGCGAGTGCAGCAATGATGGGAGCAGTTCACAAAAGG
CTTCAGAGTTGGGGCGCAATCTCTTCATGACGTTTAGACGAGAATCTGAGATTTGA

rhy1 ATGAATGTCCAAAACTGTTCCTCAGAAGGTTCTTCCAAAAACTTTTCCAAGGCAGCAGAAGTGAAGCCCTCATCAGCAGAA
GCGATGGGTAGTGTGCGGGCCAGCCGCTACAGCGTCGTGTCCTCCAAAGTAGATGGCCTCAAGTTGGCCACTGTGGCCGT
GTCCAATGGCCACAGCAGTGGTGATGGCAAGGTGAACATGTGGCAGCCGGTGCCATGTCGTTTCGTCAAGAAGGATGGA
CACTGCAACGTGCACATCATCAACATGAGCGAGAAAGGCCAGCGCTACATAGCCGACATCTTCACCACCTGCGTGGACATC
CGTTGGCGATGGATGATAATCATCTTCTGCTTGACTTTTGTGCTTTCATGGTTGTTCTTTGGCTATGTGTTCTGGCTGGTGG
CCTTCTTCTATGGTGACTTGGGGAATAGCTCCCAGCAGTGTGTCTCCAATGTCAACAGCTTCATGGCAGCCTTCCTCTTCTCT
GTGGAAACGCAGACCACTATTGGCTATGGTTACCACCATGTGACAGAAGAGTGCCCCATCGCTGTCTTTATGGTGGTTTTC
CAGTGCATTGTTGGCTGCATCATCAACGCCTTCATCATTGGTGCCGTCATGGCCAAGATGGCCAAGCCCACGAAGCGCAAT
GAAACCCTGGTGTTTAGCCACAACGCTACAATAGCAATGCGGGACGGTAAGCTATGCCTGATGTGGCGAGTTGGCAACCT
ACGCAAAAGCCACCTGGTGGAGGCCCACGTGAGGGCTCAGCTACTCAAGTCCCGGACCACCGCCGAGGGGGAGTTTATC
CCCCTAGACCACGTAGATATTGATGTGGGCTTTGACACTGGCGTAGACCGGATCTTCCTTGTTTCCCCCATCACCATTGTCC
ATGAGATCAACGAGGACAGTCCCTTCTATGATATGAGCAAGCAGGATTTTGAGACTGCTGGATTTGAGATTGTGGTCATCC
TGGAGGGCATGGTAGAAGCCACAGCCATGACAACCCAGTGTCGCAGTTCCTACCTGGCAGGGGAGATCCTCTGGGGACA
CTGCTTCGAGCCTGTACTCTTTGAGGAGAAGAACTACTACAAGGTCGACTACTCTCATTTCCACAAAACCTACGAGGTGCC
GAGCACTCCGCTATGTAGTGCGCGGGAGCTTGCTGAAAAGAAGGATAATGAGTCCAGCTCTAACTCTTTTTGCTATGAGA
ATGAAGTGGCGATGATGGACAAAGAGGAGACGGAGGACAAAAGCGAGTGCAGCAATGATGGGAGCAGTTCACAAAAGG
CTTCAGAGTTGGGGCGCAATCTCTTCATGACGTTTAGACGAGAATCTGAGATTTGA

rhy2

ATGAATGTCCAAAACTGTTCCTCAGAAGGTTCTTCCAAAAACTTTTCCAAGGCAGCAGAAGTGAAGCCCTCATCAGCAGAA
GCGATGGGTAGTGTGCGGGCCAGCCGCTACAGCGTCGTGTCCTCCAAAGTAGATGGCCTCAAGTTGGCCACTGTGGCCGT
GTCCAATGGCCACAGCAGTGGTGATGGCAAGGTGAACATGTGGCAGCCGGTGCCATGTCGTTTCGTCAAGAAGGATGGA
CACTGCAACGTGCACATCATCAACATGAGCGAGAAAGGCCAGCGCTACATAGCCGACATCTTCACCACCTGCGTGGACATC
CGTTGGCGATGGATGATAATCATCTTCTGCTTGACTTTTGTGCTTTCATGGTTGTTCTTTGGCTATGTGTTCTGGCTGGTGG
CCTTCTTCTATGGTGACTTGGGGAATAGCTCCCAGCAGTGTGTCTCCAATGTCAACAGCTTCATGGCAGCCTTCCTCTTCTCT
GTGGANACGCAGACCACTATTGGCTATGGTTACCACCATGTGACAGAAGAGTGCCCCATCGCTGTCTTTATGGTGGTTTTC
CAGTGCATTGTTGGCTGCATCATCAACGCCTTCATCATTGGTGCCGTCATGGCCAAGATGGCCAAGCCCACGAAGCGCAAT
GAAACCCTGGTGTTTAGCCACAACGCTACAATAGCAATGCGGGACGGTAAGCTATGCCTGATGTGGCGAGTTGGCAACCT
ACGCAAAAGCCACCTGGTGGAGGCCCACGTGAGGGCTCAGCTACTCAAGTCCCGGACCACCGCCGAGGGGGAGTTTATC
CCCCTAGACCACGTAGATATTGATGTGGGCTTTGACACTGGCGTAGACCGGATCTTCCTTGTTTCCCCCATCACCATTGTCC
ATGAGATCAACGAGGACAGTCCCTTCTATGATATGAGCAAGCAGGATTTTGAGACTGCTGGATTTGAGATTGTGGTCATCC
TGGAGGGCATGGTAGAAGCCACAGCCATGACAACCCAGTGTCGCAGTTCCTACCTGGCAGGGGAGATCCTCTGGGGACA
CTGCTTCGAGCCTGTACTCTTTGAGGAGAAGAACTACTACAAGGTCGACTACTCTCATTTCCACAAAACCTACGAGGTGCC
GAGCACTCCGCTATGTAGTGCGCGGGAGCTTGCTGAAAAGAAGGATAATGAGTCCAGCTCTAACTCTTTTTGCTATGAGA
ATGAAGTGGCGATGATGGACAAAGAGGAGACGGAGGACAAAAGCGAGTGCAGCAATGATGGGAGCAGTTCACAAAAGG
CTTCAGAGTTGGGGCGCAATCTCTTCATGACGTTTAGACGAGAATCTGAGATTTGA

rhy3

ATGAATGTCCAAAACTGTTCCTCAGAAGGTTCTTCCAAAAACTTTTCCAAGGCAGCAGAAGTGAAGCCCTCATCAGCAGAA
GCGATGGGTAGTGTGCGGGCCAGCCGCTACAGCGTCGTGTCCTCCAAAGTAGATGGCCTCAAGTTGGCCACTGTGGCCGT
GTCCAATGGCCACAGCAGTGGTGATGGCAAGGTGAACATGTGGCAGCCGGTGCCATGTCGTTTCGTCAAGAAGGATGGA
CACTGCAACGTGCACATCATCAACATGAGCGAGAAAGGCCAGCGCTACATAGCCGACATCTTCACCACCTGCGTGGACATC
CGTTGGCGATGGATGATAATCATCTTCTGCTTGACTTTTGTGCTTTCATGGTTGTTCTTTGGCTATGTGTTCTGGCTGGTGG
CCTTCTTCTATGGTGACTTGGGGAATAGCTCCCAGCAGTGTGTCTCCAATGTCAACAGCTTCATGGCAGCCTTCCTCTTCTCT
GTGGANACGCAGACCACTATTGGCTATGGTTACCACCATGTGACAGAAGAGTGCCCCATCGCTGTCTTTATGGTGGTTTTC
CAGTGCATTGTTGGCTGCATCATCAACGCCTTCATCATTGGTGCCGTCATGGCCAAGATGGCCAAGCCCACGAAGCGCAAT
GAAACCCTGGTGTTTAGCCACAACGCTACAATAGCAATGCGGGACGGTAAGCTATGCCTGATGTGGCGAGTTGGCAACCT
ACGCAAAAGCCACCTGGTGGAGGCCCACGTGAGGGCTCAGCTACTCAAGTCCCGGACCACCGCCGAGGGGGAGTTTATC
CCCCTAGACCACGTAGATATTGATGTGGGCTTTGACACTGGCGTAGACCGGATCTTCCTTGTTTCCCCCATCACCATTGTCC
ATGAGATCAACGAGGACAGTCCCTTCTATGATATGAGCAAGCAGGATTTTGAGACTGCTGGATTTGAGATTGTGGTCATCC
TGGAGGGCATGGTAGAAGCCACAGCCATGACAACCCAGTGTCGCAGTTCCTACCTGGCAGGGGAGATCCTCTGGGGACA
CTGCTTCGAGCCTGTACTCTTTGAGGAGAAGAACTACTACAAGGTCGACTACTCTCATTTCCACAAAACCTACGAGGTGCC
GAGCACTCCGCTATGTAGTGCGCGGGAGCTTGCTGAAAAGAAGGATAATGAGTCCAGCTCTAACTCTTTTTGCTATGAGA
ATGAAGTGGCGATGATGGACAAAGAGGAGACGGAGGACAAAAGCGAGTGCAGCAATGATGGGAGCAGTTCACAAAAGG
CTTCAGAGTTGGGGCGCAATCTCTTCATGACGTTTAGACGAGAATCTGAGATTTGA

rhy4

```
ATGAATGTCCAAAACTGTTCCTCAGAAGGTTCTTCCAAAAACTTTTCCAAGGCAGCAGAAGTGAAGCCCTCATCAGCAGAA
GCGATGGGTAGTGTGCGGGCCAGCCGCTACAGCGTCGTGTCCTCCAAAGTAGATGGCCTCAAGTTGGCCACTGTGGCCGT
GTCCAATGGCCACAGCAGTGGTGATGGCAAGGTGAACATGTGGCAGCCGGTGCCATGTCGTTTCGTCAAGAAGGATGGA
CACTGCAACGTGCACATCATCAACATGAGCGAGAAAGGCCAGCGCTACATAGCCGACATCTTCACCACCTGCGTGGACATC
CGTTGGCGATGGATGATAATCATCTTCTGCTTGACTTTTGTGCTTTCATGGTTGTTCTTTGGCTATGTGTTCTGGCTGGTGG
CCTTCTTCTATGGTGACTTGGGGAATAGCTCCCAGCAGTGTGTCTCCAATGTCAACAGCTTCATGGCAGCCTTCCTCTTCTCT
GTGGAAACGCAGACCACTATTGGCTATGGTTACCACCATGTGACAGAAGAGTGCCCCATCGCTGTCTTTATGGTGGTTTTC
CAGTGCATTGTTGGCTGCATCATCAACGCCTTCATCATTGGTGCCGTCATGGCCAAGATGGCCAAGCCCACGAAGCGCAAT
GAAACCCTGGTGTTTAGCCACAACGCTACAATAGCAATGCGGGACGGTAAGCTATGCCTGATGTGGCGAGTTGGCAACCT
ACGCAAAAGCCACCTGGTGGAGGCCCACGTGAGGGCTCAGCTACTCAAGTCCCGGACCACCGCCGAGGGGGAGTTTATC
CCCCTAGACCACGTAGATATTGATGTGGGCTTTGACACTGGCGTAGACCGGATCTTCCTTGTTTCCCCCATCACCATTGTCC
ATGAGATCAACGAGGACAGTCCCTTCTATGATATGAGCAAGCAGGATTTTGAGACTGCTGGATTTGAGATTGTGGTCATCC
TGGAGGGCATGGTAGAAGCCACAGCCATGACAACCCAGTGTCGCAGTTCCTACCTGGCAGGGGAGATCCTCTGGGGACA
CTGCTTCGAGCCTGTACTCTTTGAGGAGAAGAACTACTACAAGGTCGACTACTCTCATTTCCACAAAACCTACGAGGTGCC
GAGCACTCCGCTATGTAGTGCGCGGGAGCTTGCTGAAAAGAAGGATAATGAGTCCAGCTCTAACTCTTTTTGCTATGAGA
ATGAAGTGGCGATGATGGACAAAGAGGAGACGGAGGACAAAAGCGAGTGCAGCAATGATGGGAGCAGTTCACAAAAGG
CTTCAGAGTTGGGGCGCAATCTCTTCATGACGTTTAGACGAGAATCTGAGATTTGA
```

rhy5

ATGAATGTCCAAAACTGTTCCTCAGAAGGTTCTTCCAAAAACTTTTCCAAGGCAGCAGAAGTGAAGCCCTCATCAGCAGAA
GCGATGGGTAGTGTGCGGGCCAGCCGCTACAGCGTCGTGTCCTCCAAAGTAGATGGCCTCAAGTTGGCCACTGTGGCCGT
GTCCAATGGCCACAGCAGTGGTGATGGCAAGGTGAACATGTGGCAGCCGGTGCCATGTCGTTTCGTCAAGAAGGATGGA
CACTGCAACGTGCACATCATCAACATGAGCGAGAAAGGCCAGCGCTACATAGCCGACATCTTCACCACCTGCGTGGACATC
CGTTGGCGATGGATGATAATCATCTTCTGCTTGACTTTTGTGCTTTCATGGTTGTTCTTTGGCTATGTGTTCTGGCTGGTGG
CCTTCTTCTATGGTGACTTGGGGAATAGCTCCCAGCAGTGTGTCTCCAATGTCAACAGCTTCATGGCAGCCTTCCTCTTCTCT
GTGGANACGCAGACCACTATTGGCTATGGTTACCACCATGTGACAGAAGAGTGCCCCATCGCTGTCTTTATGGTGGTTTTC
CAGTGCATTGTTGGCTGCATCATCAACGCCTTCATCATTGGTGCCGTCATGGCCAAGATGGCCAAGCCCACGAAGCGCAAT
GAAACCCTGGTGTTTAGCCACAACGCTACAATAGCAATGCGGGACGGTAAGCTATGCCTGATGTGGCGAGTTGGCAACCT
ACGCAAAAGCCACCTGGTGGAGGCCCACGTGAGGGCTCAGCTACTCAAGTCCCGGACCACCGCCGAGGGGGAGTTTATC
CCCCTAGACCACGTAGATATTGATGTGGGCTTTGACACTGGCGTAGACCGGATCTTCCTTGTTTCCCCCATCACCATTGTCC
ATGAGATCAACGAGGACAGTCCCTTCTATGATATGAGCAAGCAGGATTTTGAGACTGCTGGATTTGAGATTGTGGTCATCC
TGGAGGGCATGGTAGAAGCCACAGCCATGACAACCCAGTGTCGCAGTTCCTACCTGGCAGGGGAGATCCTCTGGGGACA
CTGCTTCGAGCCTGTACTCTTTGAGGAGAAGAACTACTACAAGGTCGACTACTCTCATTTCCACAAAACCTACGAGGTGCC
GAGCACTCCGCTATGTAGTGCGCGGGAGCTTGCTGAAAAGAAGGATAATGAGTCCAGCTCTAACTCTTTTTGCTATGAGA
ATGAAGTGGCGATGATGGACAAAGAGGAGACGGAGGACAAAAGCGAGTGCAGCAATGATGGGAGCAGTTCACAAAAGG
CTTCAGAGTTGGGGCGCAATCTCTTCATGACGTTTAGACGAGAATCTGAGATTTGA
