# Supplementary material for: Gene and Allele-Specific Expression Underlying the Electric Signal Divergence in African Weakly Electric Fish
Source: Mol Biol Evol. 2024 Feb 15;41(2):msae021. doi: 10.1093/molbev/msae021 (PMC10897887; doi:10.1093/molbev/msae021)
Supplement: msae021_Supplementary_Data [file msae021_supplementary_data.zip › Cheng-MBE-efishtranscriptomes-Supplementary Table 9 KCNJ2 polyphen2.pdf]

Supplementary Table 9 Prediction of impact of two amino acid substitutions inferred from KCNJ2 transcripts of *com, tsh* and *rhy* .

Site(aa)	*com*	*tsh*	*rhy*	Score	Sensitivity	Specificity	Prediction
60	N	N	S	0.223	0.91	0.88	Benign
198	D	D	N	0.983	0.74	0.96	Probably damaging
