# Supplementary material for: Gene and Allele-Specific Expression Underlying the Electric Signal Divergence in African Weakly Electric Fish
Source: Mol Biol Evol. 2024 Feb 15;41(2):msae021. doi: 10.1093/molbev/msae021 (PMC10897887; doi:10.1093/molbev/msae021)
Supplement: msae021_Supplementary_Data [file msae021_supplementary_data.zip › Cheng-MBE-efishtranscriptomes-Supplementary Table 10 GO terms for AEI.pdf]

Supplementary Table 10 Gene Ontology terms for genes show allelic expression imbalance in F1 hybrids

Tissue	Term	GO terms	Category	Count	%	P-value	Genes	List Total	Pop Hits	Pop Total	Fold Enrichment	Bonferroni	Benjamini	FDR
EO	GO:0030239	myofibril assembly	Biological Process	2	11.76471	0.026903	*OBSCNB, CHRND*	14	38	18139	68.19172932	0.867093362	1	1
EO	GO:0006811	ion transport	Biological Process	3	17.64706	0.067548	*SCN4AA, CHRND, KCNJ2A*	14	603	18139	6.445984364	0.994345761	1	1
EO	GO:0034765	regulation of ion transmembrane transport	Biological Process	2	11.76471	0.096502	*SCN4AA, KCNJ2A*	14	141	18139	18.37791287	0.999452216	1	1
EO	GO:0005886	plasma membrane	Cellular Component	6	35.29412	0.069606	*SCN4AA, CHRND, KCNJ2A, ARL13B, DAG1, CDH15*	16	2841	18313	2.417238648	0.900608853	1	1
EO	GO:0005509	calcium ion binding	Molecular Function	3	17.64706	0.072875	*DAG1, ENPP2, CDH15*	12	701	17014	6.067760342	0.929232509	1	1
EO	GO:0005244	voltage-gated ion channel activity	Molecular Function	2	11.76471	0.081532	*SCN4AA, KCNJ2A*	12	131	17014	21.64631043	0.949037879	1	1
SM	GO:0014866	skeletal myofibril assembly	Biological Process	3	37.5	1.73E-05	*OBSCNB, MYO18AB, HSP90AA1.1*	8	17	18139	400.125	6.40E-04	6.41E-04	6.41E-04
SM	GO:0030239	myofibril assembly	Biological Process	3	37.5	8.92E-05	*OBSCNB, CHRND, HSP90AA1.1*	8	38	18139	179.0032895	0.003293307	0.001649297	0.001649
SM	GO:0060041	retina development in camera-type eye	Biological Process	2	25	0.051695	*OBSCNB, XIRP1*	8	137	18139	33.10036496	0.859693019	0.637570368	0.63757
SM	GO:0030018	Z disc	Cellular Component	3	37.5	2.99E-04	*CASQ1B, HSP90AA1.1, TRIM54*	8	70	18313	98.10535714	0.008331034	0.008364682	0.008365
SM	GO:0051015	actin filament binding	Molecular Function	2	25	0.079746	*MYO18AB, XIRP1*	7	234	17014	20.77411477	0.825391903	1	1
