# Supplementary material for: Gene and Allele-Specific Expression Underlying the Electric Signal Divergence in African Weakly Electric Fish
Source: Mol Biol Evol. 2024 Feb 15;41(2):msae021. doi: 10.1093/molbev/msae021 (PMC10897887; doi:10.1093/molbev/msae021)
Supplement: msae021_Supplementary_Data [file msae021_supplementary_data.zip › Cheng-MBE-efishtranscriptomes-Supplementary Fig. 1 Sig GO terms in up regulated genes.pdf]

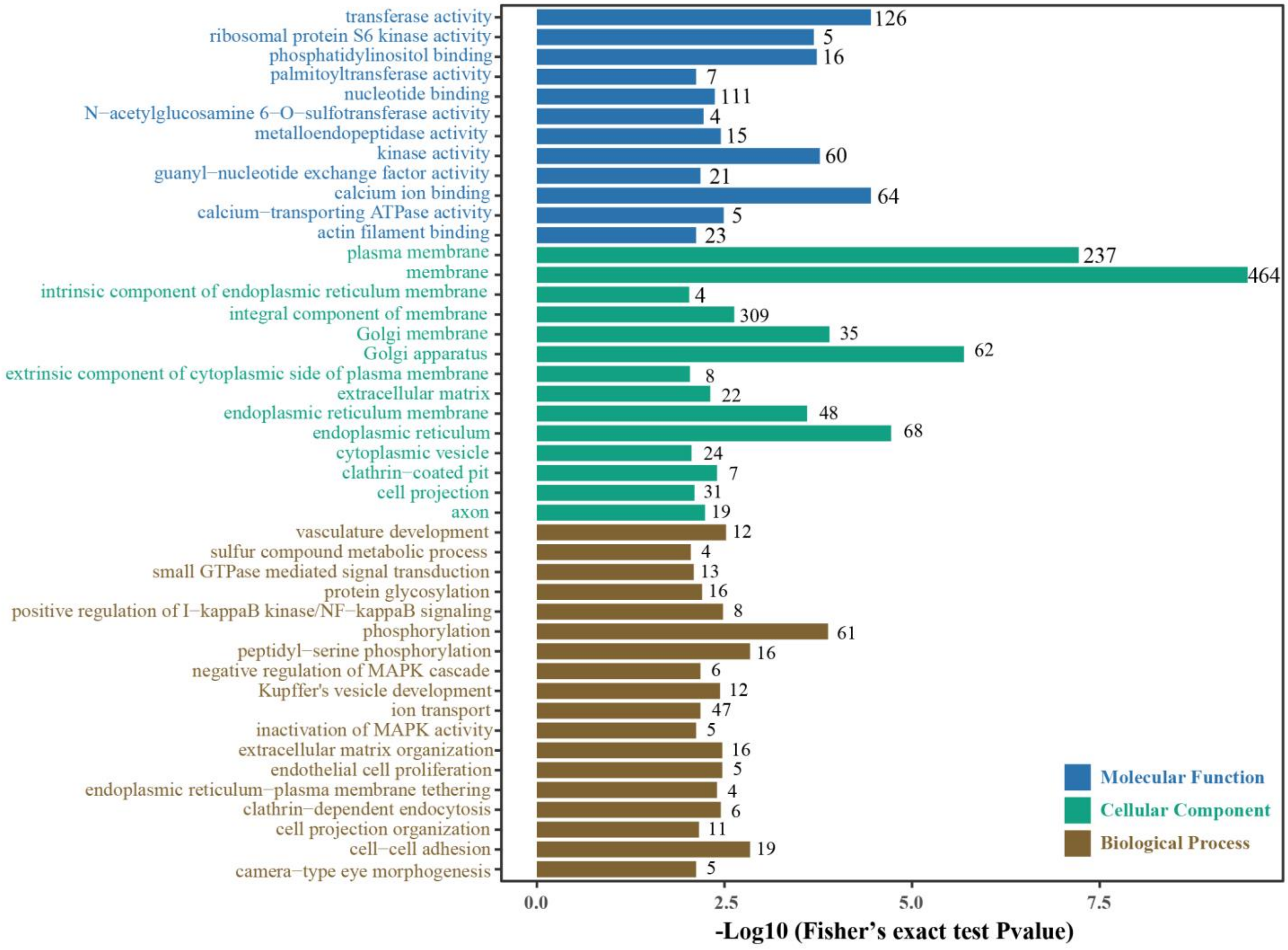


**Supplementary Fig. 1**

44 significantly enriched Gene Ontology (GO) terms with Fisher's exact test p-value < 0.01 in genes up-regulated in electric organ. The number of genes is plotted for each term. The GO terms are colored by their assignment to molecular function, cellular component, or biological process.
