# Supplementary material for: Gene and Allele-Specific Expression Underlying the Electric Signal Divergence in African Weakly Electric Fish
Source: Mol Biol Evol. 2024 Feb 15;41(2):msae021. doi: 10.1093/molbev/msae021 (PMC10897887; doi:10.1093/molbev/msae021)
Supplement: msae021_Supplementary_Data [file msae021_supplementary_data.zip › Cheng-MBE-efishtranscriptomes-Supplementary Fig. 2 Sig GO terms in down regulated genes.pdf]

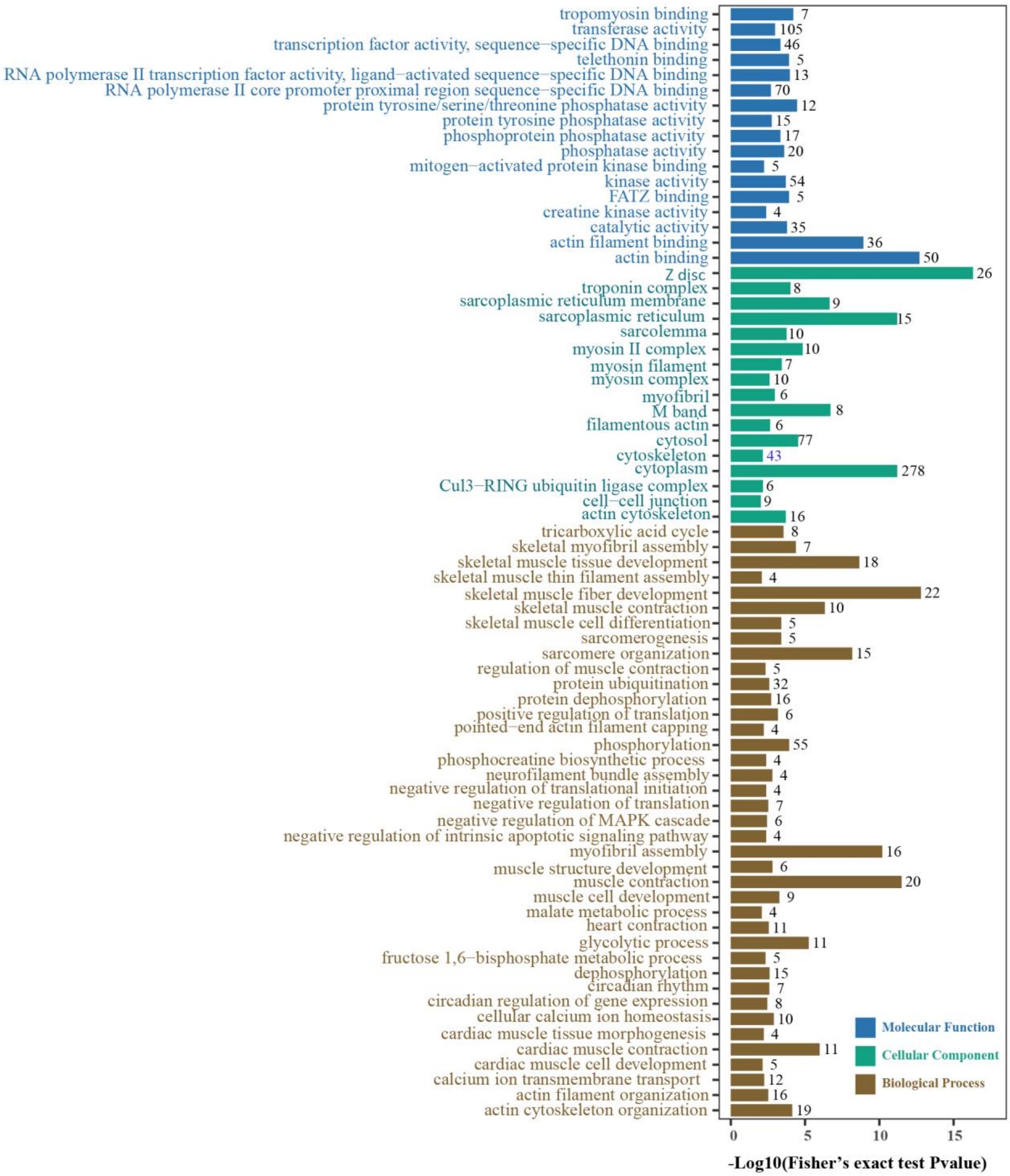

Supplementary Fig. 2

Significantly enriched Gene Ontology (GO) terms with Fisher's exact test Pvalue < 0.01 of genes down-regulated in electric organ (up regulated in skeleton muscle). The number of genes is plotted for each term. The GO terms are colored by their assignment to molecular function, cellular component, or biological process.
