# Supplementary material for: Gene and Allele-Specific Expression Underlying the Electric Signal Divergence in African Weakly Electric Fish
Source: Mol Biol Evol. 2024 Feb 15;41(2):msae021. doi: 10.1093/molbev/msae021 (PMC10897887; doi:10.1093/molbev/msae021)
Supplement: msae021_Supplementary_Data [file msae021_supplementary_data.zip › Cheng-MBE-efishtranscriptomes-Supplementary Fig. 3 All clustersfrom LRT analysis-RT.pdf]

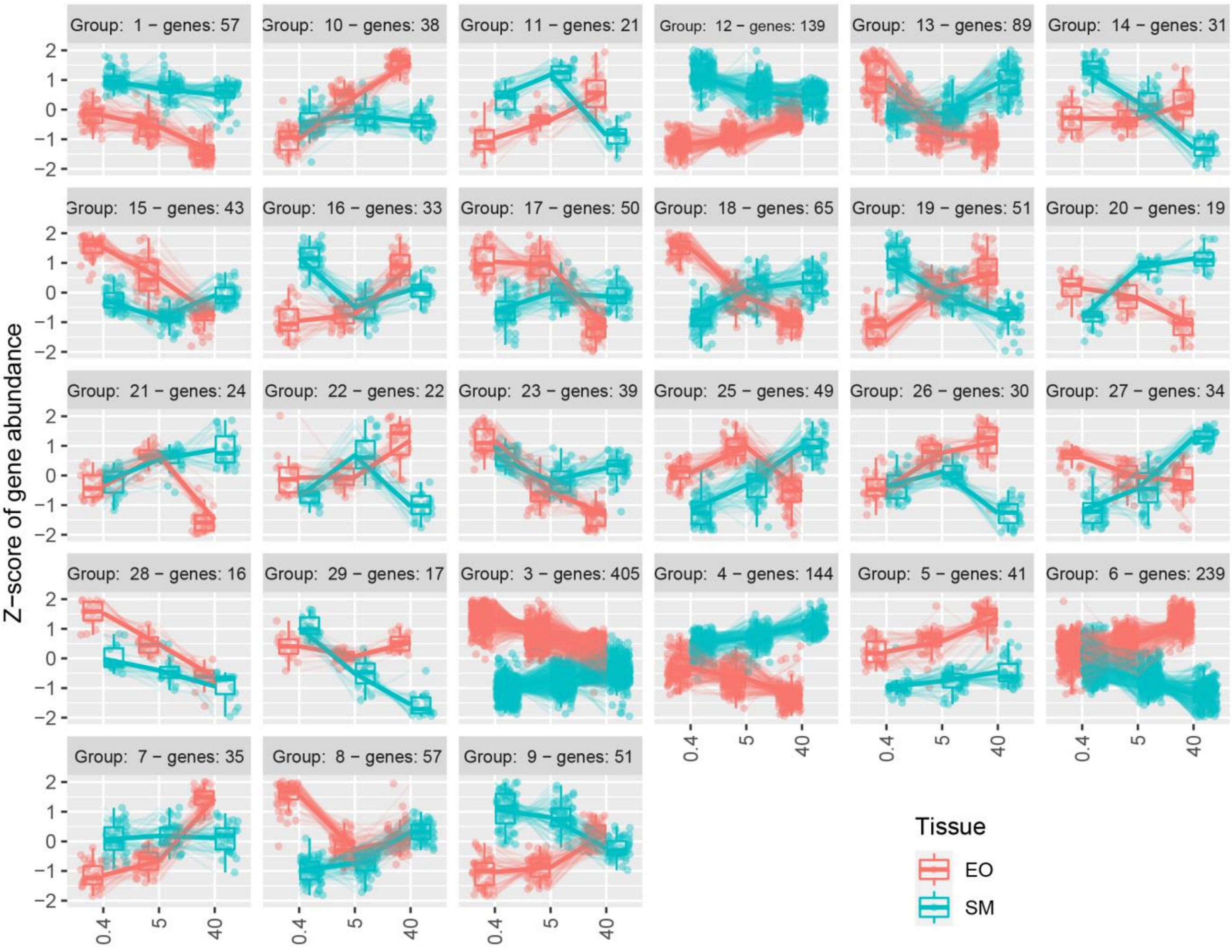

Supplementary Fig. 3

RNA-seq data clustering analysis based on EOD duration in electric organ (EO) and skeleton muscle (SM) of three F0 species. The x-axis for each group represents the EOD duration of the respective species: *C. compressirostris* (0.4ms), *C. tshokwe* (5ms) and *C. rhynchophorus* (40ms).
