# Supplementary material for: Gene and Allele-Specific Expression Underlying the Electric Signal Divergence in African Weakly Electric Fish
Source: Mol Biol Evol. 2024 Feb 15;41(2):msae021. doi: 10.1093/molbev/msae021 (PMC10897887; doi:10.1093/molbev/msae021)
Supplement: msae021_Supplementary_Data [file msae021_supplementary_data.zip › Cheng-MBE-efishtranscriptomes-Supplementary Fig. 4 Sig GO terms in group 5 6 genes.pdf]

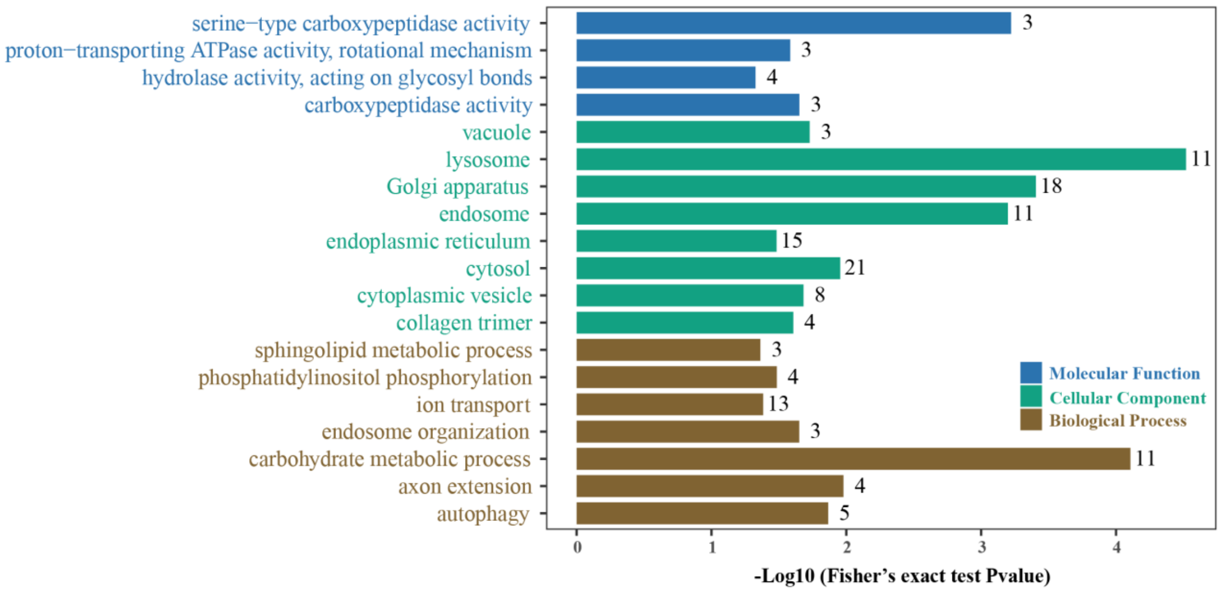

Supplementary Fig. 4

19 significantly enriched Gene Ontology (GO) terms with Fisher's exact test Pvalue < 0.05 for genes with increasing expression pattern over EOD duration change (Group 5 and 6). The number of genes is plotted for each term. The GO terms are colored by their assignment to molecular function, cellular component, or biological process.
